# The metabolic transition of liver cancer: a 34-year analysis of global burden across the 10 most populous countries, 1990–2023

**DOI:** 10.3389/fpubh.2026.1945748

**Published:** 2026-10-09

**Authors:** Qichen Luo, Sisi Lan, Jinfeng Chen, Jingru Han, Simeng Gao, Yalan Li, Haihong Deng, Yang Zhao

**Affiliations:** 1 Department of Anesthesiology, The First People's Hospital of Zhaoqing, Zhaoqing, Guangdong, China; 2 Department of Anesthesiology, The First Affiliated Hospital of Jinan University, Guangzhou, Guangdong, China; 3 Department of Oncology, The First Affiliated Hospital of Jinan University, Guangzhou, Guangdong, China

**Keywords:** behavioral risk, BMI, global burden of disease, liver cancer, metabolic risk

## Abstract

**Background:**

The global burden of liver cancer continues to rise, yet comprehensive assessments of trends in behavioral and metabolic risk factor-attributable mortality and disability-adjusted life years (DALYs) remain limited. We aimed to quantify these trends across the world's 10 most populous countries from 1990 to 2023.

**Methods:**

Using data from the Global Burden of Disease Study 2023, we extracted age-standardized mortality rates (ASMRs) and disability-adjusted life years (DALYs) for primary liver cancer attributable to seven risk factors—two composite exposures (behavioral risks and metabolic risks) and five component exposures (alcohol use, tobacco, drug use, high body mass index, and high fasting plasma glucose)—globally and across the 10 most populous nations. All burden estimates are reported with 95% uncertainty intervals (UIs). Joinpoint regression analysis identified temporal trend changes by sex and country, with annual percentage changes (APCs) and average annual percentage changes (AAPCs) reported with 95% confidence intervals (CIs).

**Findings:**

Globally, alcohol remained the predominant behavioral risk, although alcohol use-attributable ASMRs changed little across the study period [1.08 [95% UI 0.83–1.46] to 1.13 [0.87–1.46] per 100,000; AAPC 0.06% [95% CI −0.01 to 0.14], *p* = 0.07]. Concerningly, drug use-related ASMRs rose by 79% [0.40 [0.28–0.59] to 0.73 [0.56–0.92] per 100,000; AAPC 1.78% [1.68–1.87]], while metabolic risk-attributable mortality increased 71% [0.42 [0.24–0.64] to 0.73 [0.40–1.05] per 100,000; AAPC 1.67% [1.60–1.73]] and high body mass index-related DALYs nearly doubled [8.96 [3.90–14.82] to 16.26 [7.50–25.62] per 100,000]. Marked heterogeneity existed across countries: by 2023 Nigeria exhibited male metabolic risk-related DALYs nearly four times the global average [95.17 [34.23–204.38] vs. 25.13 [13.61–38.01] per 100,000]. Among women, behavioral and metabolic risk burdens increased in most countries, with the United States showing 2–3-fold higher rates than global averages.

**Interpretation:**

While traditional behavioral risk factors stabilized, metabolic risks and drug use now account for a growing share of liver cancer burden globally, with an apparent acceleration after 2020. These findings are restricted to the 10 most populous countries and therefore do not represent Europe, the Middle East, or most of Africa, and the ecological design precludes individual-level causal inference. Integrated prevention strategies targeting obesity, diabetes, and substance use disorders are nonetheless likely to be important, particularly in rapidly developing nations and among women.

## 1 Introduction

Liver cancer ranks third globally among cancer-related deaths, with an estimated 1,520,000 new cases and 1,370,000 deaths projected for 2050, imposing substantial public health burden. The epidemiological landscape has shifted dramatically: while successful hepatitis B vaccination and antiviral therapy have reduced viral hepatitis-attributable cases in many regions, metabolic dysfunction and substance use now emerge as dominant drivers (1).

This transition disproportionately affects resource-limited settings where 60% of global population resides within the 10 most populous nations—countries exhibiting vast disparities in healthcare infrastructure, socioeconomic development, and lifestyle patterns. Importantly, liver cancer represents a highly preventable malignancy: behavioral modification and metabolic risks control can substantially reduce incidence through primary and secondary prevention (1–5).

Despite the established role of behavioral and metabolic factors in hepatocarcinogenesis, comprehensive comparative analyses across diverse populations remain scarce. Previous studies examined isolated risk factors or limited geographic regions, precluding holistic understanding of the global epidemiological transition (6, 7). Moreover, the COVID-19 pandemic's impact on lifestyle factors and healthcare access necessitates updated assessment through 2023.

We therefore conducted a comprehensive analysis of liver cancer age-standardized mortality rate (ASMR) and disability-adjusted life years (DALYs) attributable to seven major risk factors (composite behavioral risks, alcohol use, tobacco, drug use, composite metabolic risks, high body mass index (BMI) and high fasting plasma glucose) across the world's 10 most populous countries from 1990 to 2023. Composite behavioral risk is the GBD aggregate of alcohol use, tobacco, and drug use, and composite metabolic risk the aggregate of high body mass index and high fasting plasma glucose; the composite exposures therefore contain their component exposures and are reported alongside, not in addition to, them. Our objective was to delineate temporal trends, identify critical inflection points, and inform evidence-based prevention strategies for this increasingly metabolic disease-driven malignancy.

## 2 Methods

This observational study on liver cancer focuses on the world's 10 most populous countries, as identified by the latest population estimates (as of June 1, 2023). Leveraging 2023 census data, this research is grounded in up-to-date population information to ensure alignment with the latest population structure and distribution characteristics. These 10 countries together account for over 60% of the global population and span a wide range of regions and levels of economic development. They were selected for population coverage rather than geographic representativeness, and, as set out in the Limitations, they do not cover Europe beyond Russia, the Middle East, or Africa beyond Nigeria.

### 2.1 Handling of the GBD data

We utilized a specialized Global Burden of Disease (GBD) Results Tool to extract age-standardized mortality rates and disability-adjusted life years related to liver cancer globally and across the world's 10 most populous countries for the period 1990–2023. Age standardization was applied to account for differences in age structures among these countries, ensuring comparability of the results. Data were disaggregated and analyzed by behavioral risk factors (e.g., alcohol, tobacco) and metabolic risk factors [e.g., high fasting plasma glucose, high body mass index (BMI)]. Trends in ASMR and DALYs attributable to these risk factors were then evaluated. Using the GBD analytical framework, which is based on the United Nations Population Division's World Population Prospects (2024 Revision), we calculated temporal changes in ASMR and DALYs by gender, country, and region for each risk factor over the 1990–2023 period.

### 2.2 Data source

The GBD database aggregates data from 204 countries and territories worldwide, encompassing over 400 health outcomes and risk factors. This database enables the monitoring of global trends in health and disease, thereby providing robust evidence to inform the development of international epidemiological prevention and treatment strategies. We extracted the age-standardized mortality rates (ASMRs) and disability-adjusted life years (DALYs) of liver cancer, which was defined using the 10th Revision of the International Classification of Diseases (ICD-10) code C22. Liver cancer mortality was estimated with the Cause of Death Ensemble model (CODEm), while exposure levels for the targeted risk factors were modeled using spatiotemporal Gaussian process regression or DisMod-MR 2.1. Risk factor-attributable burden was taken directly from the GBD comparative risk assessment framework, in which population attributable fractions are derived from exposure distributions, theoretical minimum risk exposure levels, and relative risks; we did not re-estimate attributable fractions. Every estimate is accompanied by a 95% uncertainty interval (UI), defined by the 2.5th and 97.5th percentiles of 1,000 draws from the posterior distribution, which propagates uncertainty from all modeling steps.

### 2.3 Statistical analysis

Joinpoint regression analysis was employed to examine the temporal trends in mortality rates and disability-adjusted life years for the period 1990–2023. This analytical approach enables the fitting of data across each age interval to a linear segment, with subsequent integration of these segments to form an overall trend curve. Each linear segment corresponds to a distinct phase of the trend, aligned with specific age intervals. To identify the points at which the trend exhibits significant changes, a Monte Carlo permutation test was used to assess the statistical significance of each potential joinpoint (4,499 permutations; α = 0.05). The number of joinpoints was constrained to a minimum of 0 (indicating no trend change) and a maximum of 3, ensuring the parsimony of the model. Annual percentage changes (APCs) for each segment and average annual percentage changes (AAPCs) across the full period are reported with 95% confidence intervals (CIs). Joinpoint models were fitted separately for each combination of outcome, risk factor, sex, and location, giving 462 models and 1,658 segment-specific tests in total. The permutation procedure controls the overall type I error rate within each individual model when selecting the number of joinpoints, but it does not correct for testing across models, so some individually significant inflection points are expected by chance. We therefore treat these *p*-values as descriptive rather than confirmatory and base interpretation on the magnitude and direction of change and on the width of the accompanying confidence intervals rather than on significance at any single threshold. We did not apply a false discovery rate correction, because the models are not independent—the composite exposures contain their component exposures, and mortality and DALYs are estimated from overlapping inputs—so a formal correction would not have a clear interpretation. Our substantive conclusions rest on a small number of large, internally consistent shifts that are replicated across both outcomes, both sexes, and multiple countries, rather than on isolated joinpoints.

## 3 Ethics

This study utilized an open-access database containing de-identified patient data. Since the analyzed information did not include any confidential or personally identifiable patient information, ethical approval for this study was not required in accordance with relevant research ethics guidelines.

## 4 Results

Between 1990 and 2023, liver cancer burden attributable to behavioral and metabolic risk factors demonstrated divergent trajectories globally and across the world's 10 most populous countries, collectively representing 60% of the global population (Tables 1, 2). While traditional behavioral risks stabilized or declined, metabolic risks-attributable burden nearly doubled, with a critical inflection point identified in 2020–2021 across multiple risk factors.

**Table 1:** Liver cancer age-standardized mortality rate (ASMR) attributable to behavioral and metabolic risk factors, globally and in the 10 most populous countries, 1990 and 2023, by sex.

Country	Overall LC ASMR N, all causes (1990–>2023)	1990 All risk factors-related ASMR N (95% UI), %	2023 All risk factors-related ASMR N (95% UI), %	1990 Behavioral risks-related ASMR N (95% UI), %	2023 Behavioral risks-related ASMR N (95% UI), %	1990 Alcohol -related ASMR N (95% UI), %	2023 Alcohol-related ASMR N (95% UI), %	1990 Tobacco-related ASMR N (95% UI), %	2023 Tobacco-related ASMR N (95% UI), %	1990 Drug use-related ASMR N (95% UI), %	2023 Drug use -related ASMR N (95% UI), %	1990 Metabolic risks-related ASMR N (95% UI), %	2023 Metabolic risks-related ASMR N (95% UI), %	1990 High BMI -related ASMR N (95% UI), %	2023 High BMI -related ASMR N (95% UI), %	1990 High fasting plasma glucose-related ASMR N (95% UI), %	2023 High fasting plasma glucose-related ASMR N (95% UI), %
Both																	
Global	6.04–>5.59	2.31 (1.86–2.83), 38%	2.67 (2.23–3.18), 48%	2.02 (1.55–2.47), 33%	2.21 (1.82–2.71), 40%	1.08 (0.83–1.46), 18%	1.13 (0.87–1.46), 20%	0.75 (0.24–1.25), 12%	0.57 (0.19–1.01), 10%	0.40 (0.28–0.59), 7%	0.73 (0.56–0.92), 13%	0.42 (0.24–0.64), 7%	0.73 (0.40–1.05), 13%	0.32 (0.14–0.53), 5%	0.59 (0.28–0.93), 11%	0.11 (0.07–0.16), 2%	0.15 (0.11–0.20), 3%
Indonesia	3.94–>5.08	1.42 (0.80–2.47), 36%	2.11 (1.19–3.61), 42%	1.34 (0.74–2.36), 34%	1.89 (1.05–3.32), 37%	0.72 (0.39–1.18), 18%	0.92 (0.51–1.53), 18%	0.57 (0.18–1.21), 14%	0.77 (0.23–1.57), 15%	0.22 (0.10–0.43), 5%	0.44 (0.20–0.91), 9%	0.11 (0.06–0.19), 3%	0.31 (0.14–0.55), 6%	0.05 (0.02–0.09), 1%	0.22 (0.07–0.44), 4%	0.06 (0.03–0.10), 2%	0.09 (0.05–0.15), 2%
Brazil	1.70–>1.82	0.81 (0.69–0.93), 48%	1.12 (0.96–1.28), 62%	0.70 (0.58–0.83), 41%	0.94 (0.78–1.09), 51%	0.43 (0.37–0.49), 25%	0.49 (0.43–0.56), 27%	0.23 (0.07–0.40), 13%	0.17 (0.05–0.30), 9%	0.13 (0.07–0.21), 8%	0.36 (0.20–0.51), 20%	0.17 (0.10–0.27), 10%	0.35 (0.18–0.53), 19%	0.13 (0.05–0.22), 8%	0.31 (0.13–0.49), 17%	0.05 (0.03–0.08), 3%	0.05 (0.03–0.09), 3%
China	10.14–>6.43	3.20 (2.19–4.44), 32%	2.61 (1.95–3.27), 41%	2.85 (1.80–4.12), 28%	2.21 (1.52–2.97), 34%	1.01 (0.49–2.21), 10%	0.77 (0.38–1.58), 12%	1.44 (0.46–2.41), 14%	0.91 (0.31–1.59), 14%	0.69 (0.43–1.00), 7%	0.78 (0.55–1.01), 12%	0.45 (0.29–0.68), 4%	0.56 (0.33–0.81), 9%	0.26 (0.11–0.49), 3%	0.41 (0.17–0.67), 6%	0.19 (0.14–0.28), 2%	0.16 (0.13–0.20), 2%
India	2.00–>2.76	0.82 (0.52–1.16), 41%	1.35 (0.92–1.95), 49%	0.77 (0.49–1.10), 39%	1.21 (0.81–1.76), 44%	0.49 (0.31–0.71), 24%	0.75 (0.50–1.09), 27%	0.20 (0.06–0.38), 10%	0.18 (0.05–0.37), 7%	0.16 (0.09–0.24), 8%	0.36 (0.23–0.55), 13%	0.06 (0.04–0.09), 3%	0.20 (0.12–0.33), 7%	0.01 (0.00–0.02), 1%	0.12 (0.04–0.22), 4%	0.04 (0.03–0.07), 2%	0.08 (0.05–0.13), 3%
Mexico	2.22–>2.59	1.04 (0.88–1.24), 47%	1.50 (1.29–1.77), 58%	0.80 (0.66–0.95), 36%	1.12 (0.93–1.33), 43%	0.53 (0.45–0.62), 24%	0.70 (0.59–0.83), 27%	0.23 (0.07–0.41), 11%	0.15 (0.04–0.28), 6%	0.12 (0.07–0.20), 6%	0.34 (0.21–0.50), 13%	0.36 (0.20–0.55), 16%	0.62 (0.35–0.90), 24%	0.29 (0.12–0.48), 13%	0.52 (0.24–0.82), 20%	0.08 (0.05–0.11), 3%	0.12 (0.09–0.17), 5%
Nigeria	21.04–>12.34	6.37 (2.85–10.89), 30%	4.75 (2.15–8.18), 38%	5.55 (2.48–9.51), 26%	3.66 (1.75–6.21), 30%	4.77 (2.16–8.15), 23%	3.24 (1.49–5.38), 26%	0.91 (0.18–1.87), 4%	0.38 (0.07–1.00), 3%	0.10 (0.04–0.20), 0%	0.15 (0.06–0.26), 1%	1.07 (0.37–2.17), 5%	1.50 (0.57–3.04), 12%	0.90 (0.30–1.92), 4%	1.35 (0.47–2.90), 11%	0.18 (0.06–0.39), 1%	0.17 (0.07–0.35), 1%
Pakistan	2.43–>2.88	0.94 (0.58–1.42), 39%	1.33 (0.83–2.09), 46%	0.84 (0.51–1.29), 35%	1.09 (0.65–1.81), 38%	0.40 (0.25–0.58), 17%	0.47 (0.30–0.70), 16%	0.25 (0.08–0.51), 10%	0.20 (0.05–0.45), 7%	0.28 (0.08–0.58), 12%	0.50 (0.15–1.09), 17%	0.12 (0.06–0.20), 5%	0.35 (0.16–0.59), 12%	0.06 (0.02–0.14), 3%	0.28 (0.11–0.51), 10%	0.06 (0.03–0.09), 2%	0.08 (0.05–0.12), 3%
Russia	1.77–>2.74	1.06 (0.91–1.22), 60%	1.97 (1.72–2.26), 72%	0.93 (0.80–1.09), 53%	1.75 (1.53–2.02), 64%	0.63 (0.54–0.75), 36%	1.06 (0.92–1.21), 38%	0.19 (0.06–0.32), 11%	0.32 (0.10–0.53), 12%	0.23 (0.15–0.32), 13%	0.59 (0.44–0.75), 22%	0.25 (0.12–0.39), 14%	0.54 (0.26–0.88), 20%	0.24 (0.10–0.38), 13%	0.50 (0.21–0.84), 18%	0.02 (0.01–0.03), 1%	0.05 (0.04–0.08), 2%
USA	2.02–>4.35	1.37 (1.25–1.50), 68%	3.53 (3.22–3.82), 81%	1.21 (1.08–1.34), 60%	3.07 (2.78–3.32), 71%	0.60 (0.54–0.68), 30%	1.28 (1.13–1.45), 29%	0.25 (0.08–0.41), 12%	0.45 (0.14–0.78), 10%	0.51 (0.42–0.61), 25%	1.66 (1.39–1.90), 38%	0.36 (0.21–0.52), 18%	1.20 (0.69–1.74), 28%	0.29 (0.14–0.46), 14%	1.02 (0.50–1.53), 23%	0.08 (0.06–0.10), 4%	0.24 (0.20–0.31), 5%
Bangladesh	2.59–>3.98	0.87 (0.50–1.36), 34%	1.54 (0.92–2.47), 39%	0.83 (0.47–1.28), 32%	1.32 (0.74–2.24), 33%	0.53 (0.29–0.83), 21%	0.84 (0.47–1.46), 21%	0.37 (0.12–0.67), 14%	0.52 (0.15–1.08), 13%	0.02 (0.00–0.04), 1%	0.12 (0.03–0.26), 3%	0.06 (0.03–0.12), 2%	0.29 (0.15–0.51), 7%	0.04 (0.01–0.09), 1%	0.20 (0.06–0.40), 5%	0.02 (0.01–0.04), 1%	0.09 (0.05–0.15), 2%
Male																	
Global	8.74–>8.16	3.80 (2.98–4.77), 43%	4.22 (3.51–5.17), 52%	3.52 (2.64–4.45), 40%	3.72 (3.04–4.67), 46%	1.91 (1.42–2.66), 22%	1.97 (1.52–2.62), 24%	1.45 (0.46–2.36), 17%	1.10 (0.37–1.90), 13%	0.60 (0.44–0.86), 7%	1.07 (0.84–1.33), 13%	0.49 (0.27–0.77), 6%	0.92 (0.51–1.39), 11%	0.39 (0.17–0.68), 4%	0.78 (0.36–1.22), 10%	0.11 (0.07–0.15), 1%	0.16 (0.12–0.22), 2%
Indonesia	6.15–>7.91	2.63 (1.38–4.87), 43%	3.75 (1.95–6.93), 47%	2.53 (1.34–4.69), 41%	3.50 (1.83–6.48), 44%	1.34 (0.70–2.32), 22%	1.70 (0.84–2.99), 21%	1.15 (0.37–2.47), 19%	1.56 (0.45–3.19), 20%	0.36 (0.16–0.81), 6%	0.73 (0.31–1.65), 9%	0.14 (0.07–0.26), 2%	0.40 (0.17–0.85), 5%	0.06 (0.02–0.12), 1%	0.27 (0.08–0.63), 3%	0.09 (0.04–0.16), 1%	0.13 (0.06–0.25), 2%
Brazil	1.94–>2.40	1.05 (0.90–1.23), 54%	1.58 (1.38–1.75), 66%	0.97 (0.81–1.16), 50%	1.39 (1.19–1.57), 58%	0.64 (0.55–0.76), 33%	0.84 (0.74–0.95), 35%	0.33 (0.11–0.54), 17%	0.26 (0.08–0.46), 11%	0.14 (0.07–0.23), 7%	0.44 (0.25–0.61), 18%	0.14 (0.08–0.23), 7%	0.42 (0.20–0.64), 17%	0.12 (0.05–0.20), 6%	0.38 (0.17–0.61), 16%	0.03 (0.02–0.05), 2%	0.04 (0.03–0.07), 2%
China	13.80–>9.73	4.97 (3.06–7.35), 36%	4.16 (2.90–5.52), 43%	4.65 (2.66–6.99), 34%	3.67 (2.33–5.16), 38%	1.67 (0.70–3.99), 12%	1.36 (0.60–3.02), 14%	2.67 (0.86–4.48), 19%	1.78 (0.60–3.09), 18%	0.86 (0.58–1.29), 6%	1.03 (0.73–1.35), 11%	0.47 (0.26–0.77), 3%	0.75 (0.42–1.14), 8%	0.31 (0.12–0.61), 2%	0.58 (0.24–0.98), 6%	0.16 (0.11–0.24), 1%	0.17 (0.13–0.23), 2%
India	2.67–>3.79	1.27 (0.76–1.93), 47%	2.04 (1.20–3.21), 54%	1.22 (0.73–1.86), 46%	1.89 (1.08–3.01), 50%	0.77 (0.46–1.19), 29%	1.21 (0.73–1.94), 32%	0.37 (0.11–0.71), 14%	0.35 (0.09–0.73), 9%	0.23 (0.13–0.37), 8%	0.49 (0.28–0.78), 13%	0.06 (0.03–0.10), 2%	0.22 (0.11–0.37), 6%	0.01 (0.00–0.03), 0%	0.14 (0.05–0.27), 4%	0.05 (0.03–0.08), 2%	0.09 (0.05–0.14), 2%
Mexico	2.22–>2.75	1.29 (1.08–1.50), 58%	1.93 (1.62–2.25), 70%	1.14 (0.95–1.38), 51%	1.68 (1.38–2.01), 61%	0.76 (0.64–0.90), 34%	1.05 (0.88–1.26), 38%	0.36 (0.11–0.60), 16%	0.25 (0.07–0.46), 9%	0.19 (0.11–0.30), 8%	0.53 (0.33–0.78), 19%	0.28 (0.15–0.45), 13%	0.57 (0.30–0.84), 21%	0.23 (0.10–0.40), 10%	0.49 (0.21–0.77), 18%	0.05 (0.04–0.08), 2%	0.09 (0.07–0.13), 3%
Nigeria	38.01–>25.43	11.84 (5.05–20.57), 31%	10.00 (4.43–17.69), 39%	10.48 (4.46–18.37), 28%	7.83 (3.63–13.47), 31%	9.05 (3.79–15.87), 24%	6.93 (3.13–11.57), 27%	1.69 (0.33–3.55), 4%	0.84 (0.15–2.20), 3%	0.18 (0.07–0.36), 0%	0.31 (0.12–0.54), 1%	1.79 (0.57–3.67), 5%	3.07 (1.11–6.44), 12%	1.47 (0.43–3.22), 4%	2.77 (0.95–6.12), 11%	0.33 (0.11–0.74), 1%	0.33 (0.12–0.68), 1%
Pakistan	2.49–>2.95	1.23 (0.68–2.01), 49%	1.64 (0.87–2.77), 56%	1.17 (0.65–1.93), 47%	1.49 (0.75–2.58), 50%	0.52 (0.28–0.80), 21%	0.61 (0.33–0.98), 21%	0.39 (0.13–0.81), 16%	0.34 (0.09–0.73), 12%	0.41 (0.12–0.86), 16%	0.68 (0.19–1.50), 23%	0.09 (0.04–0.16), 4%	0.28 (0.12–0.56), 10%	0.05 (0.02–0.11), 2%	0.23 (0.08–0.51), 8%	0.04 (0.02–0.06), 1%	0.05 (0.03–0.09), 2%
Russia	2.82–>4.27	1.94 (1.61–2.30), 69%	3.32 (2.80–3.91), 78%	1.83 (1.51–2.19), 65%	3.11 (2.66–3.65), 73%	1.27 (1.04–1.52), 45%	2.01 (1.70–2.39), 47%	0.50 (0.17–0.83), 18%	0.72 (0.23–1.17), 17%	0.37 (0.25–0.50), 13%	0.87 (0.68–1.11), 20%	0.30 (0.14–0.47), 11%	0.70 (0.30–1.19), 16%	0.28 (0.11–0.46), 10%	0.66 (0.26–1.15), 15%	0.02 (0.01–0.03), 1%	0.05 (0.04–0.08), 1%
USA	2.96–>6.08	2.20 (2.04–2.38), 74%	5.14 (4.77–5.49), 85%	2.02 (1.85–2.19), 68%	4.63 (4.26–4.95), 76%	1.16 (1.05–1.31), 39%	2.31 (2.07–2.59), 38%	0.41 (0.13–0.68), 14%	0.68 (0.21–1.17), 11%	0.72 (0.58–0.86), 24%	2.15 (1.80–2.47), 35%	0.48 (0.27–0.70), 16%	1.62 (0.89–2.34), 27%	0.40 (0.19–0.62), 13%	1.41 (0.67–2.15), 23%	0.09 (0.07–0.11), 3%	0.27 (0.22–0.34), 4%
Bangladesh	3.33–>5.00	1.37 (0.74–2.25), 41%	2.30 (1.20–3.91), 46%	1.33 (0.72–2.16), 40%	2.13 (1.09–3.74), 43%	0.83 (0.43–1.38), 25%	1.32 (0.67–2.43), 26%	0.65 (0.21–1.18), 19%	0.92 (0.26–1.91), 18%	0.02 (0.00–0.06), 1%	0.16 (0.04–0.37), 3%	0.06 (0.03–0.12), 2%	0.27 (0.12–0.55), 6%	0.04 (0.01–0.09), 1%	0.18 (0.06–0.45), 4%	0.03 (0.01–0.05), 1%	0.10 (0.05–0.16), 2%
Female																	
Global	3.62–>3.26	0.99 (0.81–1.25), 27%	1.28 (1.03–1.54), 39%	0.70 (0.54–0.88), 19%	0.87 (0.70–1.05), 27%	0.37 (0.29–0.48), 10%	0.37 (0.29–0.48), 11%	0.13 (0.04–0.25), 4%	0.09 (0.03–0.18), 3%	0.23 (0.14–0.34), 6%	0.44 (0.32–0.59), 13%	0.36 (0.21–0.54), 10%	0.54 (0.31–0.78), 17%	0.26 (0.11–0.43), 7%	0.42 (0.19–0.67), 13%	0.11 (0.07–0.16), 3%	0.14 (0.10–0.20), 4%
Indonesia	1.86–>2.33	0.32 (0.15–0.71), 17%	0.56 (0.26–1.05), 24%	0.25 (0.12–0.55), 13%	0.36 (0.17–0.70), 16%	0.15 (0.07–0.31), 8%	0.18 (0.09–0.34), 8%	0.03 (0.01–0.08), 2%	0.03 (0.01–0.09), 1%	0.08 (0.03–0.17), 4%	0.15 (0.07–0.36), 7%	0.08 (0.03–0.17), 4%	0.22 (0.09–0.47), 10%	0.04 (0.01–0.10), 2%	0.18 (0.06–0.39), 8%	0.04 (0.02–0.09), 2%	0.05 (0.02–0.11), 2%
Brazil	1.48–>1.34	0.61 (0.48–0.74), 41%	0.76 (0.62–0.91), 57%	0.46 (0.35–0.59), 31%	0.57 (0.45–0.70), 42%	0.24 (0.20–0.29), 16%	0.22 (0.18–0.26), 16%	0.14 (0.04–0.27), 9%	0.09 (0.03–0.17), 7%	0.12 (0.06–0.20), 8%	0.30 (0.17–0.43), 22%	0.20 (0.12–0.30), 14%	0.30 (0.17–0.45), 22%	0.15 (0.06–0.24), 10%	0.24 (0.11–0.40), 18%	0.06 (0.04–0.10), 4%	0.06 (0.04–0.10), 5%
China	6.44–>3.20	1.42 (1.01–1.94), 22%	1.09 (0.79–1.41), 34%	1.05 (0.71–1.47), 16%	0.79 (0.55–1.07), 25%	0.35 (0.26–0.46), 5%	0.19 (0.15–0.25), 6%	0.22 (0.06–0.50), 3%	0.07 (0.02–0.14), 2%	0.52 (0.26–0.85), 8%	0.54 (0.33–0.83), 17%	0.42 (0.27–0.65), 7%	0.36 (0.21–0.53), 11%	0.20 (0.08–0.37), 3%	0.23 (0.09–0.39), 7%	0.22 (0.15–0.35), 3%	0.14 (0.11–0.19), 4%
India	1.28–>1.78	0.35 (0.20–0.55), 27%	0.71 (0.42–1.15), 40%	0.30 (0.17–0.45), 23%	0.57 (0.34–0.92), 32%	0.20 (0.11–0.32), 16%	0.32 (0.18–0.52), 18%	0.03 (0.01–0.06), 2%	0.03 (0.01–0.07), 2%	0.08 (0.04–0.14), 6%	0.23 (0.13–0.39), 13%	0.05 (0.03–0.10), 4%	0.18 (0.09–0.32), 10%	0.01 (0.00–0.03), 1%	0.11 (0.04–0.19), 6%	0.04 (0.02–0.07), 3%	0.08 (0.04–0.14), 5%
Mexico	2.21–>2.44	0.82 (0.62–1.07), 37%	1.14 (0.89–1.44), 47%	0.48 (0.37–0.61), 22%	0.63 (0.50–0.80), 26%	0.32 (0.26–0.39), 14%	0.40 (0.33–0.49), 16%	0.12 (0.03–0.25), 5%	0.07 (0.02–0.12), 3%	0.06 (0.03–0.11), 3%	0.18 (0.10–0.28), 7%	0.42 (0.23–0.65), 19%	0.66 (0.38–0.96), 27%	0.34 (0.14–0.57), 15%	0.55 (0.26–0.87), 23%	0.10 (0.07–0.14), 4%	0.15 (0.11–0.20), 6%
Nigeria	2.77–>1.38	0.56 (0.30–0.96), 20%	0.41 (0.22–0.67), 30%	0.38 (0.20–0.61), 14%	0.22 (0.12–0.37), 16%	0.32 (0.17–0.54), 12%	0.20 (0.10–0.33), 14%	0.05 (0.01–0.13), 2%	0.01 (0.00–0.02), 0%	0.02 (0.01–0.03), 1%	0.02 (0.01–0.05), 2%	0.21 (0.08–0.40), 7%	0.22 (0.09–0.38), 16%	0.17 (0.06–0.35), 6%	0.18 (0.06–0.34), 13%	0.04 (0.01–0.08), 1%	0.04 (0.02–0.09), 3%
Pakistan	2.40–>2.78	0.58 (0.32–0.95), 24%	0.98 (0.52–1.64), 35%	0.44 (0.24–0.75), 18%	0.63 (0.35–1.13), 23%	0.27 (0.14–0.47), 11%	0.31 (0.17–0.51), 11%	0.06 (0.01–0.16), 2%	0.05 (0.01–0.12), 2%	0.13 (0.04–0.26), 5%	0.28 (0.10–0.61), 10%	0.16 (0.07–0.32), 7%	0.43 (0.19–0.75), 16%	0.08 (0.02–0.20), 3%	0.34 (0.12–0.63), 12%	0.08 (0.04–0.15), 3%	0.11 (0.06–0.19), 4%
Russia	1.20–>1.70	0.60 (0.48–0.73), 50%	1.08 (0.89–1.31), 63%	0.47 (0.37–0.57), 39%	0.86 (0.68–1.07), 51%	0.30 (0.25–0.38), 25%	0.43 (0.35–0.52), 25%	0.02 (0.01–0.04), 2%	0.05 (0.02–0.11), 3%	0.15 (0.09–0.23), 13%	0.41 (0.28–0.57), 24%	0.23 (0.11–0.36), 19%	0.42 (0.21–0.66), 25%	0.21 (0.09–0.34), 18%	0.38 (0.17–0.63), 23%	0.02 (0.01–0.03), 2%	0.05 (0.03–0.08), 3%
USA	1.30–>2.81	0.75 (0.62–0.88), 58%	2.10 (1.78–2.43), 75%	0.59 (0.48–0.71), 45%	1.70 (1.42–1.96), 60%	0.18 (0.14–0.22), 13%	0.37 (0.30–0.47), 13%	0.12 (0.04–0.22), 10%	0.24 (0.08–0.45), 9%	0.35 (0.27–0.45), 27%	1.23 (1.00–1.45), 44%	0.26 (0.15–0.38), 20%	0.83 (0.50–1.18), 29%	0.21 (0.09–0.33), 16%	0.67 (0.33–1.06), 24%	0.07 (0.05–0.08), 5%	0.21 (0.16–0.28), 7%
Bangladesh	1.66–>2.80	0.25 (0.13–0.44), 15%	0.67 (0.35–1.10), 24%	0.20 (0.10–0.35), 12%	0.40 (0.20–0.70), 14%	0.17 (0.08–0.30), 10%	0.29 (0.14–0.53), 10%	0.03 (0.01–0.08), 2%	0.05 (0.01–0.13), 2%	0.01 (0.00–0.01), 0%	0.07 (0.03–0.13), 2%	0.06 (0.02–0.13), 4%	0.30 (0.13–0.55), 11%	0.04 (0.01–0.10), 2%	0.22 (0.07–0.44), 8%	0.02 (0.01–0.04), 1%	0.09 (0.04–0.16), 3%

ASMR, age-standardized mortality rate; DALYs, disability-adjusted life years; LC, liver cancer; UI, uncertainty interval; USA, United States of America. Each cell reports the point estimate per 100,000 population with its 95% UI in parentheses, followed by the proportion of total liver cancer ASMR attributable to that risk factor. Behavioral risks is the GBD aggregate of alcohol use, tobacco and drug use; metabolic risks is the aggregate of high body mass index and high fasting plasma glucose. Because GBD estimates attributable burden jointly, aggregate and component exposures overlap and must not be summed. The first column reports the overall liver cancer ASMR from all causes, which is the denominator for every attributable fraction shown in the table. The next two columns report the burden attributable to all GBD risk factors combined, as estimated within the GBD comparative risk assessment; this is an attributable quantity, not a total, and it is not the sum of the risk-specific columns that follow.

**Table 2:** Liver cancer disability-adjusted life years (DALYs) attributable to behavioral and metabolic risk factors, globally and in the 10 most populous countries, 1990 and 2023, by sex.

Country	Overall LC DALYs N, all causes (1990–>2023)	1990 All risk factors-related DALYs N (95% UI), %	2023 All risk factors -related DALYs N (95% UI), %	1990 Behavioral risks-related DALYs N (95% UI), %	2023 Behavioral risks-related DALYs N (95% UI), %	1990 Alcohol-related DALYs N (95% UI), %	2023 Alcohol -related DALYs N (95% UI), %	1990 Tobacco -related DALYs N (95% UI), %	2023 Tobacco -related DALYs N (95% UI), %	1990 Drug use -related DALYs N (95% UI), %	2023 Drug use -related DALYs N (95% UI), %	1990 Metabolic risks-related DALYs N (95% UI), %	2023 Metabolic risks-related DALYs N (95% UI), %	1990 High BMI -related DALYs N (95% UI), %	2023 High BMI -related DALYs N (95% UI), %	1990 High fasting plasma glucose-related DALYs N (95% UI), %	2023 High fasting plasma glucose-related DALYs N (95% UI), %
Both																	
Global	179.6–>154.8	65.83 (52.05–82.27), 37%	70.34 (57.80–85.99), 45%	57.91 (43.70–73.36), 32%	58.12 (46.75–72.70), 38%	30.05 (22.02–42.94), 17%	29.96 (22.43–39.68), 19%	22.65 (7.18–37.12), 13%	16.31 (5.46–28.22), 11%	11.76 (8.40–16.68), 7%	17.78 (14.04–22.61), 12%	11.52 (6.49–17.72), 6%	19.29 (10.54–28.49), 12%	8.96 (3.90–14.82), 5%	16.26 (7.50–25.62), 11%	2.73 (1.87–4.05), 2%	3.53 (2.67–4.80), 2%
Indonesia	114.1–>156.2	40.67 (22.80–70.10), 36%	64.25 (36.36–110.84), 41%	38.27 (21.21–66.72), 34%	56.99 (31.96–100.98), 36%	20.23 (10.83–33.42), 18%	27.54 (15.33–45.98), 18%	15.99 (5.19–34.11), 14%	23.51 (6.92–47.95), 15%	6.59 (2.99–13.22), 6%	13.16 (5.96–27.63), 8%	3.10 (1.65–5.36), 3%	10.23 (4.39–18.49), 7%	1.58 (0.50–3.07), 1%	7.93 (2.70–15.69), 5%	1.55 (0.88–2.62), 1%	2.42 (1.34–4.26), 2%
Brazil	45.1–>46.0	21.49 (18.02–24.77), 48%	28.10 (24.38–31.54), 61%	18.59 (15.34–22.04), 41%	23.54 (19.84–27.05), 51%	10.90 (9.34–12.74), 24%	12.31 (10.77–14.09), 27%	6.13 (1.89–10.40), 14%	4.16 (1.33–7.34), 9%	3.95 (1.99–6.46), 9%	9.25 (5.31–12.67), 20%	4.56 (2.63–7.00), 10%	8.80 (4.54–13.39), 19%	3.50 (1.41–5.74), 8%	7.77 (3.41–12.34), 17%	1.17 (0.70–1.97), 3%	1.25 (0.81–2.01), 3%
China	314.5–>189.9	99.03 (67.17–138.34), 31%	76.25 (54.57–97.40), 40%	88.28 (54.22–128.44), 28%	64.61 (42.23–88.60), 34%	32.33 (13.97–73.08), 10%	24.22 (10.18–51.63), 13%	45.52 (14.71–75.85), 14%	28.38 (9.67–48.43), 15%	19.72 (12.57–29.28), 6%	19.63 (13.95–24.81), 10%	13.79 (8.57–21.68), 4%	16.61 (9.28–25.05), 9%	8.50 (3.39–15.71), 3%	13.06 (5.44–21.51), 7%	5.47 (3.80–7.98), 2%	3.82 (3.01–4.87), 2%
India	56.1–>75.7	22.29 (13.97–31.73), 40%	35.88 (24.16–51.67), 47%	21.10 (13.15–30.21), 38%	32.01 (21.51–46.48), 42%	13.06 (8.19–18.85), 23%	19.88 (12.82–28.91), 26%	5.53 (1.70–10.50), 10%	4.78 (1.25–9.98), 6%	4.56 (2.82–6.97), 8%	9.44 (6.09–14.67), 13%	1.41 (0.86–2.33), 3%	5.47 (2.94–8.98), 7%	0.37 (0.14–0.73), 1%	3.63 (1.35–6.72), 5%	1.05 (0.64–1.70), 2%	1.96 (1.18–3.19), 3%
Mexico	55.7–>63.1	25.83 (22.03–30.63), 46%	36.67 (31.71–43.01), 58%	19.79 (16.60–23.43), 36%	27.67 (23.12–32.78), 44%	12.89 (11.01–15.12), 23%	17.12 (14.49–19.99), 27%	5.56 (1.63–9.63), 10%	3.57 (1.05–6.46), 6%	3.58 (2.07–5.68), 6%	8.95 (5.68–13.13), 14%	9.02 (4.93–14.20), 16%	15.16 (8.56–22.01), 24%	7.55 (3.21–12.56), 14%	13.02 (5.97–20.34), 21%	1.74 (1.18–2.65), 3%	2.78 (2.09–3.65), 4%
Nigeria	684.5–>366.9	195.08 (86.03–339.03), 28%	133.16 (62.05–224.03), 36%	167.53 (74.68–282.79), 24%	99.45 (48.34–168.64), 27%	142.84 (64.49–245.31), 21%	87.19 (41.44–144.61), 24%	28.23 (5.55–57.76), 4%	11.05 (1.97–28.87), 3%	3.22 (1.25–6.43), 0%	4.32 (1.82–7.46), 1%	35.99 (12.42–73.83), 5%	46.17 (17.27–96.29), 13%	31.57 (10.19–66.68), 5%	42.68 (14.92–93.08), 12%	4.64 (1.65–10.34), 1%	3.97 (1.55–8.08), 1%
Pakistan	70.7–>86.2	27.77 (17.27–42.69), 39%	40.30 (25.04–63.87), 47%	25.21 (15.35–39.24), 36%	32.73 (19.19–55.70), 38%	11.82 (7.40–17.31), 17%	14.22 (9.02–20.80), 17%	6.67 (2.09–13.64), 9%	5.74 (1.48–12.57), 7%	9.19 (2.79–19.19), 13%	15.24 (4.71–33.28), 18%	3.35 (1.80–5.67), 5%	11.03 (5.00–18.73), 13%	1.96 (0.65–4.14), 3%	9.14 (3.39–16.50), 11%	1.44 (0.85–2.26), 2%	2.15 (1.29–3.20), 2%
Russia	53.0–>72.3	29.12 (24.94–33.53), 55%	50.77 (44.23–58.41), 70%	25.91 (22.00–30.25), 49%	45.57 (39.84–52.68), 63%	17.09 (14.47–20.47), 32%	27.61 (23.85–31.88), 38%	5.74 (1.92–9.60), 11%	9.53 (3.04–15.63), 13%	6.49 (4.61–8.73), 12%	14.68 (11.40–18.41), 20%	6.76 (3.25–10.49), 13%	13.55 (6.33–22.21), 19%	6.36 (2.74–10.15), 12%	12.70 (5.37–21.55), 18%	0.49 (0.30–0.74), 1%	1.07 (0.76–1.68), 1%
USA	52.4–>108.2	36.27 (33.10–39.64), 69%	86.61 (80.45–93.35), 80%	32.36 (29.40–35.59), 62%	75.79 (69.14–81.23), 70%	15.40 (13.82–17.48), 29%	32.26 (28.51–37.31), 30%	6.96 (2.25–11.55), 13%	11.44 (3.57–19.80), 11%	14.49 (12.02–17.11), 28%	40.33 (34.45–45.79), 37%	9.15 (5.16–13.54), 17%	29.91 (17.01–43.18), 28%	7.79 (3.61–12.13), 15%	25.91 (12.72–38.35), 24%	1.60 (1.23–2.13), 3%	5.30 (4.36–6.78), 5%
Bangladesh	77.5–>112.3	25.67 (14.74–39.53), 33%	43.53 (25.27–70.60), 39%	24.31 (14.01–37.71), 31%	37.16 (20.86–62.25), 33%	15.75 (8.52–24.70), 20%	23.69 (13.01–40.04), 21%	10.82 (3.60–19.04), 14%	14.05 (4.12–30.67), 12%	0.52 (0.02–1.31), 1%	3.68 (0.89–8.36), 3%	1.76 (0.91–3.33), 2%	8.36 (4.17–14.91), 8%	1.17 (0.39–2.64), 2%	6.07 (2.03–12.11), 5%	0.60 (0.29–1.07), 1%	2.43 (1.31–3.83), 2%
Male																	
Global	261.1–>228.2	108.29 (82.51–137.45), 41%	111.50 (90.74–142.07), 49%	100.28 (74.01–129.25), 38%	97.53 (78.21–125.35), 43%	52.44 (37.03–78.03), 20%	52.12 (38.72–72.41), 23%	43.15 (13.85–69.70), 17%	31.13 (10.52–53.00), 14%	17.43 (12.91–24.53), 7%	25.63 (20.59–32.39), 11%	13.71 (7.37–21.66), 5%	25.13 (13.61–38.01), 11%	11.11 (4.83–19.16), 4%	21.73 (10.04–34.50), 10%	2.73 (1.83–4.15), 1%	3.86 (2.84–5.00), 2%
Indonesia	176.9–>242.5	74.30 (39.14–136.93), 42%	112.88 (59.62–210.69), 47%	71.45 (37.76–131.62), 40%	104.68 (55.07–195.58), 43%	37.38 (19.66–65.38), 21%	50.33 (25.55–88.85), 21%	32.20 (10.40–68.84), 18%	46.80 (13.74–96.43), 19%	11.15 (4.80–25.21), 6%	22.06 (9.50–50.79), 9%	4.07 (1.94–7.42), 2%	13.15 (5.28–28.31), 5%	1.85 (0.59–4.05), 1%	9.77 (2.92–23.02), 4%	2.24 (1.07–4.11), 1%	3.51 (1.74–6.69), 1%
Brazil	52.6–>60.7	27.67 (23.59–32.45), 53%	38.98 (34.53–43.16), 64%	25.59 (21.49–30.53), 49%	34.22 (29.65–38.43), 56%	16.39 (13.89–19.56), 31%	20.51 (18.07–23.34), 34%	8.84 (2.87–14.40), 17%	6.34 (2.01–10.88), 10%	4.21 (2.15–6.71), 8%	10.91 (6.50–14.75), 18%	3.92 (2.08–6.13), 7%	10.56 (5.15–16.45), 17%	3.21 (1.25–5.53), 6%	9.74 (4.31–15.83), 16%	0.76 (0.45–1.35), 1%	0.97 (0.63–1.57), 2%
China	436.6–>299.6	155.81 (93.77–229.59), 36%	125.87 (84.71–167.86), 42%	145.51 (80.90–219.33), 33%	110.16 (66.51–156.64), 37%	54.04 (19.18–129.88), 12%	43.54 (16.35–98.31), 15%	84.12 (27.37–140.16), 19%	55.18 (18.85–93.93), 18%	24.77 (17.34–36.04), 6%	26.32 (19.07–34.11), 9%	15.03 (8.29–25.03), 3%	23.96 (12.76–37.22), 8%	10.55 (4.04–20.35), 2%	19.84 (8.22–33.18), 7%	4.59 (2.95–6.80), 1%	4.40 (3.40–5.86), 1%
India	75.6–>106.2	34.49 (20.46–51.44), 46%	54.16 (33.05–85.66), 51%	33.32 (19.80–49.81), 44%	50.09 (30.07–80.02), 47%	20.45 (12.37–31.22), 27%	32.29 (19.34–51.87), 30%	10.07 (3.12–19.29), 13%	9.10 (2.41–19.34), 9%	6.61 (3.78–10.67), 9%	12.78 (7.38–19.75), 12%	1.51 (0.83–2.46), 2%	6.22 (3.09–11.01), 6%	0.40 (0.15–0.82), 1%	4.26 (1.55–8.45), 4%	1.12 (0.62–1.80), 1%	2.05 (1.17–3.39), 2%
Mexico	56.1–>68.7	31.62 (26.61–36.79), 56%	47.31 (39.68–55.43), 69%	28.17 (23.54–33.36), 50%	41.58 (33.96–49.68), 61%	18.42 (15.55–22.03), 33%	25.72 (21.32–30.99), 37%	8.43 (2.59–14.02), 15%	5.93 (1.67–10.79), 9%	5.37 (3.15–8.60), 10%	13.71 (8.75–19.88), 20%	7.15 (3.75–11.62), 13%	14.29 (7.57–21.09), 21%	6.08 (2.52–10.70), 11%	12.54 (5.50–19.61), 18%	1.21 (0.79–1.85), 2%	2.18 (1.53–3.00), 3%
Nigeria	1190.3–>759.9	345.91 (148.24–607.09), 29%	282.51 (126.10–487.56), 37%	300.84 (129.46–518.77), 25%	214.05 (101.02–367.88), 28%	257.37 (109.58–444.20), 22%	187.69 (87.11–313.16), 25%	50.37 (9.77–104.09), 4%	24.47 (4.31–63.87), 3%	5.39 (1.95–11.05), 0%	8.81 (3.42–15.56), 1%	59.41 (18.95–124.83), 5%	95.17 (34.23–204.38), 13%	51.62 (15.46–111.53), 4%	88.37 (30.50–196.67), 12%	8.12 (2.58–18.32), 1%	7.71 (2.83–16.51), 1%
Pakistan	75.7–>90.0	37.79 (21.38–61.84), 50%	50.10 (26.31–84.60), 56%	36.10 (20.35–59.54), 48%	45.25 (22.71–78.52), 50%	15.76 (8.91–24.56), 21%	18.71 (10.20–29.95), 21%	10.81 (3.40–21.74), 14%	9.62 (2.63–20.85), 11%	13.80 (4.15–27.92), 18%	21.27 (5.70–47.35), 24%	2.73 (1.26–5.17), 4%	9.03 (3.67–17.97), 10%	1.74 (0.57–3.81), 2%	7.70 (2.60–16.64), 9%	1.02 (0.52–1.62), 1%	1.46 (0.81–2.48), 2%
Russia	80.3–>112.8	51.07 (42.45–60.47), 64%	85.09 (71.97–98.67), 75%	48.37 (39.47–57.76), 60%	79.68 (67.80–92.54), 71%	32.87 (26.83–39.89), 41%	51.54 (43.67–60.05), 46%	13.89 (4.68–22.85), 17%	19.97 (6.47–32.56), 18%	10.10 (7.13–13.52), 13%	21.61 (17.38–27.66), 19%	7.91 (3.59–12.54), 10%	18.23 (7.71–30.84), 16%	7.45 (3.07–12.19), 9%	17.28 (6.75–30.17), 15%	0.51 (0.31–0.77), 1%	1.13 (0.79–1.75), 1%
USA	76.1–>151.0	56.77 (52.79–61.30), 75%	126.07 (117.93–134.79), 83%	52.34 (48.18–57.04), 69%	113.70 (105.10–121.92), 75%	28.98 (26.09–32.82), 38%	57.42 (50.98–64.87), 38%	11.17 (3.66–18.44), 15%	17.13 (5.34–29.11), 11%	19.88 (16.62–23.77), 26%	52.10 (44.12–60.46), 34%	12.43 (6.99–18.36), 16%	40.58 (22.15–58.44), 27%	10.77 (5.02–16.68), 14%	35.92 (17.23–54.20), 24%	1.93 (1.52–2.52), 3%	6.15 (5.01–7.84), 4%
Bangladesh	102.5–>145.4	41.14 (22.56–66.11), 40%	66.25 (34.13–116.49), 46%	39.88 (22.00–64.17), 39%	60.95 (31.27–106.53), 42%	24.95 (12.83–41.00), 24%	37.98 (19.42–69.73), 26%	19.11 (6.41–33.34), 19%	25.54 (7.37–55.71), 18%	0.81 (0.03–2.06), 1%	5.34 (1.03–12.60), 4%	1.88 (0.89–3.86), 2%	8.21 (3.55–16.70), 6%	1.20 (0.43–2.84), 1%	5.73 (1.83–14.10), 4%	0.68 (0.31–1.28), 1%	2.58 (1.25–4.49), 2%
Female																	
Global	101.7–>84.4	26.16 (21.07–33.01), 26%	31.77 (25.38–39.64), 38%	18.41 (14.34–23.32), 18%	21.30 (17.53–25.91), 25%	9.46 (7.19–12.46), 9%	9.26 (7.07–12.43), 11%	3.26 (0.92–6.40), 3%	2.25 (0.70–4.08), 3%	6.45 (4.10–9.69), 6%	10.65 (7.79–14.18), 13%	9.42 (5.49–14.47), 9%	13.72 (7.67–19.66), 16%	6.88 (2.85–11.62), 7%	11.03 (4.99–17.14), 13%	2.73 (1.85–4.19), 3%	3.21 (2.30–4.51), 4%
Indonesia	53.8–>69.9	9.07 (4.25–20.29), 17%	16.89 (7.91–32.49), 24%	7.09 (3.25–15.78), 13%	10.55 (5.01–20.14), 15%	4.14 (2.03–8.75), 8%	5.36 (2.64–10.14), 8%	0.78 (0.13–1.99), 1%	0.84 (0.14–2.43), 1%	2.27 (0.79–5.31), 4%	4.47 (1.93–10.42), 6%	2.20 (0.96–5.02), 4%	7.35 (2.84–15.73), 11%	1.32 (0.36–3.12), 2%	6.09 (1.97–14.01), 9%	0.91 (0.39–2.27), 2%	1.37 (0.60–2.79), 2%
Brazil	38.1–>33.4	15.95 (12.57–19.45), 42%	18.99 (15.75–22.36), 57%	12.34 (9.43–15.80), 32%	14.62 (11.54–17.60), 44%	6.01 (4.87–7.29), 16%	5.42 (4.55–6.52), 16%	3.69 (1.07–7.01), 10%	2.33 (0.73–4.35), 7%	3.72 (1.88–6.11), 10%	7.90 (4.54–11.13), 24%	5.11 (2.98–7.63), 13%	7.28 (4.12–10.99), 22%	3.74 (1.44–6.37), 10%	6.08 (2.62–10.06), 18%	1.53 (0.88–2.53), 4%	1.48 (0.93–2.37), 4%
China	185.3–>79.0	39.62 (28.21–54.09), 21%	26.38 (19.58–33.98), 33%	28.51 (19.65–39.35), 15%	18.98 (13.55–25.29), 24%	9.53 (6.94–12.46), 5%	4.70 (3.54–5.92), 6%	5.22 (1.32–12.03), 3%	1.57 (0.50–3.16), 2%	14.53 (7.43–24.15), 8%	13.11 (8.39–19.36), 17%	12.35 (8.07–19.12), 7%	9.03 (5.32–13.33), 11%	6.25 (2.54–11.42), 3%	6.09 (2.48–10.10), 8%	6.33 (4.14–9.96), 3%	3.22 (2.44–4.47), 4%
India	34.5–>45.7	9.12 (5.31–14.26), 26%	18.30 (10.75–29.17), 40%	7.92 (4.56–12.16), 23%	14.66 (8.76–23.44), 32%	5.12 (2.84–8.13), 15%	7.94 (4.40–12.73), 17%	0.64 (0.15–1.54), 2%	0.64 (0.13–1.51), 1%	2.31 (1.17–4.05), 7%	6.28 (3.41–10.94), 14%	1.30 (0.72–2.30), 4%	4.69 (2.40–7.89), 10%	0.33 (0.10–0.75), 1%	2.98 (1.10–5.40), 7%	0.98 (0.52–1.70), 3%	1.86 (0.96–3.49), 4%
Mexico	55.4–>58.0	20.52 (15.59–26.79), 37%	27.37 (21.59–34.51), 47%	12.10 (9.31–15.18), 22%	15.53 (12.13–19.77), 27%	7.82 (6.28–9.49), 14%	9.62 (7.77–11.97), 17%	2.94 (0.71–6.15), 5%	1.52 (0.49–2.80), 3%	1.91 (0.99–3.11), 3%	4.80 (2.63–7.47), 8%	10.74 (5.84–16.54), 19%	15.89 (9.18–22.97), 27%	8.91 (3.75–14.72), 16%	13.41 (6.51–20.84), 23%	2.23 (1.46–3.35), 4%	3.30 (2.40–4.48), 6%
Nigeria	80.9–>39.4	15.26 (8.24–25.81), 19%	11.02 (5.96–17.99), 28%	10.15 (5.49–16.57), 13%	5.93 (3.24–10.00), 15%	8.71 (4.81–14.34), 11%	5.15 (2.72–8.51), 13%	1.09 (0.24–3.03), 1%	0.16 (0.03–0.49), 0%	0.50 (0.21–0.89), 1%	0.64 (0.31–1.23), 2%	5.85 (2.25–11.23), 7%	5.99 (2.41–10.23), 15%	5.02 (1.65–9.90), 6%	5.24 (1.90–9.49), 13%	0.89 (0.34–1.98), 1%	0.88 (0.37–2.07), 2%
Pakistan	65.7–>81.4	16.13 (8.78–26.53), 25%	29.09 (15.34–48.91), 36%	12.46 (6.94–21.59), 19%	18.49 (10.28–32.91), 23%	7.26 (4.01–12.63), 11%	9.12 (4.85–14.94), 11%	1.58 (0.31–4.39), 2%	1.29 (0.20–3.44), 2%	3.93 (1.18–7.84), 6%	8.39 (2.93–18.20), 10%	4.19 (1.97–8.04), 6%	13.26 (5.81–23.17), 16%	2.27 (0.62–5.39), 3%	10.74 (3.93–19.98), 13%	1.99 (1.05–3.47), 3%	2.93 (1.49–5.11), 4%
Russia	35.6–>41.9	15.86 (12.33–19.45), 45%	25.58 (21.29–31.00), 61%	12.36 (9.72–15.16), 35%	20.70 (16.48–25.24), 49%	7.75 (6.26–9.71), 22%	10.26 (8.19–12.47), 24%	0.58 (0.17–1.25), 2%	1.72 (0.51–3.15), 4%	4.27 (2.57–6.32), 12%	9.63 (6.79–12.98), 23%	6.03 (2.92–9.41), 17%	9.92 (4.96–15.68), 24%	5.65 (2.46–9.00), 16%	9.15 (4.15–14.98), 22%	0.48 (0.29–0.77), 1%	1.01 (0.70–1.64), 2%
USA	32.8–>68.8	19.35 (16.16–22.69), 59%	50.41 (43.30–58.41), 73%	15.91 (13.20–18.80), 49%	41.04 (34.35–46.98), 60%	4.30 (3.49–5.50), 13%	9.16 (7.38–12.61), 13%	3.40 (1.04–6.03), 10%	6.20 (1.95–11.27), 9%	9.97 (7.93–12.42), 30%	29.59 (24.34–34.52), 43%	6.34 (3.55–9.43), 19%	20.03 (11.95–28.78), 29%	5.22 (2.37–8.27), 16%	16.64 (8.25–25.86), 24%	1.34 (1.00–1.81), 4%	4.52 (3.60–6.15), 7%
Bangladesh	46.8–>74.8	6.98 (3.57–12.00), 15%	18.22 (9.87–29.29), 24%	5.49 (2.71–9.64), 12%	10.69 (5.27–18.73), 14%	4.64 (2.31–8.26), 10%	7.79 (3.78–13.23), 10%	0.77 (0.17–1.86), 2%	1.16 (0.25–2.77), 2%	0.17 (0.01–0.46), 0%	1.90 (0.68–3.88), 3%	1.63 (0.65–3.47), 4%	8.52 (3.59–15.40), 11%	1.14 (0.31–2.80), 2%	6.46 (1.99–12.48), 9%	0.51 (0.25–0.95), 1%	2.27 (1.16–4.02), 3%

DALYs, disability-adjusted life years; LC, liver cancer; UI, uncertainty interval; USA, United States of America. Each cell reports the point estimate per 100,000 population with its 95% UI in parentheses, followed by the proportion of total liver cancer DALYs attributable to that risk factor. Behavioral risks is the GBD aggregate of alcohol use, tobacco and drug use; metabolic risks is the aggregate of high body mass index and high fasting plasma glucose. Because GBD estimates attributable burden jointly, aggregate and component exposures overlap and must not be summed. The first column reports the overall liver cancer DALYs from all causes, which is the denominator for every attributable fraction shown in the table. The next two columns report the burden attributable to all GBD risk factors combined, as estimated within the GBD comparative risk assessment; this is an attributable quantity, not a total, and it is not the sum of the risk-specific columns that follow.

### 4.1 Behavioral risks: diverging trajectories

Global ASMR for overall behavioral risks (alcohol, tobacco, and drug use combined) showed no net decline across the study period [2.02 [95% UI 1.55–2.47] to 2.21 [1.82–2.71] per 100,000; AAPC 0.19% [95% CI 0.12–0.28]], rising to 1996 [APC 1.52% [0.86–2.59]] and declining only modestly thereafter [APC −0.10% [−0.19 to −0.03], 1996–2023] (Supplementary Figure S1), yet this masked substantial heterogeneity across individual risk factors and sex-specific patterns (Figure 1). Among males, only China and Nigeria showed lower behavioral risk-attributable ASMR in 2023 compared to 1990, though Nigeria maintained rates exceeding twice the global average throughout [10.48 [95% UI 4.46–18.37] to 7.83 [3.63–13.47] per 100,000]. Conversely, among females, behavioral risks-related ASMR increased globally with accelerating trends in recent years [APC 1.59% [95% CI 0.63–3.50], 1990–1995; 0.31% [−0.54 to 0.39], 1995–2021, not significant; 2.98% [0.42–4.37], 2021–2023]. By 2023, U.S. women exhibited behavioral risks ASMR more than twice the global average, driven predominantly by drug use. DALYs mirrored these patterns (Supplementary Figure S2), with recent increases evident in six of 10 countries among males (India, Indonesia, Pakistan, Nigeria, Bangladesh, and Russia) and in six of 10 among females (India, Indonesia, Pakistan, Nigeria, Bangladesh, and the United States). Bangladesh demonstrated the steepest female DALY escalation [APC 10.91% [95% CI 7.76–15.80], 2020–2023].

**Figure 1:**
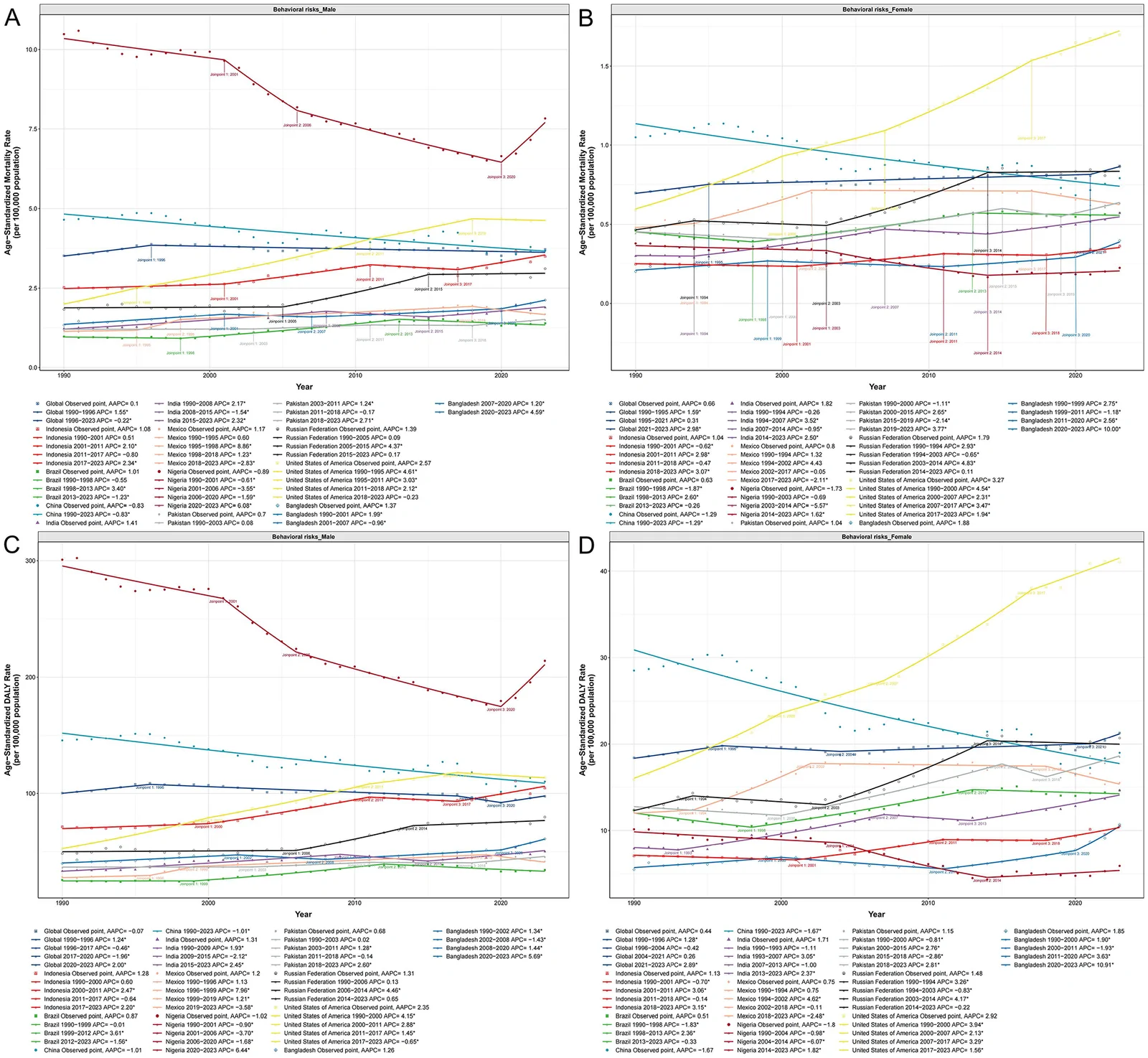
Male/female age-standardized mortality rates and age-standardized DALYs trends for behavioral risks. All indices are per 100, 000 population. ASMR, Age Standardized Mortality Rate; DALYs, Disability Adjusted Life Years; AAPC, Average Annual Percentage Change; APC, Annual Percentage Change.

### 4.2 Alcohol use

Alcohol use-attributable liver cancer ASMR remained globally stable from 1990 to 2023 [1.08 [95% UI 0.83–1.46] to 1.13 [0.87–1.46] per 100,000; AAPC 0.06% [95% CI −0.01 to 0.14], *p* = 0.07] (Supplementary Figure S3), yet eight of 10 countries experienced increases while only China and Nigeria declined (Figure 2). The United States demonstrated the most substantial rise [0.60 [95% UI 0.54–0.68] to 1.28 [1.13–1.45] per 100,000; 114% increase], whereas Nigeria, despite declining from 9.05 [95% UI 3.79–15.87] to 6.93 [3.13–11.57] per 100,000 among males, maintained the highest absolute rates globally throughout the study period. Among females, Russia reached the highest ASMR by 2023 [0.43 [95% UI 0.35–0.52] per 100,000], surpassing China's 1990 level [0.35 [0.26–0.46] per 100,000]. Sex-stratified analysis revealed concerning recent increases in six countries among males (China, Indonesia, India, Nigeria, Pakistan, Bangladesh), with Nigeria showing the steepest acceleration [APC 5.89% [95% CI 4.02–9.33], 2020–2023]. Among females, ASMR increased in recent years in five countries (India, Indonesia, Nigeria, Pakistan, and Bangladesh), with no recent increase in Brazil, China, Mexico, Russia, or the United States. DALYs paralleled mortality trends (Supplementary Figure S4), with Bangladesh recording the highest female growth rate [APC 7.38% [95% CI 4.80–11.79], 2019–2023], potentially reflecting pandemic-associated increases in harmful alcohol consumption.

**Figure 2:**
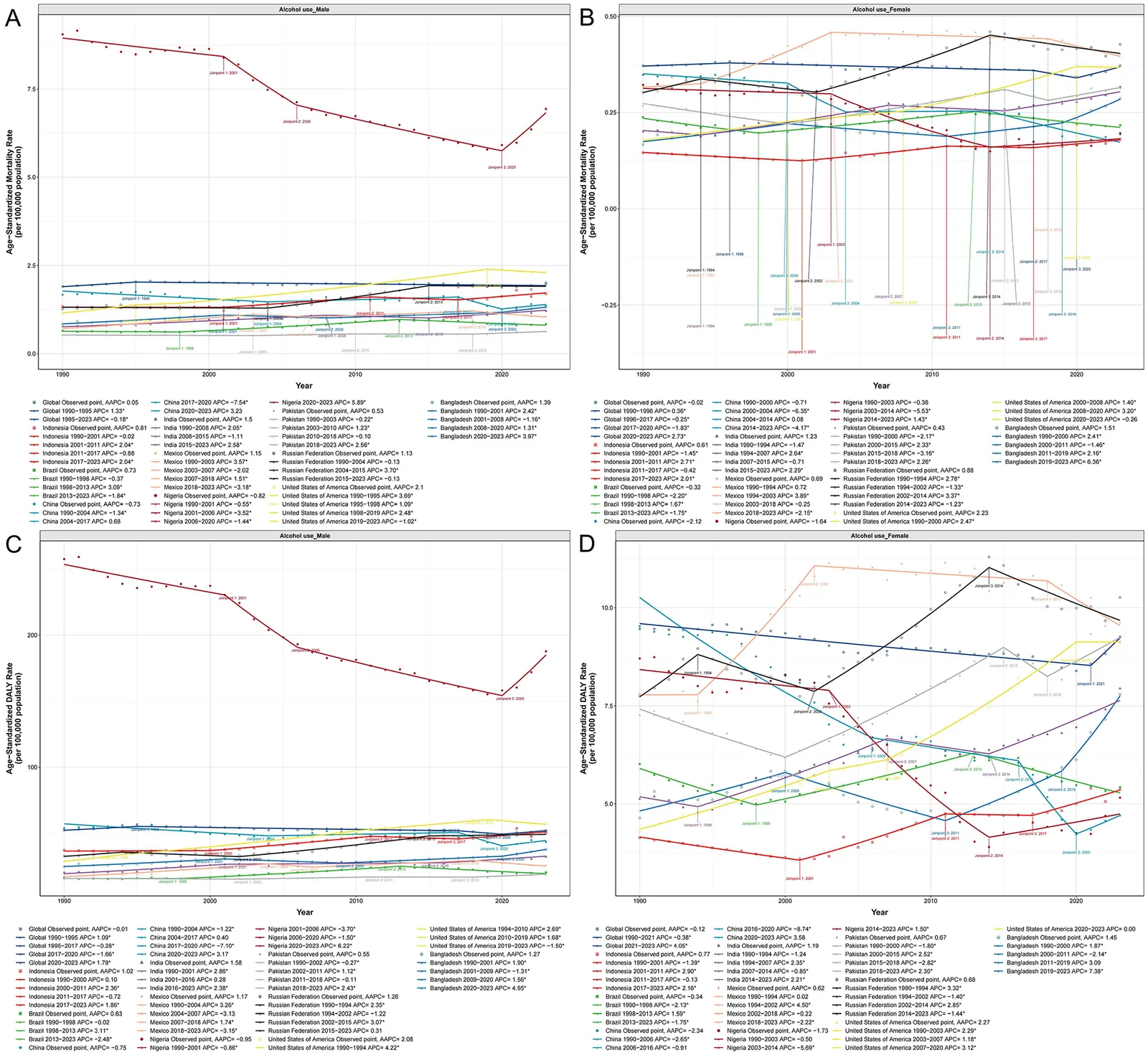
Male/female age-standardized mortality rates and age-standardized DALYS trends for alcohol use. All indices are per 100,000 population. ASMR, Age Standardized Mortality Rate; DALYs, Disability Adjusted Life Years; AAPC, Average Annual Percentage Change; APC, Annual Percentage Change.

### 4.3 Tobacco

Tobacco-attributable ASMR declined globally by 24% between 1990 and 2023 [0.75 [95% UI 0.24–1.25] to 0.57 [0.19–1.01] per 100,000; AAPC −0.77% [95% CI −0.89 to −0.66]] (Supplementary Figure S5), representing one of the few clear public health successes observed (Figure 3). China exemplified this trend: despite exhibiting the highest tobacco-attributable ASMR in 1990 (nearly double the global average), sustained tobacco control policies achieved consistent reductions [AAPC −1.52% [95% CI −1.70 to −1.33], 1990–2023]. However, five countries—India, Indonesia, Nigeria, Pakistan, and Bangladesh—defied the global downward trend, with rising male ASMR in recent years potentially reflecting inadequate tobacco control infrastructure and high secondhand smoke exposure. Among females, concerning increases emerged in seven countries (China, Indonesia, Nigeria, Pakistan, Russia, the United States, and Bangladesh), with Nigeria recording the steepest rise [APC 9.20% [95% CI 0.60–14.13], 2021–2023]. Critically, after continuous decline since 1996, global tobacco-attributable DALYs reversed course in 2020 (Supplementary Figure S6), with males demonstrating significant increases [APC 1.81% [95% CI 0.24–4.55], 2020–2023] and females showing a plateau rather than continued decline. This 2020 inflection point coincided with COVID-19 pandemic disruptions to cessation services and potential stress-related smoking relapse.

**Figure 3:**
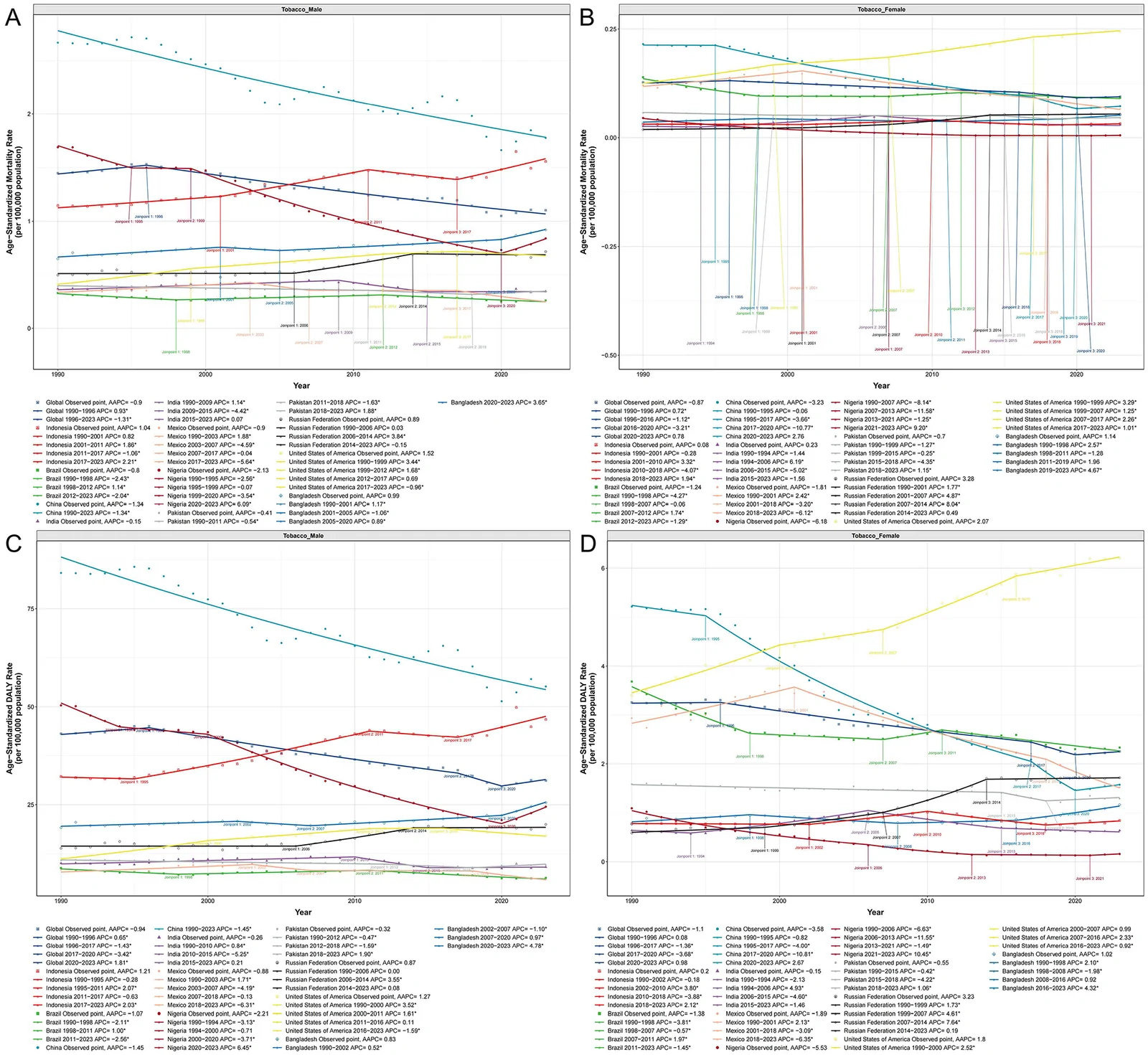
Male/female age-standardized mortality rates and age-standardized DALYS trends for tobacco use. All indices are per 100,000 population. ASMR, Age Standardized Mortality Rate; DALYs, Disability Adjusted Life Years; AAPC, Average Annual Percentage Change; APC, Annual Percentage Change.

### 4.4 Drug use

Drug use-attributable liver cancer burden increased dramatically, with global ASMR rising 79% [0.40 [95% UI 0.28–0.59] to 0.73 [0.56–0.92] per 100,000; AAPC 1.78% [95% CI 1.68–1.87]] (Supplementary Figure S7) and DALYs increasing 51% [11.76 [8.40–16.68] to 17.78 [14.04–22.61] per 100,000; AAPC 1.26% [1.17–1.34]] (Supplementary Figure S8) over the study period, with no evidence of plateauing (Figure 4). Post-2020 acceleration was evident [APC 2.88% [95% CI 1.66–5.08], 2020–2023 for DALYs], marking drug use as the fastest-growing behavioral risk factor. The United States exhibited alarming trends: by 2023, drug use-attributable ASMR reached 2-fold (males) and 3-fold (females) the global average, compared to near-parity in 1990, paralleling the well-documented U.S. opioid epidemic and associated hepatitis C transmission among people who inject drugs. Among males, all countries except Mexico showed increasing ASMR in recent years [Mexico APC −2.75% [95% CI −4.95 to −1.17], 2018–2023], while China showed no net change in male drug use-attributable DALYs across the entire study period [APC −0.14% [95% CI −0.37 to 0.10], 1990–2023]—the only country to avoid escalation. Among females, drug use-attributable ASMR increased in all countries except Mexico, with several demonstrating post-2020 acceleration. Notably, by 2023, drug use-attributable DALYs exceeded tobacco-attributable DALYs in six countries (India, the United States, Pakistan, Brazil, Russia, and Mexico), signaling a fundamental shift in behavioral risk hierarchies.

**Figure 4:**
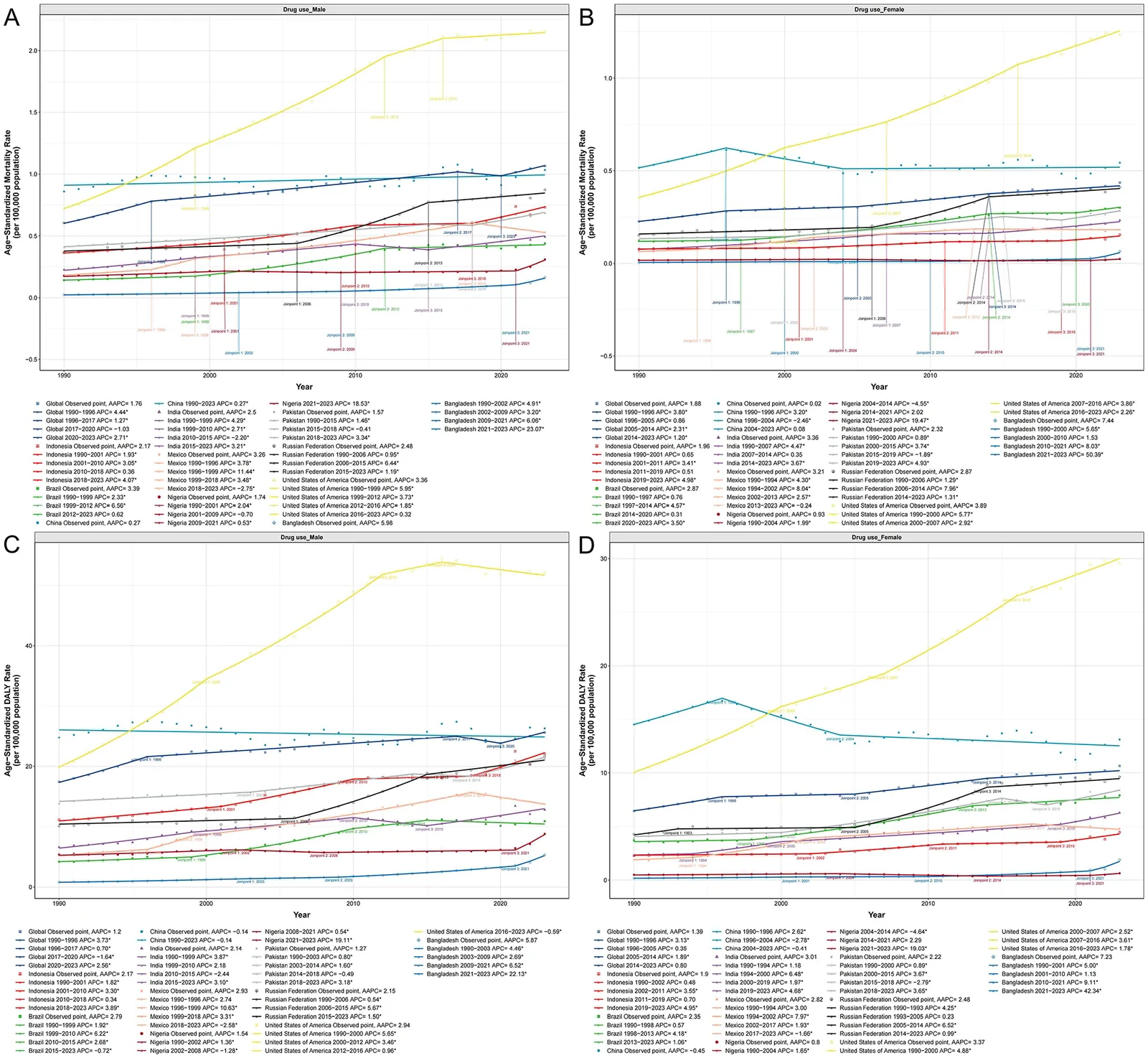
Male/female age-standardized mortality rates and age-standardized DALYS trends for drug use. All indices are per 100,000 population. ASMR, Age Standardized Mortality Rate; DALYs, Disability Adjusted Life Years; AAPC, Average Annual Percentage Change; APC, Annual Percentage Change.

### 4.5 Metabolic risks: the emerging dominant driver

Metabolic risks-attributable liver cancer burden increased 71% globally [ASMR 0.42 [95% UI 0.24–0.64] to 0.73 [0.40–1.05] per 100,000; AAPC 1.67% [95% CI 1.60–1.73], 1990–2023] (Supplementary Figure S9), representing the most consistent upward trend across all risk factors examined (Figure 5). Acceleration since 2020 was particularly pronounced [male APC 3.74% [95% CI 2.88–5.37] in 2020–2023 vs. 1.50% [1.44–1.59] in 1997–2017; female APC 5.17% [2.95–6.26] in 2021–2023 vs. 0.85% [0.75–0.91] in 1996–2021]. DALYs increased 67% [11.52 [95% UI 6.49–17.72] to 19.29 [10.54–28.49] per 100,000] (Supplementary Figure S10), narrowing the gap with alcohol-attributable burden from 2.6-fold to 1.6-fold over the study period. Among women, metabolic risk-attributable DALYs exceeded alcohol-attributable DALYs in eight of 10 countries by 2023, although for both sexes combined alcohol-attributable DALYs remained higher in every country studied. Geographic disparities were striking: Nigeria exhibited male DALYs nearly 4-fold the global average by 2023 [95.17 [95% UI 34.23–204.38] vs. 25.13 [13.61–38.01] per 100,000] with explosive recent growth [APC 11.43% [95% CI 9.01–13.20], 2021–2023], while Bangladesh similarly demonstrated rapid escalation [male APC 8.31% [95% CI 6.42–10.87], 2020–2023]. Among both sexes, only Mexico showed declining ASMR [males APC −1.79% [95% CI −3.53 to −0.57]; females APC −2.08% [−3.94 to −1.01], 2017–2023], whereas China demonstrated non-significant female declines since 2004 [APC −0.48% [95% CI −0.85 to 0.34]]. All other countries experienced increases, with most showing post-2020 acceleration. Female DALYs increased in seven of 10 countries (all except China, Russia, and Mexico), with several exceeding 7% annual growth post-2020.

**Figure 5:**
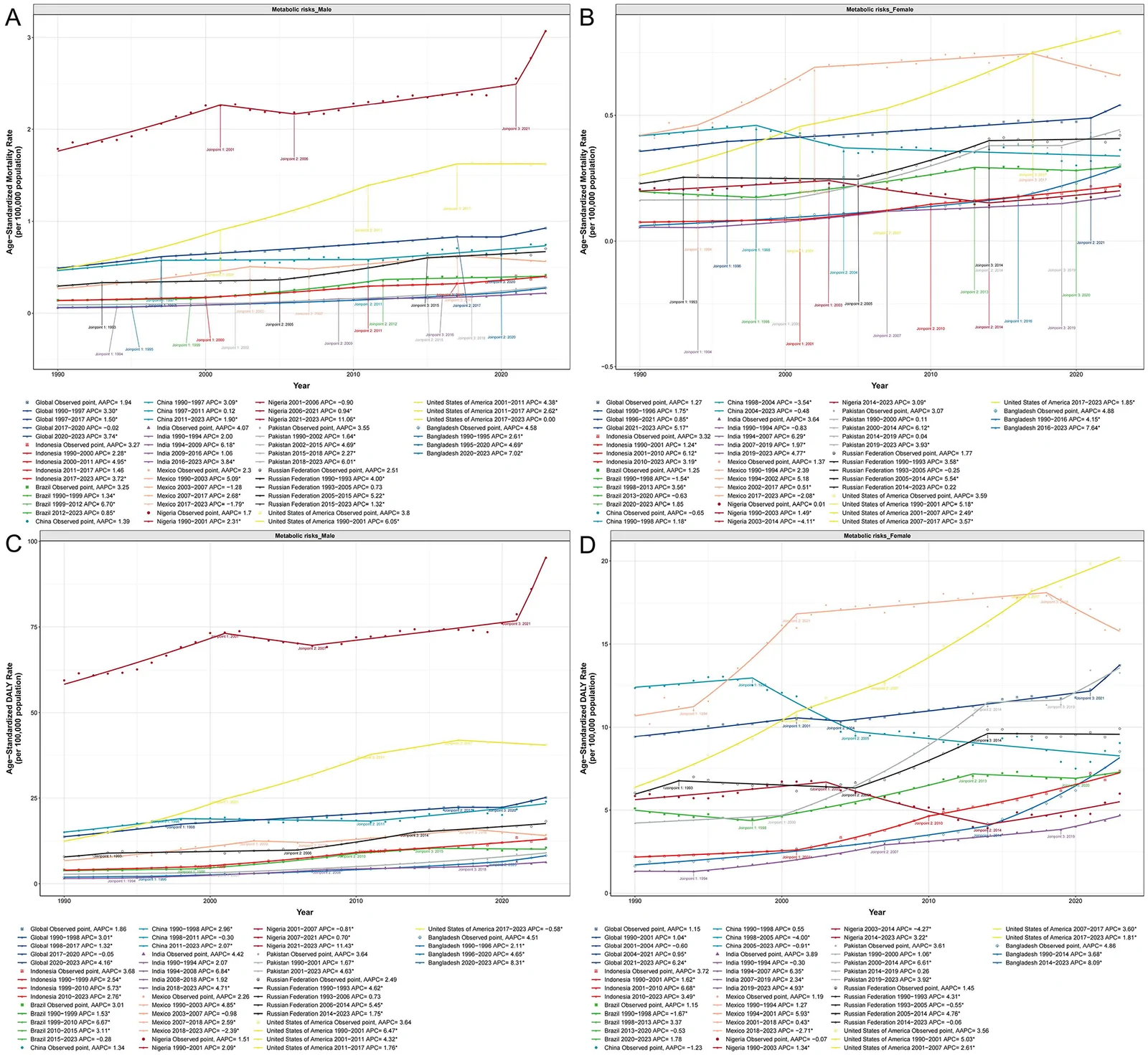
Male/female age-standardized mortality rates and age-standardized DALYS trends for metabolic risks. All indices are per 100,000 population. ASMR, Age Standardized Mortality Rate; DALYs, Disability Adjusted Life Years; AAPC, Average Annual Percentage Change; APC, Annual Percentage Change.

### 4.6 High body mass index

High BMI-attributable burden demonstrated the steepest escalation among all risk factors, with global ASMR increasing 84% [0.32 [95% UI 0.14–0.53] to 0.59 [0.28–0.93] per 100,000; AAPC 1.86% [95% CI 1.81–1.91]] (Supplementary Figure S11) and DALYs nearly doubling [8.96 [3.90–14.82] to 16.26 [7.50–25.62] per 100,000; 81% increase] (Supplementary Figure S12) between 1990 and 2023 (Figure 6). Post-2020 acceleration was evident across both sexes [male APC 3.85% [95% CI 3.01–5.43] in 2020–2023 vs. 1.61% [1.54–1.70] in 1998–2017; female APC 5.57% [3.72–6.47] in 2021–2023 vs. 1.06% [0.99–1.11] in 1997–2021] (Supplementary Figures S11, S12). By 2023, Nigeria recorded the highest male ASMR [2.77 [95% UI 0.95–6.12] per 100,000] with explosive recent growth [APC 11.14% [95% CI 7.96–12.87], 2021–2023], while the United States exhibited the highest female ASMR [0.67 [95% UI 0.33–1.06] per 100,000], reflecting obesity prevalence exceeding 40%. Mexico represented the sole country demonstrating declining male ASMR [APC −2.29% [95% CI −4.52 to −0.78], 2018–2023] and female DALYs, potentially reflecting national obesity prevention initiatives. China displayed a paradoxical pattern: stable female ASMR since 2004 but sharply rising DALYs post-2021 [APC 7.91% [95% CI 1.62–11.54]], possibly indicating improved survival with obesity-related complications. Among females, nine of 10 countries (all except Mexico) demonstrated rising ASMR, with Bangladesh and China recording the steepest DALY increases (both >7% annually). Notably, by 2023, high BMI-attributable burden exceeded tobacco-attributable burden in six of 10 countries (the United States, Pakistan, Nigeria, Brazil, Russia, and Mexico), marking a fundamental epidemiological transition from traditional to metabolic risk dominance.

**Figure 6:**
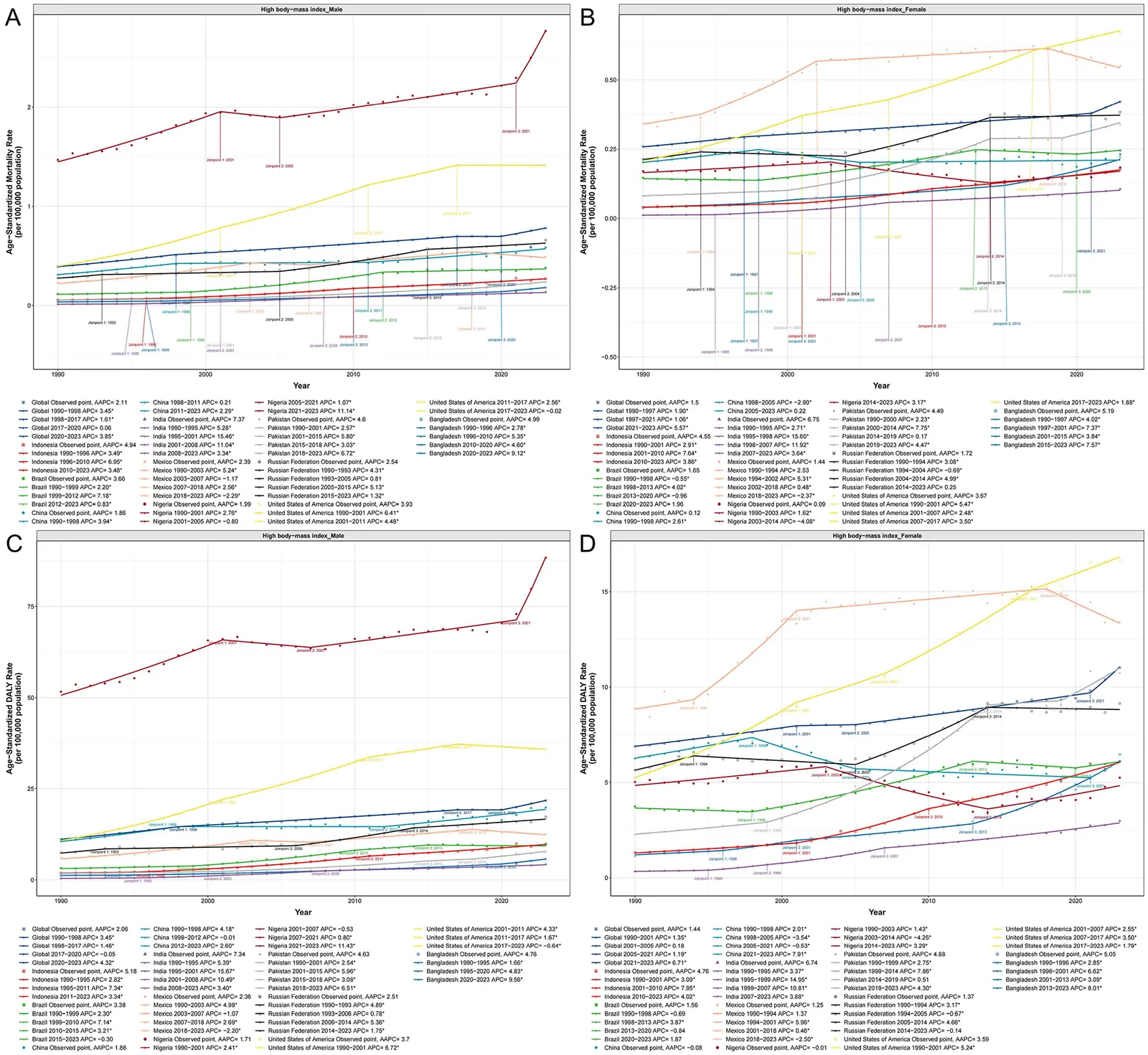
Male/female age-standardized mortality rates and age-standardized DALYS trends for high body mass index. All indices are per 100,000 population. ASMR, Age Standardized Mortality Rate; DALYs, Disability Adjusted Life Years; AAPC, Average Annual Percentage Change; APC, Annual Percentage Change.

### 4.7 High fasting plasma glucose

High fasting glucose-attributable burden increased 41% for ASMR [0.11 [95% UI 0.07–0.16] to 0.15 [0.11–0.20] per 100,000] (Supplementary Figure S13) and 29% for DALYs [2.73 [1.87–4.05] to 3.53 [2.67–4.80] per 100,000] (Supplementary Figure S14) globally, closely paralleling diabetes epidemic trajectories (Figure 7). In absolute terms these are the smallest changes of any exposure examined—approximately 0.04 deaths and 0.8 DALYs per 100,000 over 34 years—and the uncertainty intervals for the 1990 and 2023 estimates overlap substantially, so the relative increases should be read with that in mind. Male ASMRs demonstrated biphasic increase patterns [APC 1.97% [95% CI 1.66–2.54], 1990–1999; plateau 1999–2004; APC 1.29% [1.17–1.50], 2004–2023], whereas females showed relatively stable trends until sharp post-2021 acceleration [APC 4.01% [95% CI 0.50–6.12], 2021–2023]. Among males, all countries except Mexico [APC −2.39% [95% CI −3.89 to −1.30], 2017–2023] demonstrated recent ASMR increases, although the United States plateaued after 2017 [APC 0.14% [−0.39 to 0.59], *p* = 0.52]. Nigeria exhibited the steepest DALY escalation [APC 7.60% [95% CI 5.57–11.47], 2020–2023]. Among females, only China demonstrated sustained ASMR declines since 1990, with Mexico showing recent decreases; all other countries exhibited increases. The United States recorded the largest overall female DALY increase [1.34 [95% UI 1.00–1.81] to 4.52 [3.60–6.15] per 100,000; AAPC 3.77% [95% CI 3.71–3.84], 1990–2023], despite advanced diabetes care infrastructure, potentially reflecting suboptimal glycemic control in chronic liver disease populations or diagnostic delays in women.

**Figure 7:**
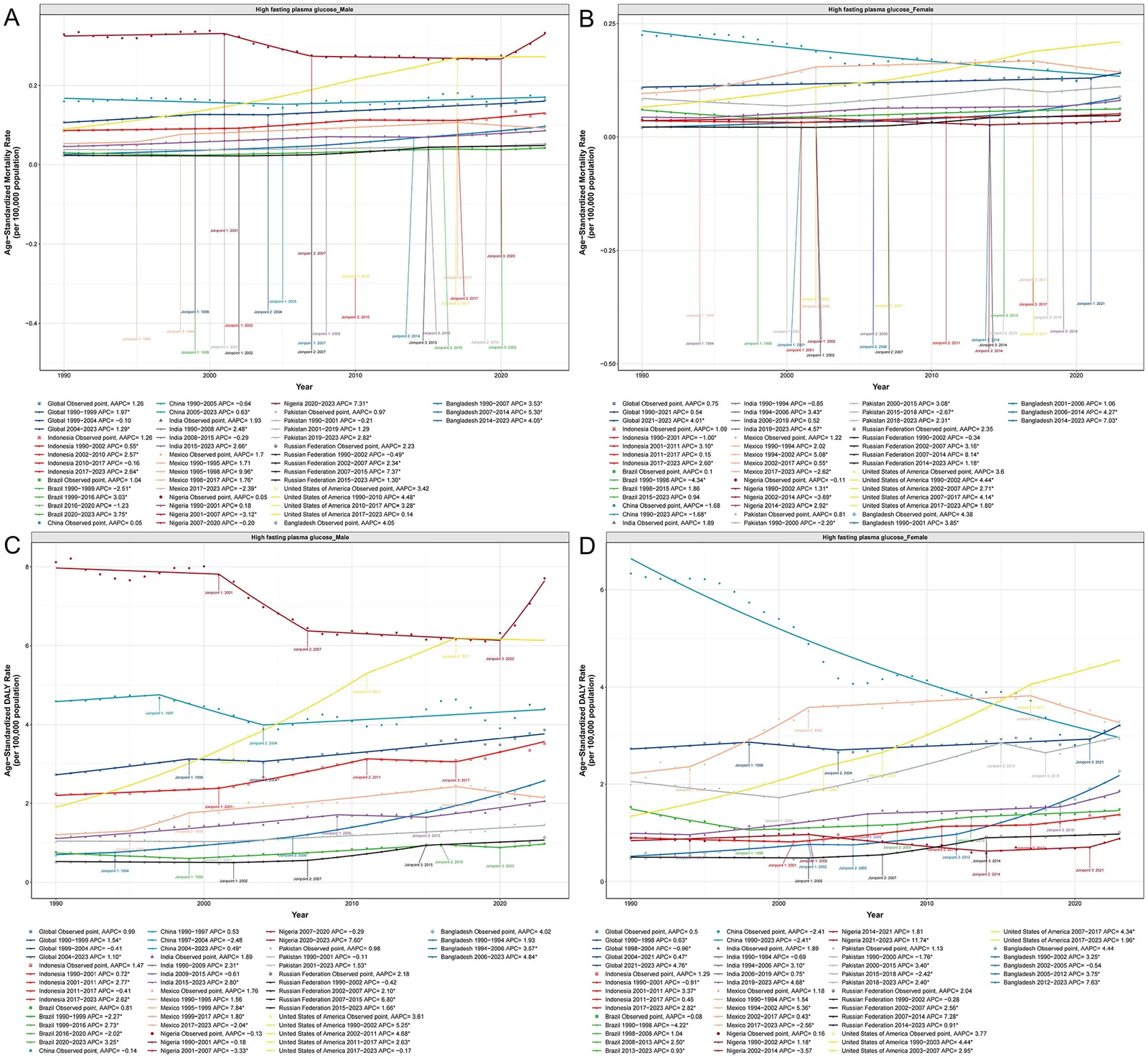
Male/female age-standardized mortality rates and age-standardized DALYS trends for high fasting plasma glucose. All indices are per 100,000 population. ASMR, Age Standardized Mortality Rate; DALYs, Disability Adjusted Life Years; AAPC, Average Annual Percentage Change; APC, Annual Percentage Change.

### 4.8 Critical temporal inflection point: the 2020 watershed

Joinpoint regression identified synchronized trend accelerations across multiple risk factors during 2020–2021 (Supplementary Tables S1, S2). Metabolic risks, high BMI, and drug use all demonstrated significant post-2020 increases compared to pre-pandemic trends (*p* < 0.05 for all; these *p*-values are descriptive, for the reasons set out in the Statistical analysis section), while tobacco-attributable burden reversed from declining to increasing. This synchronized pattern across diverse risk factors suggests common pandemic-associated mechanisms: healthcare access disruptions, lifestyle deterioration (reduced physical activity, unhealthy dietary changes), stress-related substance use increases, and delayed cancer diagnosis with potential stage migration.

### 4.9 Sex-specific patterns

Women demonstrated paradoxically rising burdens despite lower absolute rates across most risk factors. Globally, female behavioral risk-related ASMR rose faster than male over the full period [AAPC 0.66% [95% CI 0.51–0.78] vs. 0.10% [0.03–0.19], *p* = 0.008]. Male rates rose to 1996 and declined modestly thereafter [APC −0.22% [−0.30 to −0.15], 1996–2023], whereas female rates were flat between 1995 and 2021 before rising sharply [APC 2.98% [0.42–4.37], 2021–2023]. Metabolic risks-related ASMR increased among females in all countries except Mexico and China, with the most recent female trend exceeding the corresponding male trend in four countries (India, the United States, Brazil, and Bangladesh). By 2023, U.S. women exhibited behavioral risks-related DALYs exceeding twice the global average, while high BMI-related female DALYs increased in eight of 10 countries. These patterns likely reflect increasing female substance use in rapidly developing countries, higher obesity prevalence among women, greater metabolic syndrome prevalence despite lower diabetes rates, and gender-specific healthcare access barriers.

### 4.10 Country-specific risk hierarchies and population impact

By 2023, the relative contribution of risk factors varied substantially. Traditional alcohol-dominant patterns persisted in China and Nigeria (alcohol >3 × other risks), transitional mixed patterns emerged in India, Indonesia, Pakistan, Bangladesh, and Brazil (converging alcohol and metabolic contributions), while metabolic-dominant patterns characterized the United States and Mexico (metabolic risks approaching or exceeding alcohol). Notably, the United States uniquely exhibited drug use burden exceeding tobacco. In 2023 the 10 most populous countries accounted for 258,072 (51%) of the 507,628 liver cancer deaths estimated globally. Behavioral risks accounted for 203,332 of the global deaths [population attributable fraction 40.1% [95% UI 33.7–46.5], up from 33.3% [26.0–40.5] in 1990] and metabolic risks for 66,483 [13.1% [7.5–19.0], up from 6.9% [3.9–9.8]]; the corresponding ten-country totals were 105,512 (52% of the global behavioral total) and 29,094 (44% of the global metabolic total). Because GBD estimates attributable burden jointly and the behavioral and metabolic categories overlap, these fractions cannot be added together into a single figure for all modifiable risks.

## 5 Discussion

This comprehensive 34-year analysis of liver cancer burden across the world's 10 most populous countries, representing 60% of global population, reveals a critical epidemiological transition: while traditional behavioral risks from tobacco and alcohol stabilize or decline in many regions, metabolic dysfunction and drug use now drive accelerating burden increases. Three principal findings demand urgent attention: first, metabolic risk-attributable liver cancer mortality increased 71%, narrowing the gap with alcohol as the leading driver by 2023; second, drug use-attributable mortality rose by 79%, with the United States exhibiting rates 2–3-fold above global averages; third, a synchronized inflection point in 2020–2021 across multiple risk factors coincides with the pandemic period and is compatible with, though does not by itself demonstrate, pandemic-associated lifestyle disruptions compounding pre-existing adverse trends. These findings necessitate fundamental reorientation of liver cancer prevention from viral hepatitis-centric approaches toward integrated metabolic disease and substance use disorder management.

Our finding that metabolic risks-attributable DALYs [19.29 [95% UI 10.54–28.49] per 100,000 in 2023] now approach alcohol-attributable DALYs [29.96 [95% UI 22.43–39.68] per 100,000]—narrowing from a 2.6-fold gap in 1990 to 1.6-fold by 2023—marks a watershed in liver cancer epidemiology. This transition parallels but substantially exceeds the pace of global obesity and diabetes increases: while worldwide adult obesity prevalence more than doubled between 1990 and 2021, from 5.8% to 14.8% in men and from 10.2% to 20.8% in women (8), our data demonstrate high BMI-attributable liver cancer burden increased 81%, suggesting non-linear dose-response relationships or synergistic effects with concurrent metabolic derangements. The near-doubling of high BMI-related DALYs represents the steepest escalation among all risk factors examined, validating emerging concerns that metabolic dysfunction-associated steatotic liver disease (MASLD)—whose global prevalence rose from 25.3% to 38.0% of adults (9)—will become the predominant etiology of hepatocellular carcinoma in high-income and middle-income countries within the next decade (10–12).

Critically, our identification of post-2020 acceleration in metabolic risk burden [male APC increasing from 1.50% [95% CI 1.44–1.59] in 1997–2017 to 3.74% [2.88–5.37] in 2020–2023; female APC from 0.85% [0.75–0.91] in 1996–2021 to 5.17% [2.95–6.26] in 2021–2023] is consistent with the COVID-19 pandemic having catalyzed adverse lifestyle changes that persist beyond acute pandemic phases. Population-based studies document pandemic-associated weight gain during lockdowns in both adults and children (13–16), reduced physical activity (17, 18), and worsening glycemic control among people with diabetes (19, 20); disruptions in healthcare services, constraints on healthcare resources, and delays in diagnosis associated with the COVID-19 pandemic may also need to be considered (21, 22). Measurable increases in cancer burden followed within 2–3 years in our data, although an ecological analysis of this kind cannot establish that these exposures caused the increase. The rapidity of this effect—visible in age-standardized mortality data despite liver cancer's typical latency—likely reflects both accelerated progression in pre-existing chronic liver disease and earlier age of MASLD-related hepatocellular carcinoma onset, consistent with recent reports of increasing incidence among individuals aged 25–44 years (10–12).

Geographic heterogeneity in metabolic risk burden underscores distinct epidemic stages and health system capacities. Nigeria's 4-fold higher male DALYs vs. global averages, with explosive 11.43% [95% CI 9.01–13.20] annual growth post-2021, likely reflects intersecting epidemics of rapid urbanization, dietary westernization, undiagnosed diabetes (an estimated 53.6% of adults living with diabetes in the Africa region remain undiagnosed, the highest proportion of any International Diabetes Federation region) (23, 24), and minimal MASLD surveillance infrastructure. Conversely, Mexico's declining metabolic risk trends—unique among studied countries—temporally coincide with implementation of comprehensive obesity prevention policies including sugar-sweetened beverage taxation (2014), front-of-package warning labels (2020), and school food environment regulations (25), providing proof-of-concept that population-level interventions can reverse adverse trajectories even amid global pandemic disruptions.

The 79% increase in drug use-attributable ASMRs, with post-2020 acceleration and surpassing of tobacco-attributable burden in six countries, represents a fundamentally under-recognized driver of liver cancer mortality. GBD attributes this burden to drug use as an exposure and does not partition it by downstream etiology, so our data cannot identify the mechanism. One plausible pathway—supported by modeling studies external to this analysis rather than by anything estimated here—is hepatitis C transmission among people who inject drugs, with liver disease progression potentially accelerated by direct drug hepatotoxicity, concomitant alcohol use, and barriers to antiviral therapy access (26, 27). We offer this as a hypothesis consistent with our findings rather than a conclusion drawn from them; separating it from other contributions to drug use-attributable liver cancer would require etiology-specific data that the GBD risk-factor estimates do not provide. The United States exemplifies this crisis, with our finding of 3-fold higher female drug use-attributable ASMRs paralleling documented increases in opioid use and injection drug use among women (28), driven by prescription opioid misuse transitioning to heroin and fentanyl use (29). Critically, while direct-acting antivirals achieve high cure rates, coverage of HCV testing and treatment among people who inject drugs remains low and highly uneven between countries (30–32), creating a widening gap between therapeutic capacity and implementation that our mortality data starkly illustrate.

The contrast between declining drug use burden in Mexico [male ASMR APC −2.75% [95% CI −4.95 to −1.17], 2018–2023; female DALY APC −1.66% [−3.74 to −0.42], 2017–2023] vs. escalating trends in most other countries highlights the potential impact of harm reduction approaches. Mexico's 2009–2019 expansion of opioid agonist therapy, needle-syringe programs, and integrated HCV testing/treatment within addiction services (33) demonstrates that evidence-based interventions can reverse drug use-attributable liver cancer mortality even amid ongoing substance use epidemics. Conversely, persistently high burden in countries with punitive drug policies underscores that criminalization-focused approaches fail to mitigate health consequences (32, 34).

The 24% global decline in tobacco-attributable liver cancer ASMRs represents one of few unambiguous public health successes, with China's sustained reduction [AAPC −1.52% [95% CI −1.70 to −1.33]] demonstrating that even countries with high baseline burden and substantial tobacco industry presence can achieve meaningful progress through comprehensive tobacco control. However, our identification of a 2020 inflection point—with global male DALYs reversing from declining (pre-2020) to increasing [APC 1.81% [95% CI 0.24–4.55], 2020–2023]—threatens to erode decades of gains. This reversal likely reflects pandemic-associated disruptions to cessation services (clinic closures, reduced healthcare access), stress-related relapse among former smokers, and increased initiation among adolescents during lockdowns (35–37). The concerning increases in female tobacco-attributable ASMRs across seven countries, particularly Nigeria's 9.20% [95% CI 0.60–14.13] annual growth post-2021, reflect targeted tobacco industry marketing toward women in low-regulation environments and rising social acceptability of female smoking during economic transitions (38–41).

Our finding that women demonstrated rising burdens across most risk factors despite lower absolute rates reveals systematic gender disparities in both exposure patterns and healthcare access. The faster global increase in female behavioral risk ASMRs [AAPC 0.66% [95% CI 0.51–0.78]] than male [AAPC 0.10% [0.03–0.19]] reflects documented narrowing of gender gaps in substance use globally, with particularly rapid increases in alcohol consumption among women in upper-middle-income countries (42, 43). Additionally, higher obesity prevalence among women in most countries (20.8% vs. 14.8% among men globally in 2021) (8) compounds metabolic risk exposure, while evidence suggests women experience faster MASLD progression to fibrosis and hepatocellular carcinoma at lower BMI thresholds than men (10–12).

Beyond differential exposure, gender-specific healthcare barriers likely contribute to observed burden increases. Studies document longer diagnostic delays for liver cancer among women (44). The 2–3-fold higher U.S. female rates vs. global averages, despite universal healthcare access for older adults through Medicare, suggests these disparities persist even in well-resourced settings, mandating sex-specific intervention design and implementation research.

Our findings extend and contextualize previous GBD-based analyses that examined individual risk factors in isolation. A recent study of metabolic risk-attributable liver cancer (1990–2021) reported consistent increases in case numbers (1), while another documented declining tobacco and rising alcohol burdens over the same period (2). Our analysis advances understanding in four key dimensions: first, examination of seven behavioral and metabolic risk factors within one framework enables direct comparison of relative contributions and identification of epidemiological transitions from alcohol-dominant to metabolic-dominant patterns; second, extension through 2023 captures critical pandemic-era changes, with inflection point analysis quantifying acceleration magnitude; third, sex-stratified analysis across demographically diverse nations reveals systematic gender disparities obscured in aggregate data; fourth, focus on the world's 10 most populous countries enables actionable country-specific comparisons while maintaining global population representation.

Our metabolic risk findings align with prospective cohort data demonstrating diabetes increases hepatocellular carcinoma risk, obesity and metabolic syndrome (2–5, 8, 10–12). However, our documentation of near-doubling DALYs substantially exceeds predicted increases from prevalence changes alone, suggesting either: (1) synergistic effects when multiple metabolic derangements coexist; (2) non-linear dose-response relationships at higher BMI/glucose levels now prevalent in populations; or (3) cohort effects, with earlier-life metabolic exposures conferring greater carcinogenic risk than late-life exposures captured in previous cohort studies enrolling middle-aged adults.

Regarding drug use, our findings complement that the increase in U.S. drug use-attributable burden parallels documented increases in hepatocellular carcinoma incidence validating that upstream injection drug use increases rapidly translate into downstream cancer burden.

The biological mechanisms linking metabolic dysfunction to hepatocellular carcinoma involve interconnected pathways: insulin resistance promotes hepatic lipid accumulation and lipotoxicity, triggering oxidative stress, inflammation, and hepatocyte injury (45). Chronic inflammation induces fibrogenesis and cirrhosis development, the principal hepatocellular carcinoma risk substrate. Critically, emerging evidence demonstrates MASLD-related hepatocellular carcinoma increasingly develops in non-cirrhotic livers (3–5, 10–12), challenging traditional surveillance paradigms focused on cirrhosis screening. This mechanism explains both the rapidity of burden increases we observed (shorter latency than cirrhosis-mediated pathways) and the younger age distribution of metabolic risk-attributable cases.

This study's strengths include: examination of seven behavioral and metabolic risk factors within a single framework, enabling the epidemiological transition to be assessed across exposures; extended 34-year follow-up with joinpoint analysis detecting critical inflection points; large population coverage (about 60% of the world's population, though not geographic representativeness); sex-stratified analysis revealing systematic gender disparities; and focus on demographically diverse nations spanning income levels, enabling cross-context comparison while avoiding small-country instability.

However, important limitations warrant consideration. First, GBD methodology relies on pooled risk-outcome relationships that may not reflect country-specific dose-response patterns or effect modification by genetic, dietary, or environmental factors. For instance, Asian populations demonstrate hepatocellular carcinoma risk at lower BMI thresholds than Western populations, potentially underestimating metabolic risk burden in Asian countries. Second, liver cancer diagnostic capacity varies substantially across settings: even where surveillance is available its sensitivity for early-stage disease is limited—ultrasound alone detects 47% of early-stage hepatocellular carcinomas and ultrasound with alpha-fetoprotein 63%(46)—and resource-limited settings rely predominantly on clinical diagnosis, so ascertainment is likely to be lower still. This heterogeneity likely underestimates true burden in countries like Nigeria and Bangladesh while more accurately capturing burden in the United States and China. Sensitivity analyses using alternative diagnostic assumptions would strengthen inference but were beyond this study's scope.

Third, risk factor attribution in individuals with multiple exposures (e.g., concurrent obesity, diabetes, and alcohol use) involves modeling assumptions. GBD employs counterfactual methodology estimating burden reduction if specific risks were eliminated, which may overestimate independent effects when risks act synergistically. Our finding that the population attributable fraction rose from 33% to 40% for behavioral risks and from 7% to 13% for metabolic risks suggests either: (1) actual increases in risk factor prevalence and potency; or (2) improved attribution modeling in recent GBD iterations. However, consistency with prospective cohort data and biological plausibility supports the former interpretation.

Fourth, our 2020–2023 findings reflect only 3–4 years post-pandemic follow-up. Liver cancer typically develops after decades of chronic liver disease, raising questions about how such rapid burden increases manifest. Plausible explanations include: (1) accelerated progression in pre-existing advanced fibrosis/cirrhosis populations, where metabolic decompensation or substance use relapse could precipitate hepatocellular carcinoma within 1–3 years; (2) diagnostic delays during pandemic-related healthcare disruptions leading to later-stage diagnosis captured as higher mortality; (3) incidental surveillance interruptions in known chronic liver disease patients resulting in delayed tumor detection; or (4) population-level compositional changes (e.g., pandemic-associated excess mortality among healthier individuals, enriching remaining populations for chronic disease). Longer follow-up and incidence data (not available in current GBD iterations) will clarify whether 2020–2023 trends represent sustained shifts vs. temporary perturbations.

Fifth, our focus on the 10 most populous countries maximizes population coverage but not geographic representativeness: Europe other than Russia, the entire Middle East, and all of Africa other than Nigeria are absent, as are all small-to-medium nations, so the aggregate patterns reported here should not be read as global averages. For instance, Pacific Island nations experience extreme obesity prevalence (up to 60% in some populations) but were excluded from our analysis. Regional GBD analyses could address this limitation. Sixth, drug use-attributable burden is estimated for drug use as an exposure and cannot be decomposed into its downstream etiologies, so the contribution of hepatitis C transmission relative to other pathways cannot be quantified from these data. Seventh, because a large number of joinpoint models were fitted, individual inflection points should be read as descriptive rather than confirmatory, as set out in the Statistical analysis section; our interpretation rests on shifts replicated across both outcomes, both sexes, and multiple countries. Finally, ecological study design precludes individual-level inference: observed population-level associations between risk factor trends and cancer burden may not reflect individual-level causation due to confounding, lag effects, and cohort compositional changes.

This comprehensive 34-year analysis spanning 60% of global population documents a critical epidemiological transition in liver cancer: metabolic dysfunction and substance use now drive burden increases, with metabolic risk-attributable mortality rivaling alcohol as the leading driver by 2023. The post-2020 acceleration across multiple risk factors—metabolic risks, high BMI, and drug use—coincides with the pandemic period and is consistent with, though does not demonstrate, pandemic-associated lifestyle changes compounding pre-existing adverse trends, while tobacco-attributable burden reversed from decades of decline. These findings mandate urgent reorientation of liver cancer prevention from viral hepatitis-centric approaches toward integrated strategies addressing obesity, diabetes, and substance use disorders.

## Data Availability

The original contributions presented in the study are included in the article/Supplementary material, further inquiries can be directed to the corresponding authors.

## References

[1] Chan SL Sun HC Xu Y Zeng H El-Serag HB Lee JM . The Lancet Commission on addressing the global hepatocellular carcinoma burden: comprehensive strategies from prevention to treatment. Lancet. (2025) 406:731–78. doi: 10.1016/S0140-6736(25)01042-6 40744051

[2] Marengo A Rosso C Bugianesi E. Liver cancer: connections with obesity, fatty liver, and cirrhosis. Annu Rev Med. (2016) 67:103–17. doi: 10.1146/annurev-med-090514-013832 26473416

[3] Hwang SY Danpanichkul P Agopian V Mehta N Parikh ND Abou-Alfa GK . Hepatocellular carcinoma: updates on epidemiology, surveillance, diagnosis and treatment. Clin Mol Hepatol. (2025) 31:S228–54. doi: 10.3350/cmh.2024.0824 39722614 PMC11925437

[4] Brown ZJ Tsilimigras DI Ruff SM Mohseni A Kamel IR Cloyd JM . Management of hepatocellular carcinoma: a review. JAMA Surg. (2023) 158:410–20. doi: 10.1001/jamasurg.2022.7989 36790767

[5] Tan EY Danpanichkul P Yong JN Yu Z Tan DJH Lim WH . Liver cancer in 2021: global burden of disease study. J Hepatol. (2025) 82:851–60. doi: 10.1016/j.jhep.2024.10.031 39481652

[6] Wu C Targher G Byrne CD Mao Y Cheung TT Yilmaz Y . Global, regional, and national burden of primary liver cancer attributable to metabolic risks: an analysis of the global burden of disease study 1990-2021. Am J Gastroenterol. (2025) 120:2280–90. doi: 10.14309/ajg.0000000000003288 39749919

[7] Liu C Shi T Li S Wu S Chen J Cai C . Global, regional, and national liver cancer attributable to smoking and alcohol use burden, 1990-2021: analysis for the global burden of disease 2021 study. BMC Public Health. (2025) 25:2037. doi: 10.1186/s12889-025-23184-3 40457284 PMC12128314

[8] GBD 2021 Adult BMI Collaborators. Global, regional, and national prevalence of adult overweight and obesity, 1990-2021, with forecasts to 2050: a forecasting study for the Global Burden of Disease Study 2021. Lancet. (2025) 405:813–38. doi: 10.1016/S0140-6736(25)00355-1 40049186 PMC11920007

[9] Younossi ZM Golabi P Paik JM Henry A Van Dongen C Henry L. The global epidemiology of nonalcoholic fatty liver disease (NAFLD) and nonalcoholic steatohepatitis (NASH): a systematic review. Hepatology. (2023) 77:1335–47. doi: 10.1097/HEP.0000000000000004 36626630 PMC10026948

[10] Zhang H Zhou XD Shapiro MD Lip GYH Tilg H Valenti L . Global burden of metabolic diseases, 1990-2021. Metabolism. (2024) 160:155999. doi: 10.1016/j.metabol.2024.155999 39151887

[11] Xiao J Wang F Yuan Y Gao J Xiao L Yan C . Epidemiology of liver diseases: global disease burden and forecasted research trends. Sci China Life Sci. (2025) 68:541–57. doi: 10.1007/s11427-024-2722-2 39425834

[12] Zhao Y Bo Y Zu J Xing Z Yang Z Zhang Y. Global burden of chronic liver disease and temporal trends: a population-based analysis from 1990 to (2021) with projections to 2050. Liver Int. (2025) 45:e70155. doi: 10.1111/liv.70155 40421876

[13] Betts SS Adise S Hayati Rezvan P Marshall AT Kan E Johnson DL . Socioeconomic adversity and weight gain during the COVID-19 pandemic. JAMA Pediatr. (2023) 177:1102–5. doi: 10.1001/jamapediatrics.2023.2823 37578771 PMC10425859

[14] Zeigler Z. COVID-19 self-quarantine and weight gain risk factors in adults. Curr Obes Rep. (2021) 10:423–33. doi: 10.1007/s13679-021-00449-7 34251647 PMC8273568

[15] Seal A Schaffner A Phelan S Brunner-Gaydos H Tseng M Keadle S . COVID-19 pandemic and stay-at-home mandates promote weight gain in US adults. Obesity. (2022) 30:240–8. doi: 10.1002/oby.23293 34467670 PMC8662140

[16] Lubrecht J Arayess L Reijnders D Hesselink ML Velde GT Janse A . Weight gain in children during the COVID-19 pandemic and the protective effect of lifestyle intervention in children with obesity. Obes Facts. (2022) 15:600–8. doi: 10.1159/000525298 35640561 PMC9421665

[17] Oliveira MR Sudati IP Konzen VM de Campos AC Wibelinger LM Correa C . COVID-19 and the impact on the physical activity level of elderly people: A systematic review. Exp Gerontol. (2022) 159:111675. doi: 10.1016/j.exger.2021.111675 34954282 PMC8695515

[18] Clemente-Suárez VJ Beltrán-Velasco AI Ramos-Campo DJ Mielgo-Ayuso J Nikolaidis PA Belando N . Physical activity and COVID-19. The basis for an efficient intervention in times of COVID-19 pandemic. Physiol Behav. (2022) 244:113667. doi: 10.1016/j.physbeh.2021.113667 34861297 PMC8632361

[19] Pranata R Henrina J Raffaello WM Lawrensia S Huang I. Diabetes and COVID-19: the past, the present, and the future. Metabolism. (2021) 121:154814. doi: 10.1016/j.metabol.2021.154814 34119537 PMC8192264

[20] Singh AK Khunti K. COVID-19 and diabetes. Annu Rev Med. (2022) 73:129–47. doi: 10.1146/annurev-med-042220-011857 34379444

[21] COVIDSurg Collaborative. Effect of COVID-19 pandemic lockdowns on planned cancer surgery for 15 tumour types in 61 countries: an international, prospective, cohort study. Lancet Oncol. (2021) 22:1507–17. doi: 10.1016/S1470-2045(21)00493-9 34624250 PMC8492020

[22] Shah R Hanna NM Loo CE David M Mafra A Fink H . The global impact of the COVID-19 pandemic on delays and disruptions in cancer care services: a systematic review and meta-analysis. Nat Cancer. (2025) 6:194–204. doi: 10.1038/s43018-024-00880-4 39747650

[23] Ogurtsova K Guariguata L Barengo NC Ruiz PL Sacre JW Karuranga S . IDF diabetes Atlas: global estimates of undiagnosed diabetes in adults for 2021. Diabetes Res Clin Pract. (2022) 183:109118. doi: 10.1016/j.diabres.2021.109118 34883189

[24] GBD 2021 Diabetes Collaborators. Global, regional, and national burden of diabetes from 1990 to 2021 with projections of prevalence to 2050: a systematic analysis for the Global Burden of Disease Study 2021. Lancet. (2023) 402:203–34. doi: 10.1016/S0140-6736(23)01301-6 37356446 PMC10364581

[25] Colchero MA Popkin BM Rivera JA Ng SW. Beverage purchases from stores in Mexico under the excise tax on sugar sweetened beverages: observational study. BMJ. (2016) 352:h6704. doi: 10.1136/bmj.h6704 26738745 PMC4986313

[26] Liang Y Lin X Zhang M Wang S Dong Y Xie Z . Synergistic impact of high body-mass index on HBV/HCV-related liver cancer burden globally: analysis and projections 1990-2050. Public Health. (2025) 247:105893. doi: 10.1016/j.puhe.2025.105893 40743866

[27] Shayan SJ Nazari R Kiwanuka F. Prevalence of HIV and HCV among injecting drug users in three selected WHO-EMRO countries: a meta-analysis. Harm Reduct J. (2021) 18:59. doi: 10.1186/s12954-021-00505-4 34044849 PMC8161998

[28] Goetz TG Becker JB Mazure CM. Women, opioid use and addiction. FASEB J. (2021) 35:e21303. doi: 10.1096/fj.202002125R 33433026

[29] Wilson N Kariisa M Seth P Smith H Davis NL. Drug and opioid-involved overdose deaths - United States, 2017-2018. MMWR Morb Mortal Wkly Rep. (2020) 69:290–7. doi: 10.15585/mmwr.mm6911a4 32191688 PMC7739981

[30] Lee HW Lee H Kim BK Chang Y Jang JY Kim DY. Cost-effectiveness of chronic hepatitis C screening and treatment. Clin Mol Hepatol. (2022) 28:164–73. doi: 10.3350/cmh.2021.0193 34955002 PMC9013616

[31] Martinello M Hajarizadeh B Grebely J Dore GJ Matthews GV. HCV cure and reinfection among people with HIV/HCV coinfection and people who inject drugs. Curr HIV/AIDS Rep. (2017) 14:110–21. doi: 10.1007/s11904-017-0358-8 28432579

[32] Colledge-Frisby S Ottaviano S Webb P Grebely J Wheeler A Cunningham EB . Global coverage of interventions to prevent and manage drug-related harms among people who inject drugs: a systematic review. Lancet Glob Health. (2023) 11:e673–83. doi: 10.1016/S2214-109X(23)00058-X 36996860 PMC12765503

[33] Santos-López G Panduro A Sosa-Jurado F Fierro NA Lira R Márquez-Domínguez L . Advances in the elimination of viral hepatitis in Mexico: a local perspective on the global initiative. Pathogens. (2024) 13:859. doi: 10.3390/pathogens13100859 39452730 PMC11510378

[34] Csete J Kamarulzaman A Kazatchkine M Altice F Balicki M Buxton J . Public health and international drug policy. Lancet. (2016) 387:1427–80. doi: 10.1016/S0140-6736(16)00619-X 27021149 PMC5042332

[35] Lee BP Dodge JL Leventhal A Terrault NA. Retail alcohol and tobacco sales during COVID-19. Ann Intern Med. (2021) 174:1027–9. doi: 10.7326/M20-7271 33646843 PMC7983313

[36] Asare S Majmundar A Islami F Bandi P Fedewa S Westmaas LJ . Changes in cigarette sales in the united states during the COVID-19 pandemic. Ann Intern Med. (2022) 175:141–3. doi: 10.7326/M21-3350 34662148 PMC8554710

[37] Herzig SE Albers L Soto D Lee R Ramirez C Rahman T . Pandemic-related life changes and adolescent initiation of cannabis and tobacco/nicotine use. Addict Behav. (2023) 144:107724. doi: 10.1016/j.addbeh.2023.107724 37087769 PMC10103764

[38] Amos A Haglund M. From social taboo to “torch of freedom”: the marketing of cigarettes to women. Tob Control. (2000) 9:3–8. doi: 10.1136/tc.9.1.3 10691743 PMC1748294

[39] Kisia L Mohamed SF Kyule G Tchoupé C Abolarin O Pokothoane R . Use of tobacco and nicotine products among adolescents in sub-Saharan Africa: protocol for a population-based multi-country household survey. Front Public Health. (2025) 13:1562352. doi: 10.3389/fpubh.2025.1562352 40777631 PMC12330029

[40] Adeosun A Gross K Pelikan J . Tobacco imagery in Nigerian music videos from 1999 to 2023: a content analysis. Tob Control. (2025). doi: 10.1136/tc-2025-059644 41136330

[41] Jayasinghe YA Ratnapreya S Nawarathna G Peiris PM Kanmodi KK Jayasinghe R. Prevalence and determinants of e-cigarette use in South Asia: a systematic review. BMJ Glob Health. (2025) 10:e018363. doi: 10.1136/bmjgh-2024-018363 40903043 PMC12410620

[42] McKetta SC Keyes KM. Trends in U.S. women's binge drinking in middle adulthood by socioeconomic status, 2006-2018. Drug Alcohol Depend. (2020) 212:108026. doi: 10.1016/j.drugalcdep.2020.108026 32408139 PMC7293936

[43] Slade T Chapman C Swift W Keyes K Tonks Z Teesson M. Birth cohort trends in the global epidemiology of alcohol use and alcohol-related harms in men and women: systematic review and metaregression. BMJ Open. (2016) 6:e011827. doi: 10.1136/bmjopen-2016-011827 27797998 PMC5093369

[44] Tran TT Be HT Hoang KD Dong HD. Sex and gender disparities in hepatocellular carcinoma: insights into risk, diagnosis, and therapeutic outcomes. Clin Transl Oncol. (2025) 27:4301–15. doi: 10.1007/s12094-025-03965-3 40528067

[45] Wang X Zhang L Dong B. Molecular mechanisms in MASLD/MASH-related HCC. Hepatology. (2025) 82:1303–24. doi: 10.1097/HEP.0000000000000786 38349726 PMC11323288

[46] Tzartzeva K Obi J Rich NE Parikh ND Marrero JA Yopp A . Surveillance imaging and alpha fetoprotein for early detection of hepatocellular carcinoma in patients with cirrhosis: a meta-analysis. Gastroenterology. (2018) 154:1706–18.e1. doi: 10.1053/j.gastro.2018.01.064 29425931 PMC5927818

