# Supplementary material for: The metabolic transition of liver cancer: a 34-year analysis of global burden across the 10 most populous countries, 1990–2023
Source: Front Public Health. 2026 Oct 9;14:1945748. doi: 10.3389/fpubh.2026.1945748 (PMC13690718; doi:10.3389/fpubh.2026.1945748)
Supplement: Supplementary file 1 [file Data_Sheet_1.pdf]

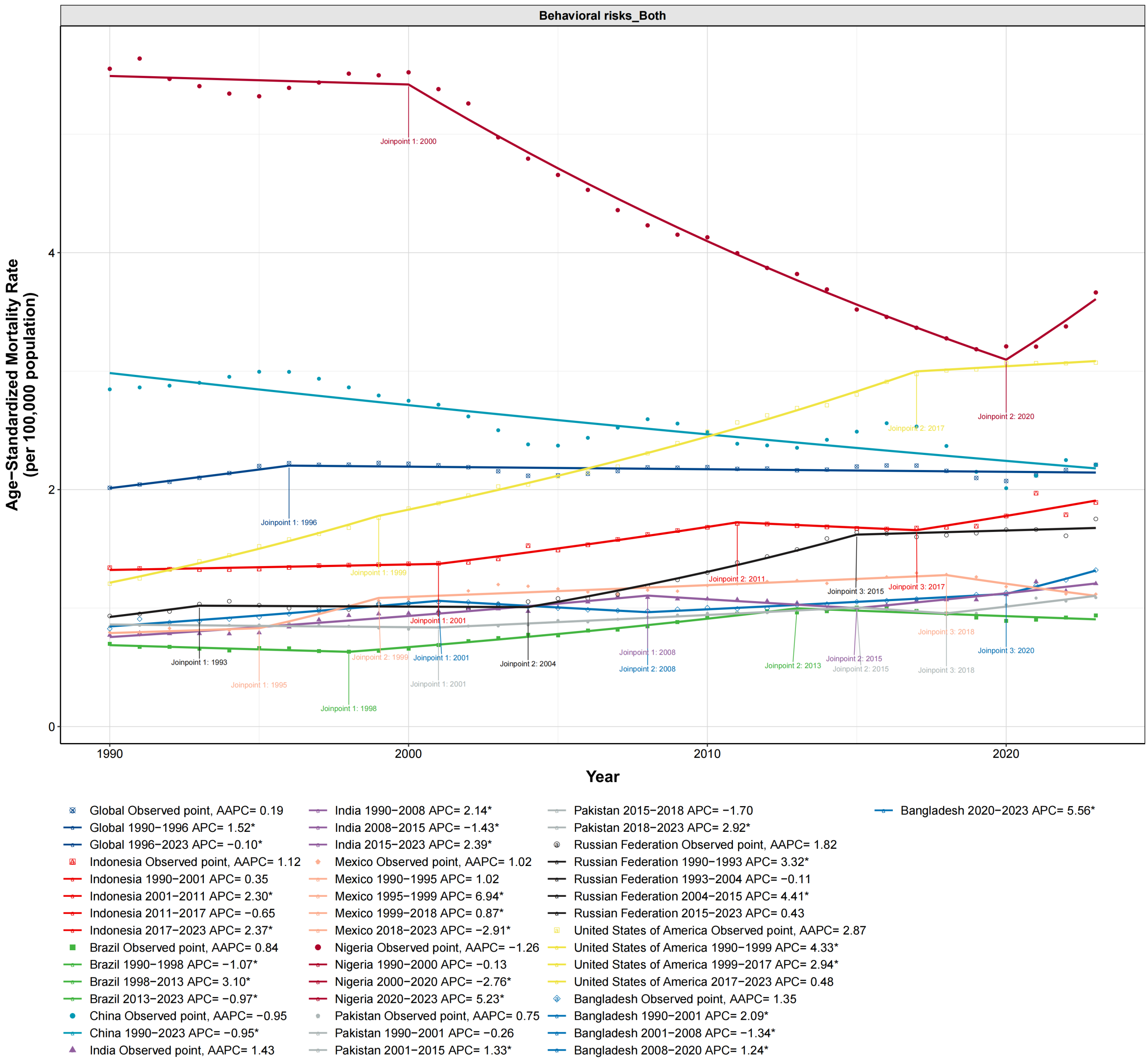
Behavioral risks_Both
Age−Standardized Mortality Rate
(per 100,000 population)
Year
0
2
4
1990
2000
2010
2020
Joinpoint 1: 2000
Joinpoint 2: 2020
Joinpoint 2: 2017
Joinpoint 1: 1996
Joinpoint 1: 1999
Joinpoint 2: 2011
Joinpoint 3: 2015
Joinpoint 3: 2017
Joinpoint 1: 2001
Joinpoint 3: 2018
Joinpoint 3: 2020
Joinpoint 2: 1999
Joinpoint 1: 2001
Joinpoint 1: 1993
Joinpoint 2: 2004
Joinpoint 1: 2008
Joinpoint 2: 2008
Joinpoint 2: 2013
Joinpoint 2: 2015
Joinpoint 2: 2015
Joinpoint 3: 2018
Joinpoint 1: 1995
Joinpoint 1: 2001
Joinpoint 1: 1998
Global Observed point, AAPC= 0.19
Global 1990−1996 APC= 1.52*
Global 1996−2023 APC= −0.10*
Indonesia Observed point, AAPC= 1.12
Indonesia 1990−2001 APC= 0.35
Indonesia 2001−2011 APC= 2.30*
Indonesia 2011−2017 APC= −0.65
Indonesia 2017−2023 APC= 2.37*
Brazil Observed point, AAPC= 0.84
Brazil 1990−1998 APC= −1.07*
Brazil 1998−2013 APC= 3.10*
Brazil 2013−2023 APC= −0.97*
China Observed point, AAPC= −0.95
China 1990−2023 APC= −0.95*
India Observed point, AAPC= 1.43
India 1990−2008 APC= 2.14*
India 2008−2015 APC= −1.43*
India 2015−2023 APC= 2.39*
Mexico Observed point, AAPC= 1.02
Mexico 1990−1995 APC= 1.02
Mexico 1995−1999 APC= 6.94*
Mexico 1999−2018 APC= 0.87*
Mexico 2018−2023 APC= −2.91*
Nigeria Observed point, AAPC= −1.26
Nigeria 1990−2000 APC= −0.13
Nigeria 2000−2020 APC= −2.76*
Nigeria 2020−2023 APC= 5.23*
Pakistan Observed point, AAPC= 0.75
Pakistan 1990−2001 APC= −0.26
Pakistan 2001−2015 APC= 1.33*
Pakistan 2015−2018 APC= −1.70
Pakistan 2018−2023 APC= 2.92*
Russian Federation Observed point, AAPC= 1.82
Russian Federation 1990−1993 APC= 3.32*
Russian Federation 1993−2004 APC= −0.11
Russian Federation 2004−2015 APC= 4.41*
Russian Federation 2015−2023 APC= 0.43
United States of America Observed point, AAPC= 2.87
United States of America 1990−1999 APC= 4.33*
United States of America 1999−2017 APC= 2.94*
United States of America 2017−2023 APC= 0.48
Bangladesh Observed point, AAPC= 1.35
Bangladesh 1990−2001 APC= 2.09*
Bangladesh 2001−2008 APC= −1.34*
Bangladesh 2008−2020 APC= 1.24*
Bangladesh 2020−2023 APC= 5.56*

Figure S1

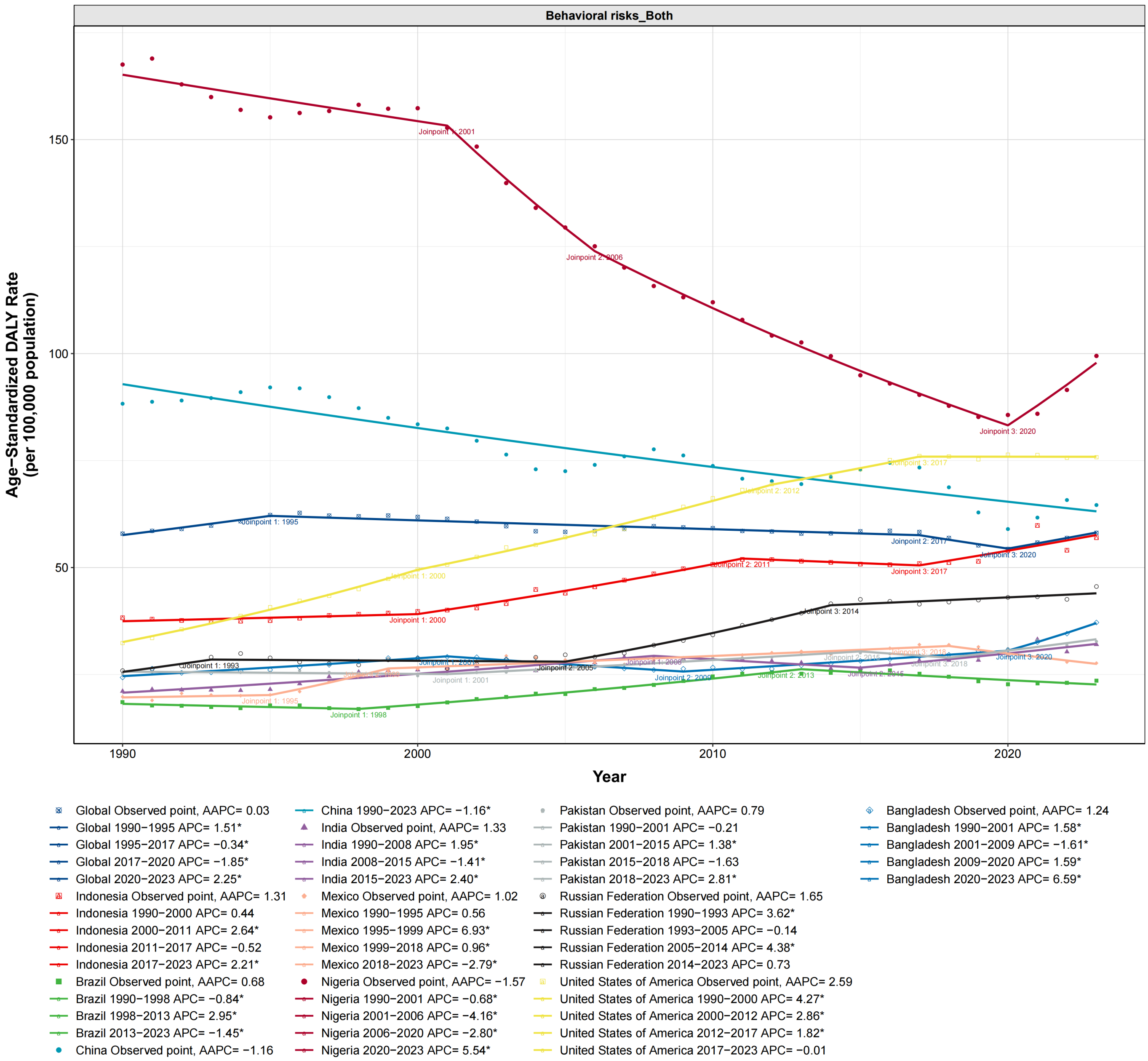
Behavioral risks_Both
Age−Standardized DALY Rate
(per 100,000 population)
150
100
50
1990
2000
2010
2020
Year
Global Observed point, AAPC= 0.03
Global 1990−1995 APC= 1.51*
Global 1995−2017 APC= −0.34*
Global 2017−2020 APC= −1.85*
Global 2020−2023 APC= 2.25*
Indonesia Observed point, AAPC= 1.31
Indonesia 1990−2000 APC= 0.44
Indonesia 2000−2011 APC= 2.64*
Indonesia 2011−2017 APC= −0.52
Indonesia 2017−2023 APC= 2.21*
Brazil Observed point, AAPC= 0.68
Brazil 1990−1998 APC= −0.84*
Brazil 1998−2013 APC= 2.95*
Brazil 2013−2023 APC= −1.45*
China Observed point, AAPC= −1.16
China 1990−2023 APC= −1.16*
India Observed point, AAPC= 1.33
India 1990−2008 APC= 1.95*
India 2008−2015 APC= −1.41*
India 2015−2023 APC= 2.40*
Mexico Observed point, AAPC= 1.02
Mexico 1990−1995 APC= 0.56
Mexico 1995−1999 APC= 6.93*
Mexico 1999−2018 APC= 0.96*
Mexico 2018−2023 APC= −2.79*
Nigeria Observed point, AAPC= −1.57
Nigeria 1990−2001 APC= −0.68*
Nigeria 2001−2006 APC= −4.16*
Nigeria 2006−2020 APC= −2.80*
Nigeria 2020−2023 APC= 5.54*
Pakistan Observed point, AAPC= 0.79
Pakistan 1990−2001 APC= −0.21
Pakistan 2001−2015 APC= 1.38*
Pakistan 2015−2018 APC= −1.63
Pakistan 2018−2023 APC= 2.81*
Russian Federation Observed point, AAPC= 1.65
Russian Federation 1990−1993 APC= 3.62*
Russian Federation 1993−2005 APC= −0.14
Russian Federation 2005−2014 APC= 4.38*
Russian Federation 2014−2023 APC= 0.73
United States of America Observed point, AAPC= 2.59
United States of America 1990−2000 APC= 4.27*
United States of America 2000−2012 APC= 2.86*
United States of America 2012−2017 APC= 1.82*
United States of America 2017−2023 APC= −0.01
Bangladesh Observed point, AAPC= 1.24
Bangladesh 1990−2001 APC= 1.58*
Bangladesh 2001−2009 APC= −1.61*
Bangladesh 2009−2020 APC= 1.59*
Bangladesh 2020−2023 APC= 6.59*

Figure S2

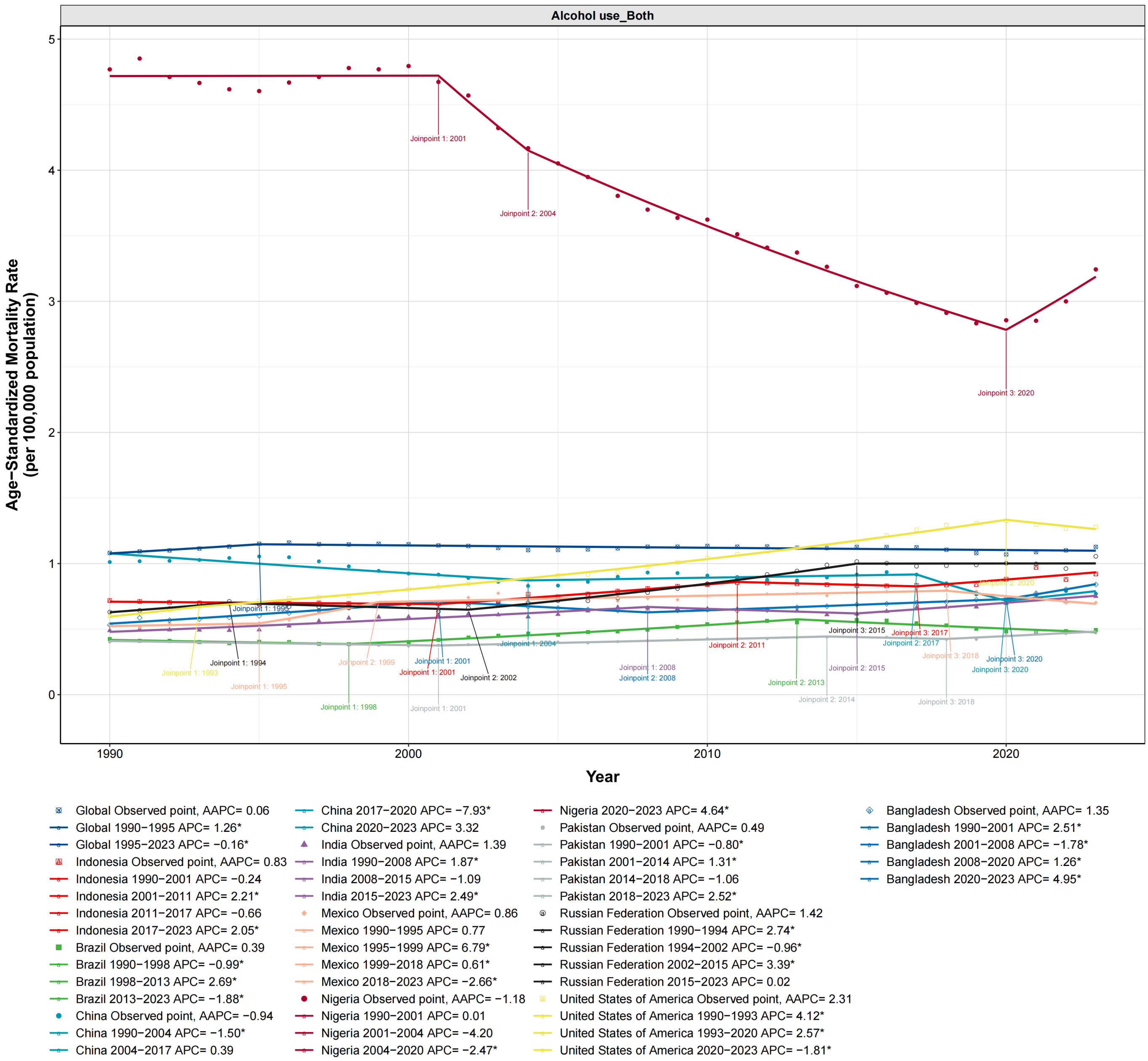
Alcohol use_Both
Age−Standardized Mortality Rate
(per 100,000 population)
Year
Global Observed point, AAPC= 0.06
Global 1990−1995 APC= 1.26*
Global 1995−2023 APC= −0.16*
Indonesia Observed point, AAPC= 0.83
Indonesia 1990−2001 APC= −0.24
Indonesia 2001−2011 APC= 2.21*
Indonesia 2011−2017 APC= −0.66
Indonesia 2017−2023 APC= 2.05*
Brazil Observed point, AAPC= 0.39
Brazil 1990−1998 APC= −0.99*
Brazil 1998−2013 APC= 2.69*
Brazil 2013−2023 APC= −1.88*
China Observed point, AAPC= −0.94
China 1990−2004 APC= −1.50*
China 2004−2017 APC= 0.39
China 2017−2020 APC= −7.93*
China 2020−2023 APC= 3.32
India Observed point, AAPC= 1.39
India 1990−2008 APC= 1.87*
India 2008−2015 APC= −1.09
India 2015−2023 APC= 2.49*
Mexico Observed point, AAPC= 0.86
Mexico 1990−1995 APC= 0.77
Mexico 1995−1999 APC= 6.79*
Mexico 1999−2018 APC= 0.61*
Mexico 2018−2023 APC= −2.66*
Nigeria Observed point, AAPC= −1.18
Nigeria 1990−2001 APC= 0.01
Nigeria 2001−2004 APC= −4.20
Nigeria 2004−2020 APC= −2.47*
Nigeria 2020−2023 APC= 4.64*
Pakistan Observed point, AAPC= 0.49
Pakistan 1990−2001 APC= −0.80*
Pakistan 2001−2014 APC= 1.31*
Pakistan 2014−2018 APC= −1.06
Pakistan 2018−2023 APC= 2.52*
Russian Federation Observed point, AAPC= 1.42
Russian Federation 1990−1994 APC= 2.74*
Russian Federation 1994−2002 APC= −0.96*
Russian Federation 2002−2015 APC= 3.39*
Russian Federation 2015−2023 APC= 0.02
United States of America Observed point, AAPC= 2.31
United States of America 1990−1993 APC= 4.12*
United States of America 1993−2020 APC= 2.57*
United States of America 2020−2023 APC= −1.81*
Bangladesh Observed point, AAPC= 1.35
Bangladesh 1990−2001 APC= 2.51*
Bangladesh 2001−2008 APC= −1.78*
Bangladesh 2008−2020 APC= 1.26*
Bangladesh 2020−2023 APC= 4.95*

Figure S3

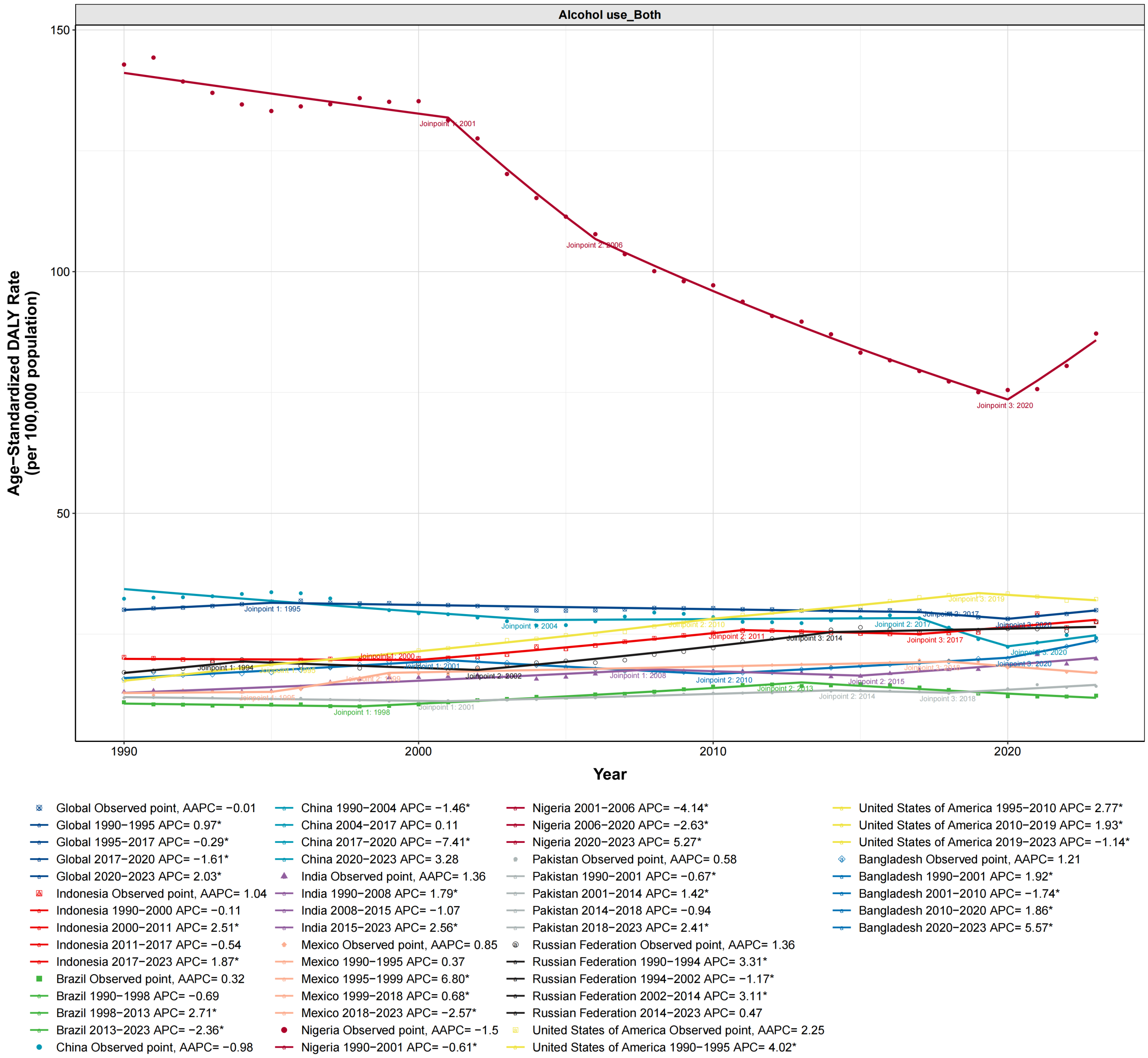
Alcohol use_Both
150
100
50
1990
2000
2010
2020
Year
Age−Standardized DALY Rate
(per 100,000 population)
Joinpoint 1: 2001
Joinpoint 2: 2006
Joinpoint 3: 2020
Global Observed point, AAPC= −0.01
Global 1990−1995 APC= 0.97*
Global 1995−2017 APC= −0.29*
Global 2017−2020 APC= −1.61*
Global 2020−2023 APC= 2.03*
Indonesia Observed point, AAPC= 1.04
Indonesia 1990−2000 APC= −0.11
Indonesia 2000−2011 APC= 2.51*
Indonesia 2011−2017 APC= −0.54
Indonesia 2017−2023 APC= 1.87*
Brazil Observed point, AAPC= 0.32
Brazil 1990−1998 APC= −0.69
Brazil 1998−2013 APC= 2.71*
Brazil 2013−2023 APC= −2.36*
China Observed point, AAPC= −0.98
China 1990−2004 APC= −1.46*
China 2004−2017 APC= 0.11
China 2017−2020 APC= −7.41*
China 2020−2023 APC= 3.28
India Observed point, AAPC= 1.36
India 1990−2008 APC= 1.79*
India 2008−2015 APC= −1.07
India 2015−2023 APC= 2.56*
Mexico Observed point, AAPC= 0.85
Mexico 1990−1995 APC= 0.37
Mexico 1995−1999 APC= 6.80*
Mexico 1999−2018 APC= 0.68*
Mexico 2018−2023 APC= −2.57*
Nigeria Observed point, AAPC= −1.5
Nigeria 1990−2001 APC= −0.61*
Nigeria 2001−2006 APC= −4.14*
Nigeria 2006−2020 APC= −2.63*
Nigeria 2020−2023 APC= 5.27*
Pakistan Observed point, AAPC= 0.58
Pakistan 1990−2001 APC= −0.67*
Pakistan 2001−2014 APC= 1.42*
Pakistan 2014−2018 APC= −0.94
Pakistan 2018−2023 APC= 2.41*
Russian Federation Observed point, AAPC= 1.36
Russian Federation 1990−1994 APC= 3.31*
Russian Federation 1994−2002 APC= −1.17*
Russian Federation 2002−2014 APC= 3.11*
Russian Federation 2014−2023 APC= 0.47
United States of America Observed point, AAPC= 2.25
United States of America 1990−1995 APC= 4.02*
United States of America 1995−2010 APC= 2.77*
United States of America 2010−2019 APC= 1.93*
United States of America 2019−2023 APC= −1.14*
Bangladesh Observed point, AAPC= 1.21
Bangladesh 1990−2001 APC= 1.92*
Bangladesh 2001−2010 APC= −1.74*
Bangladesh 2010−2020 APC= 1.86*
Bangladesh 2020−2023 APC= 5.57*

Figure S4

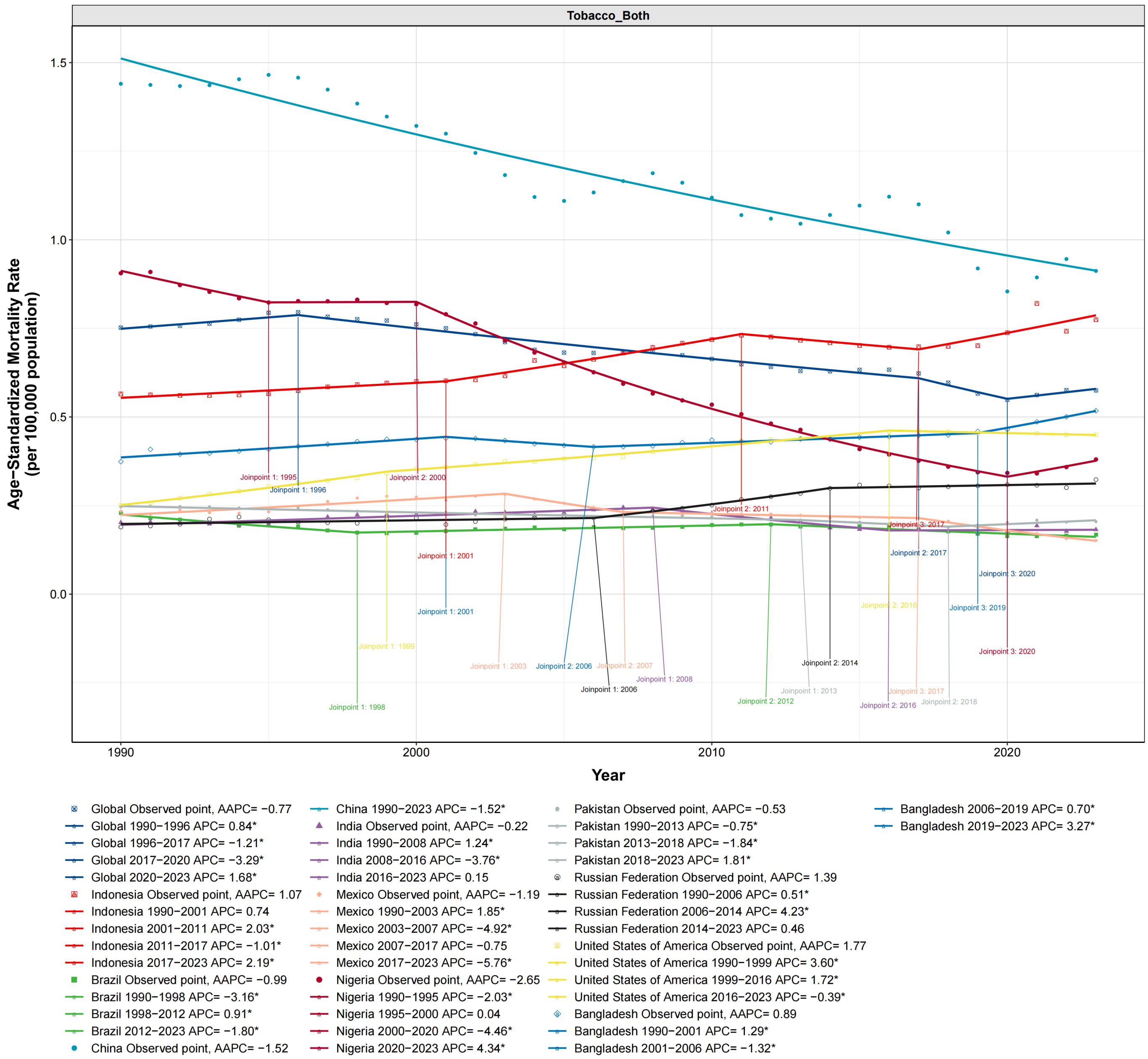
Tobacco_Both
Age−Standardized Mortality Rate
(per 100,000 population)
1.5
1.0
0.5
0.0
1990
2000
2010
2020
Year
Global Observed point, AAPC= −0.77
Global 1990−1996 APC= 0.84*
Global 1996−2017 APC= −1.21*
Global 2017−2020 APC= −3.29*
Global 2020−2023 APC= 1.68*
Indonesia Observed point, AAPC= 1.07
Indonesia 1990−2001 APC= 0.74
Indonesia 2001−2011 APC= 2.03*
Indonesia 2011−2017 APC= −1.01*
Indonesia 2017−2023 APC= 2.19*
Brazil Observed point, AAPC= −0.99
Brazil 1990−1998 APC= −3.16*
Brazil 1998−2012 APC= 0.91*
Brazil 2012−2023 APC= −1.80*
China Observed point, AAPC= −1.52
China 1990−2023 APC= −1.52*
India Observed point, AAPC= −0.22
India 1990−2008 APC= 1.24*
India 2008−2016 APC= −3.76*
India 2016−2023 APC= 0.15
Mexico Observed point, AAPC= −1.19
Mexico 1990−2003 APC= 1.85*
Mexico 2003−2007 APC= −4.92*
Mexico 2007−2017 APC= −0.75
Mexico 2017−2023 APC= −5.76*
Nigeria Observed point, AAPC= −2.65
Nigeria 1990−1995 APC= −2.03*
Nigeria 1995−2000 APC= 0.04
Nigeria 2000−2020 APC= −4.46*
Nigeria 2020−2023 APC= 4.34*
Pakistan Observed point, AAPC= −0.53
Pakistan 1990−2013 APC= −0.75*
Pakistan 2013−2018 APC= −1.84*
Pakistan 2018−2023 APC= 1.81*
Russian Federation Observed point, AAPC= 1.39
Russian Federation 1990−2006 APC= 0.51*
Russian Federation 2006−2014 APC= 4.23*
Russian Federation 2014−2023 APC= 0.46
United States of America Observed point, AAPC= 1.77
United States of America 1990−1999 APC= 3.60*
United States of America 1999−2016 APC= 1.72*
United States of America 2016−2023 APC= −0.39*
Bangladesh Observed point, AAPC= 0.89
Bangladesh 1990−2001 APC= 1.29*
Bangladesh 2001−2006 APC= −1.32*
Bangladesh 2006−2019 APC= 0.70*
Bangladesh 2019−2023 APC= 3.27*

Figure S5

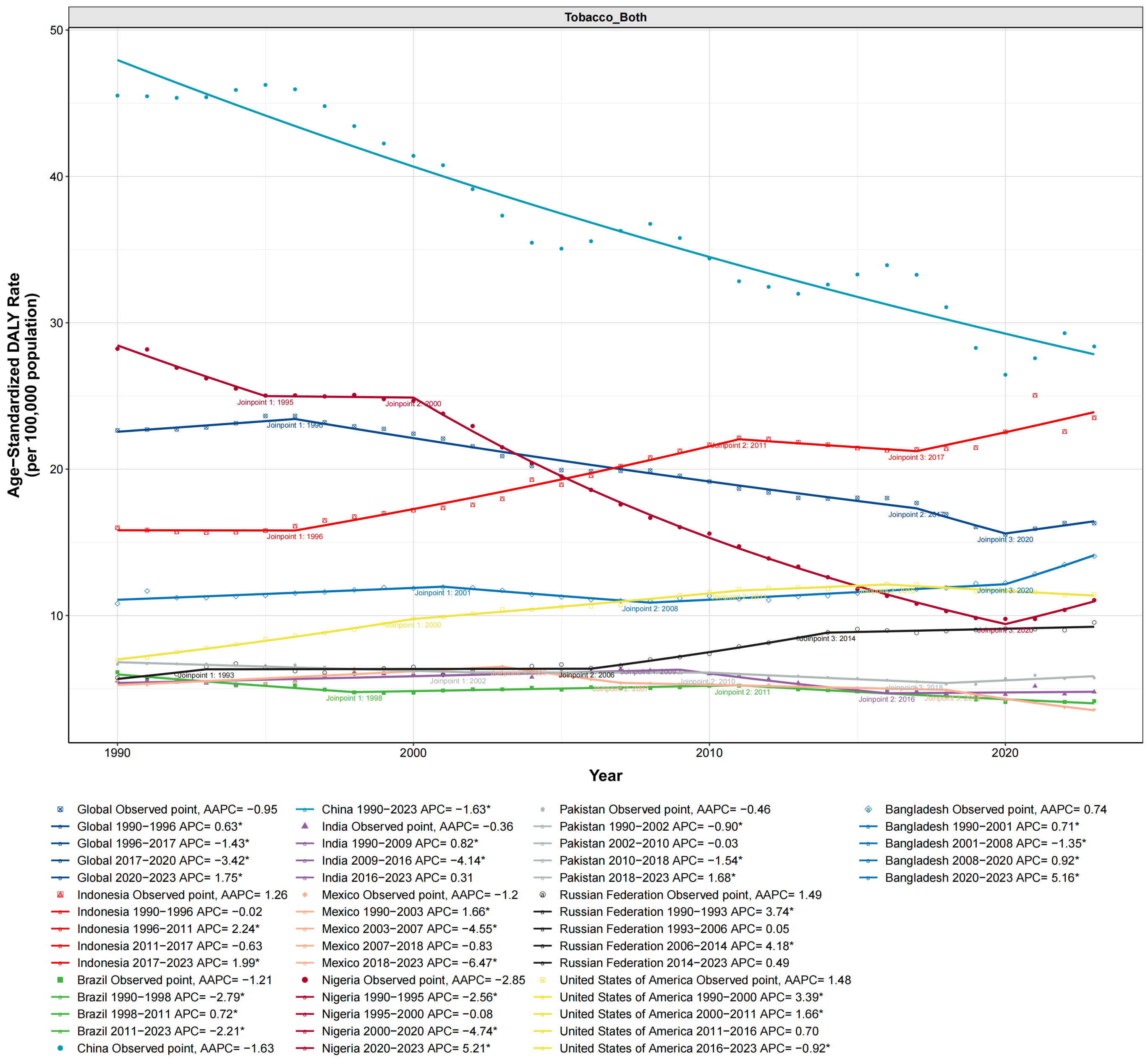

Tobacco_Both
Age−Standardized DALY Rate
(per 100,000 population)
50
40
30
20
10
1990
2000
2010
2020
Year
Joinpoint 1: 1995
Joinpoint 2: 2000
Joinpoint 1: 1996
Joinpoint 2: 2011
Joinpoint 3: 2017
Joinpoint 1: 1996
Joinpoint 2: 2017
Joinpoint 3: 2020
Joinpoint 1: 2001
Joinpoint 2: 2008
Joinpoint 3: 2020
Joinpoint 1: 2000
Joinpoint 3: 2014
Joinpoint 3: 2020
Joinpoint 1: 1993
Joinpoint 2: 2006
Joinpoint 1: 2002
Joinpoint 2: 2010
Joinpoint 1: 1998
Joinpoint 2: 2011
Joinpoint 2: 2016
Global Observed point, AAPC= −0.95
Global 1990−1996 APC= 0.63*
Global 1996−2017 APC= −1.43*
Global 2017−2020 APC= −3.42*
Global 2020−2023 APC= 1.75*
Indonesia Observed point, AAPC= 1.26
Indonesia 1990−1996 APC= −0.02
Indonesia 1996−2011 APC= 2.24*
Indonesia 2011−2017 APC= −0.63
Indonesia 2017−2023 APC= 1.99*
Brazil Observed point, AAPC= −1.21
Brazil 1990−1998 APC= −2.79*
Brazil 1998−2011 APC= 0.72*
Brazil 2011−2023 APC= −2.21*
China Observed point, AAPC= −1.63
China 1990−2023 APC= −1.63*
India Observed point, AAPC= −0.36
India 1990−2009 APC= 0.82*
India 2009−2016 APC= −4.14*
India 2016−2023 APC= 0.31
Mexico Observed point, AAPC= −1.2
Mexico 1990−2003 APC= 1.66*
Mexico 2003−2007 APC= −4.55*
Mexico 2007−2018 APC= −0.83
Mexico 2018−2023 APC= −6.47*
Nigeria Observed point, AAPC= −2.85
Nigeria 1990−1995 APC= −2.56*
Nigeria 1995−2000 APC= −0.08
Nigeria 2000−2020 APC= −4.74*
Nigeria 2020−2023 APC= 5.21*
Pakistan Observed point, AAPC= −0.46
Pakistan 1990−2002 APC= −0.90*
Pakistan 2002−2010 APC= −0.03
Pakistan 2010−2018 APC= −1.54*
Pakistan 2018−2023 APC= 1.68*
Russian Federation Observed point, AAPC= 1.49
Russian Federation 1990−1993 APC= 3.74*
Russian Federation 1993−2006 APC= 0.05
Russian Federation 2006−2014 APC= 4.18*
Russian Federation 2014−2023 APC= 0.49
United States of America Observed point, AAPC= 1.48
United States of America 1990−2000 APC= 3.39*
United States of America 2000−2011 APC= 1.66*
United States of America 2011−2016 APC= 0.70
United States of America 2016−2023 APC= −0.92*
Bangladesh Observed point, AAPC= 0.74
Bangladesh 1990−2001 APC= 0.71*
Bangladesh 2001−2008 APC= −1.35*
Bangladesh 2008−2020 APC= 0.92*
Bangladesh 2020−2023 APC= 5.16*

Figure S6

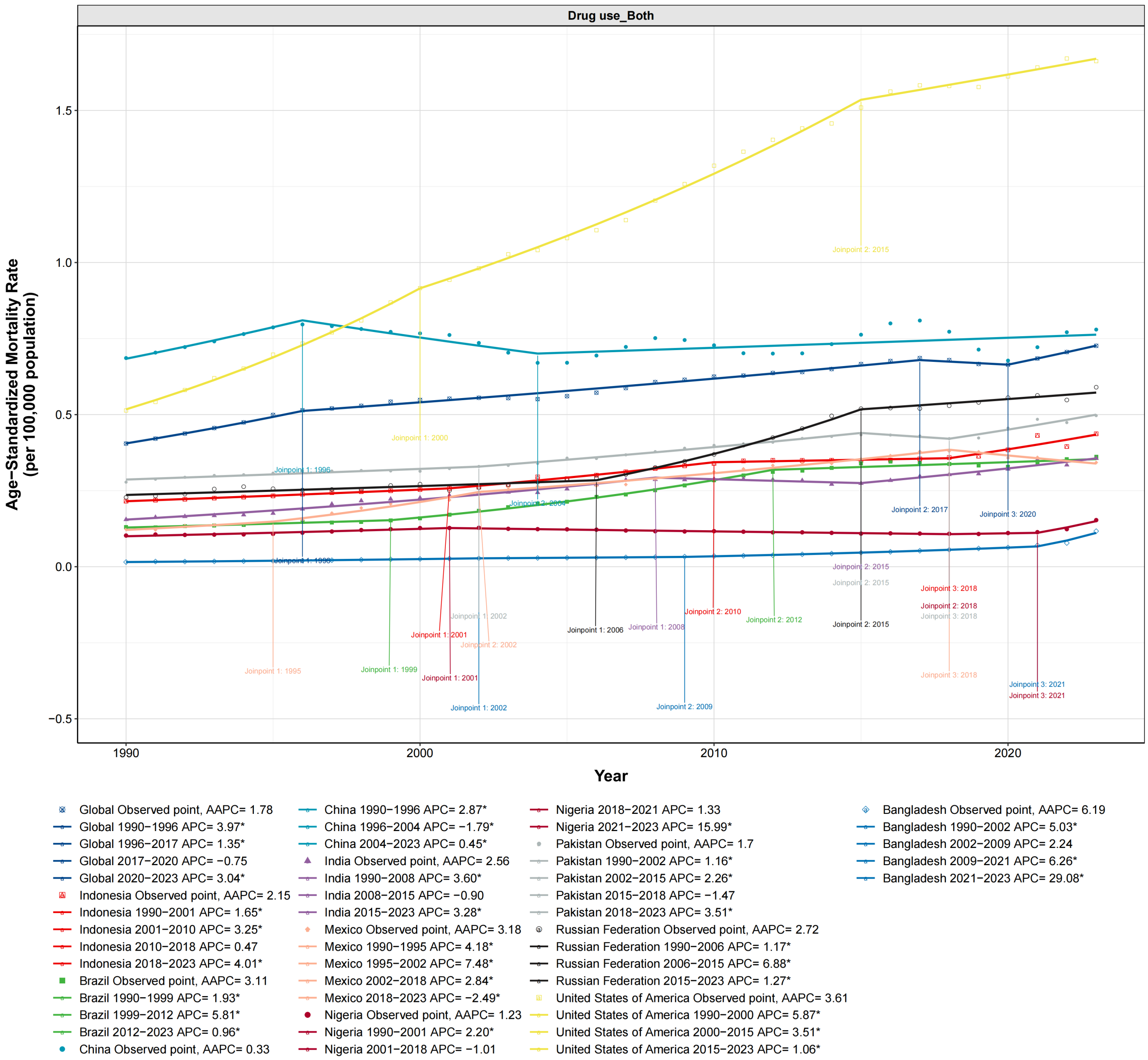

Drug use_Both
Age−Standardized Mortality Rate (per 100,000 population)
1.5
1.0
0.5
0.0
−0.5
1990
2000
2010
2020
Year
Global Observed point, AAPC= 1.78
Global 1990−1996 APC= 3.97*
Global 1996−2017 APC= 1.35*
Global 2017−2020 APC= −0.75
Global 2020−2023 APC= 3.04*
Indonesia Observed point, AAPC= 2.15
Indonesia 1990−2001 APC= 1.65*
Indonesia 2001−2010 APC= 3.25*
Indonesia 2010−2018 APC= 0.47
Indonesia 2018−2023 APC= 4.01*
Brazil Observed point, AAPC= 3.11
Brazil 1990−1999 APC= 1.93*
Brazil 1999−2012 APC= 5.81*
Brazil 2012−2023 APC= 0.96*
China Observed point, AAPC= 0.33
China 1990−1996 APC= 2.87*
China 1996−2004 APC= −1.79*
China 2004−2023 APC= 0.45*
India Observed point, AAPC= 2.56
India 1990−2008 APC= 3.60*
India 2008−2015 APC= −0.90
India 2015−2023 APC= 3.28*
Mexico Observed point, AAPC= 3.18
Mexico 1990−1995 APC= 4.18*
Mexico 1995−2002 APC= 7.48*
Mexico 2002−2018 APC= 2.84*
Mexico 2018−2023 APC= −2.49*
Nigeria Observed point, AAPC= 1.23
Nigeria 1990−2001 APC= 2.20*
Nigeria 2001−2018 APC= −1.01
Nigeria 2018−2021 APC= 1.33
Nigeria 2021−2023 APC= 15.99*
Pakistan Observed point, AAPC= 1.7
Pakistan 1990−2002 APC= 1.16*
Pakistan 2002−2015 APC= 2.26*
Pakistan 2015−2018 APC= −1.47
Pakistan 2018−2023 APC= 3.51*
Russian Federation Observed point, AAPC= 2.72
Russian Federation 1990−2006 APC= 1.17*
Russian Federation 2006−2015 APC= 6.88*
Russian Federation 2015−2023 APC= 1.27*
United States of America Observed point, AAPC= 3.61
United States of America 1990−2000 APC= 5.87*
United States of America 2000−2015 APC= 3.51*
United States of America 2015−2023 APC= 1.06*
Bangladesh Observed point, AAPC= 6.19
Bangladesh 1990−2002 APC= 5.03*
Bangladesh 2002−2009 APC= 2.24
Bangladesh 2009−2021 APC= 6.26*
Bangladesh 2021−2023 APC= 29.08*

Figure S7

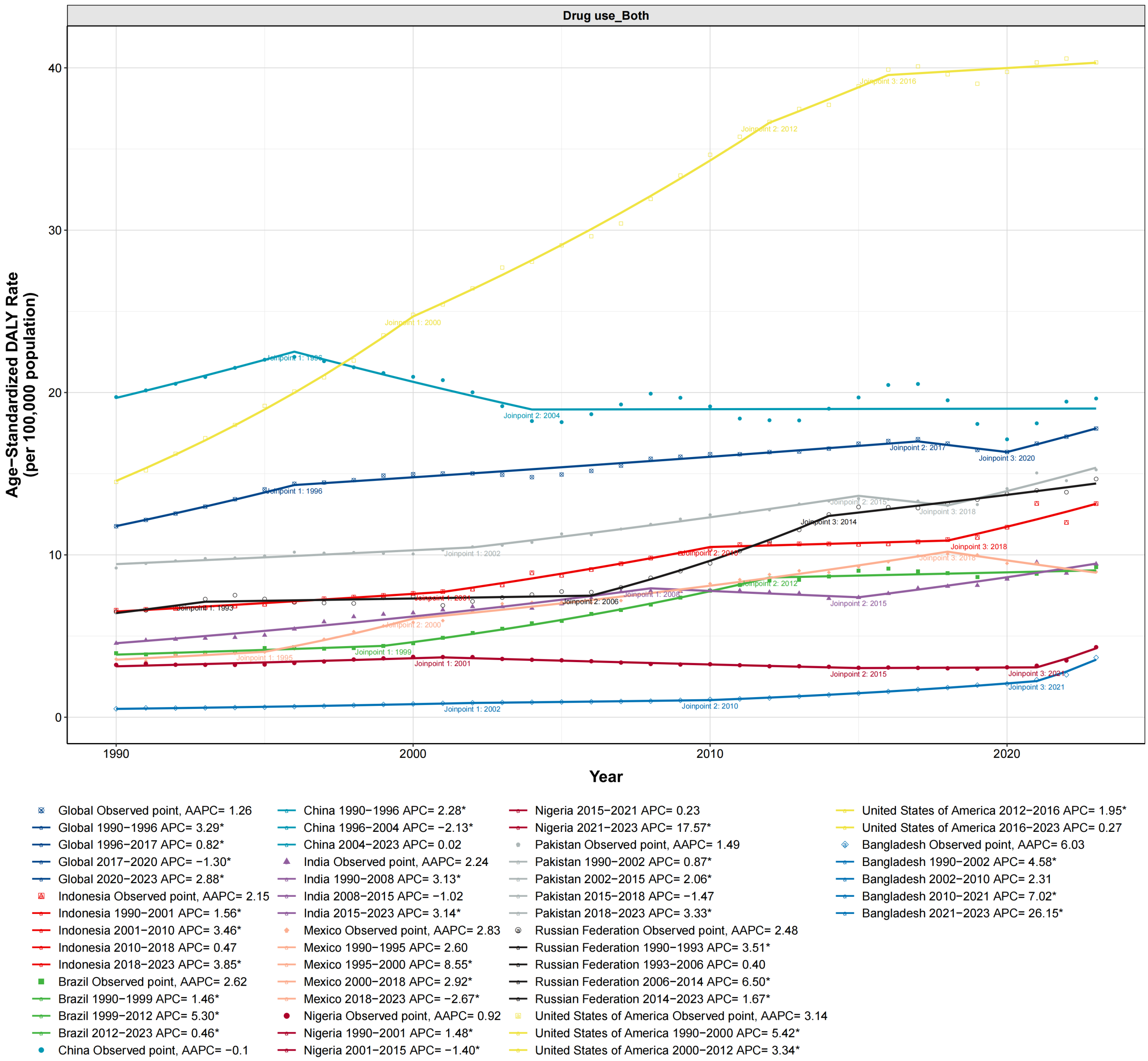
Drug use_Both
Age−Standardized DALY Rate (per 100,000 population)
Year
1990
2000
2010
2020
0
10
20
30
40
Global Observed point, AAPC= 1.26
Global 1990−1996 APC= 3.29*
Global 1996−2017 APC= 0.82*
Global 2017−2020 APC= −1.30*
Global 2020−2023 APC= 2.88*
Indonesia Observed point, AAPC= 2.15
Indonesia 1990−2001 APC= 1.56*
Indonesia 2001−2010 APC= 3.46*
Indonesia 2010−2018 APC= 0.47
Indonesia 2018−2023 APC= 3.85*
Brazil Observed point, AAPC= 2.62
Brazil 1990−1999 APC= 1.46*
Brazil 1999−2012 APC= 5.30*
Brazil 2012−2023 APC= 0.46*
China Observed point, AAPC= −0.1
China 1990−1996 APC= 2.28*
China 1996−2004 APC= −2.13*
China 2004−2023 APC= 0.02
India Observed point, AAPC= 2.24
India 1990−2008 APC= 3.13*
India 2008−2015 APC= −1.02
India 2015−2023 APC= 3.14*
Mexico Observed point, AAPC= 2.83
Mexico 1990−1995 APC= 2.60
Mexico 1995−2000 APC= 8.55*
Mexico 2000−2018 APC= 2.92*
Mexico 2018−2023 APC= −2.67*
Nigeria Observed point, AAPC= 0.92
Nigeria 1990−2001 APC= 1.48*
Nigeria 2001−2015 APC= −1.40*
Nigeria 2015−2021 APC= 0.23
Nigeria 2021−2023 APC= 17.57*
Pakistan Observed point, AAPC= 1.49
Pakistan 1990−2002 APC= 0.87*
Pakistan 2002−2015 APC= 2.06*
Pakistan 2015−2018 APC= −1.47
Pakistan 2018−2023 APC= 3.33*
Russian Federation Observed point, AAPC= 2.48
Russian Federation 1990−1993 APC= 3.51*
Russian Federation 1993−2006 APC= 0.40
Russian Federation 2006−2014 APC= 6.50*
Russian Federation 2014−2023 APC= 1.67*
United States of America Observed point, AAPC= 3.14
United States of America 1990−2000 APC= 5.42*
United States of America 2000−2012 APC= 3.34*
United States of America 2012−2016 APC= 1.95*
United States of America 2016−2023 APC= 0.27
Bangladesh Observed point, AAPC= 6.03
Bangladesh 1990−2002 APC= 4.58*
Bangladesh 2002−2010 APC= 2.31
Bangladesh 2010−2021 APC= 7.02*
Bangladesh 2021−2023 APC= 26.15*

Figure S8

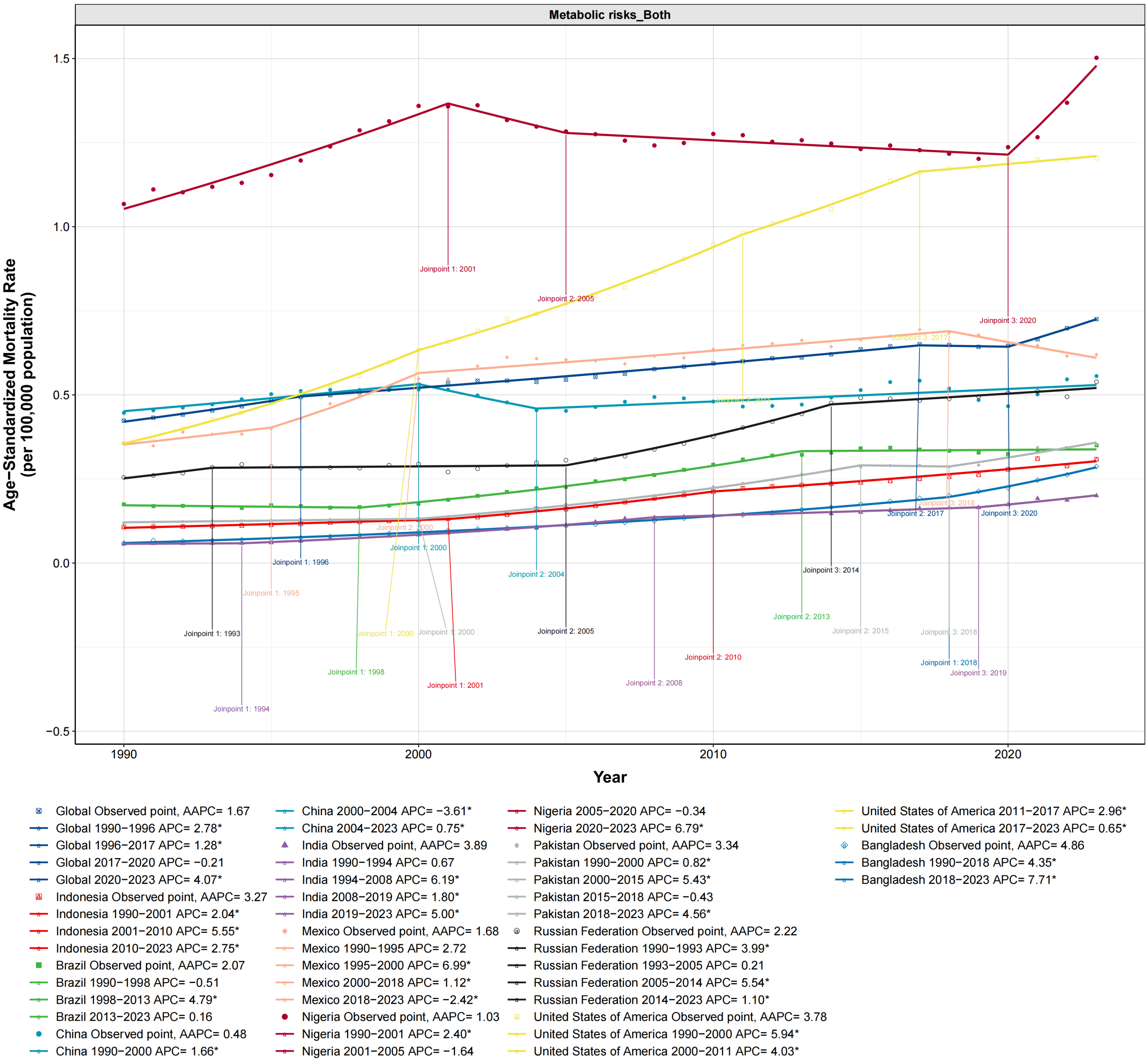

Metabolic risks_Both
Age−Standardized Mortality Rate
(per 100,000 population)
Year
1.5
1.0
0.5
0.0
−0.5
1990
2000
2010
2020
Joinpoint 1: 2001
Joinpoint 2: 2005
Joinpoint 3: 2020
Joinpoint 1: 1996
Joinpoint 1: 2000
Joinpoint 2: 2004
Joinpoint 1: 1995
Joinpoint 1: 1993
Joinpoint 2: 2005
Joinpoint 3: 2014
Joinpoint 2: 2013
Joinpoint 2: 2015
Joinpoint 3: 2018
Joinpoint 1: 2018
Joinpoint 3: 2019
Joinpoint 2: 2017
Joinpoint 3: 2020
Joinpoint 2: 2010
Joinpoint 2: 2008
Joinpoint 1: 1998
Joinpoint 1: 1994
Joinpoint 1: 2001
Global Observed point, AAPC= 1.67
Global 1990−1996 APC= 2.78*
Global 1996−2017 APC= 1.28*
Global 2017−2020 APC= −0.21
Global 2020−2023 APC= 4.07*
Indonesia Observed point, AAPC= 3.27
Indonesia 1990−2001 APC= 2.04*
Indonesia 2001−2010 APC= 5.55*
Indonesia 2010−2023 APC= 2.75*
Brazil Observed point, AAPC= 2.07
Brazil 1990−1998 APC= −0.51
Brazil 1998−2013 APC= 4.79*
Brazil 2013−2023 APC= 0.16
China Observed point, AAPC= 0.48
China 1990−2000 APC= 1.66*
China 2000−2004 APC= −3.61*
China 2004−2023 APC= 0.75*
India Observed point, AAPC= 3.89
India 1990−1994 APC= 0.67
India 1994−2008 APC= 6.19*
India 2008−2019 APC= 1.80*
India 2019−2023 APC= 5.00*
Mexico Observed point, AAPC= 1.68
Mexico 1990−1995 APC= 2.72
Mexico 1995−2000 APC= 6.99*
Mexico 2000−2018 APC= 1.12*
Mexico 2018−2023 APC= −2.42*
Nigeria Observed point, AAPC= 1.03
Nigeria 1990−2001 APC= 2.40*
Nigeria 2001−2005 APC= −1.64
Nigeria 2005−2020 APC= −0.34
Nigeria 2020−2023 APC= 6.79*
Pakistan Observed point, AAPC= 3.34
Pakistan 1990−2000 APC= 0.82*
Pakistan 2000−2015 APC= 5.43*
Pakistan 2015−2018 APC= −0.43
Pakistan 2018−2023 APC= 4.56*
Russian Federation Observed point, AAPC= 2.22
Russian Federation 1990−1993 APC= 3.99*
Russian Federation 1993−2005 APC= 0.21
Russian Federation 2005−2014 APC= 5.54*
Russian Federation 2014−2023 APC= 1.10*
United States of America Observed point, AAPC= 3.78
United States of America 1990−2000 APC= 5.94*
United States of America 2000−2011 APC= 4.03*
United States of America 2011−2017 APC= 2.96*
United States of America 2017−2023 APC= 0.65*
Bangladesh Observed point, AAPC= 4.86
Bangladesh 1990−2018 APC= 4.35*
Bangladesh 2018−2023 APC= 7.71*

Figure S9

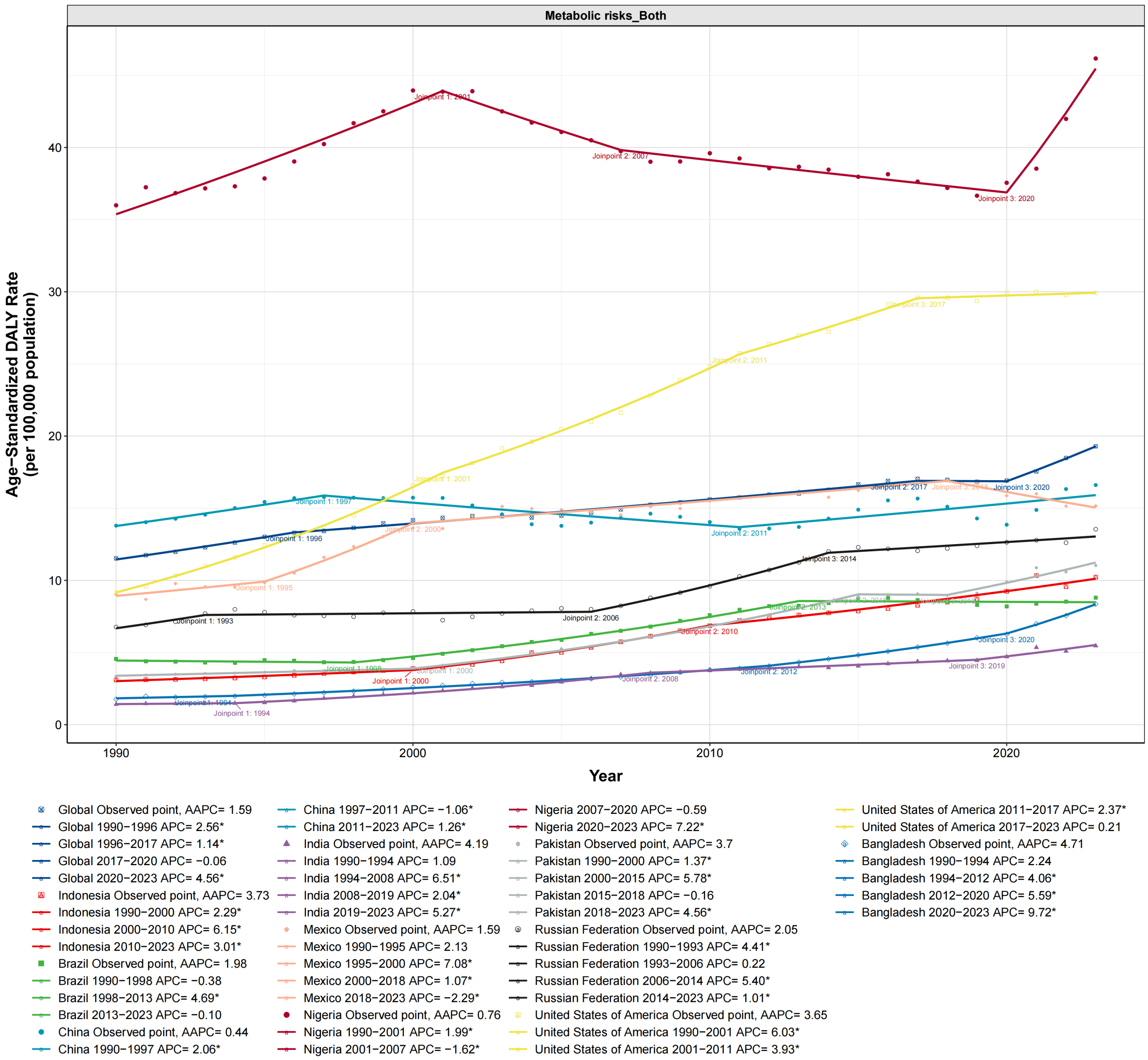
Metabolic risks_Both
Age−Standardized DALY Rate (per 100,000 population)
Year
0
10
20
30
40
1990
2000
2010
2020
Global Observed point, AAPC= 1.59
Global 1990−1996 APC= 2.56*
Global 1996−2017 APC= 1.14*
Global 2017−2020 APC= −0.06
Global 2020−2023 APC= 4.56*
Indonesia Observed point, AAPC= 3.73
Indonesia 1990−2000 APC= 2.29*
Indonesia 2000−2010 APC= 6.15*
Indonesia 2010−2023 APC= 3.01*
Brazil Observed point, AAPC= 1.98
Brazil 1990−1998 APC= −0.38
Brazil 1998−2013 APC= 4.69*
Brazil 2013−2023 APC= −0.10
China Observed point, AAPC= 0.44
China 1990−1997 APC= 2.06*
China 1997−2011 APC= −1.06*
China 2011−2023 APC= 1.26*
India Observed point, AAPC= 4.19
India 1990−1994 APC= 1.09
India 1994−2008 APC= 6.51*
India 2008−2019 APC= 2.04*
India 2019−2023 APC= 5.27*
Mexico Observed point, AAPC= 1.59
Mexico 1990−1995 APC= 2.13
Mexico 1995−2000 APC= 7.08*
Mexico 2000−2018 APC= 1.07*
Mexico 2018−2023 APC= −2.29*
Nigeria Observed point, AAPC= 0.76
Nigeria 1990−2001 APC= 1.99*
Nigeria 2001−2007 APC= −1.62*
Nigeria 2007−2020 APC= −0.59
Nigeria 2020−2023 APC= 7.22*
Pakistan Observed point, AAPC= 3.7
Pakistan 1990−2000 APC= 1.37*
Pakistan 2000−2015 APC= 5.78*
Pakistan 2015−2018 APC= −0.16
Pakistan 2018−2023 APC= 4.56*
Russian Federation Observed point, AAPC= 2.05
Russian Federation 1990−1993 APC= 4.41*
Russian Federation 1993−2006 APC= 0.22
Russian Federation 2006−2014 APC= 5.40*
Russian Federation 2014−2023 APC= 1.01*
United States of America Observed point, AAPC= 3.65
United States of America 1990−2001 APC= 6.03*
United States of America 2001−2011 APC= 3.93*
United States of America 2011−2017 APC= 2.37*
United States of America 2017−2023 APC= 0.21
Bangladesh Observed point, AAPC= 4.71
Bangladesh 1990−1994 APC= 2.24
Bangladesh 1994−2012 APC= 4.06*
Bangladesh 2012−2020 APC= 5.59*
Bangladesh 2020−2023 APC= 9.72*

Figure S10

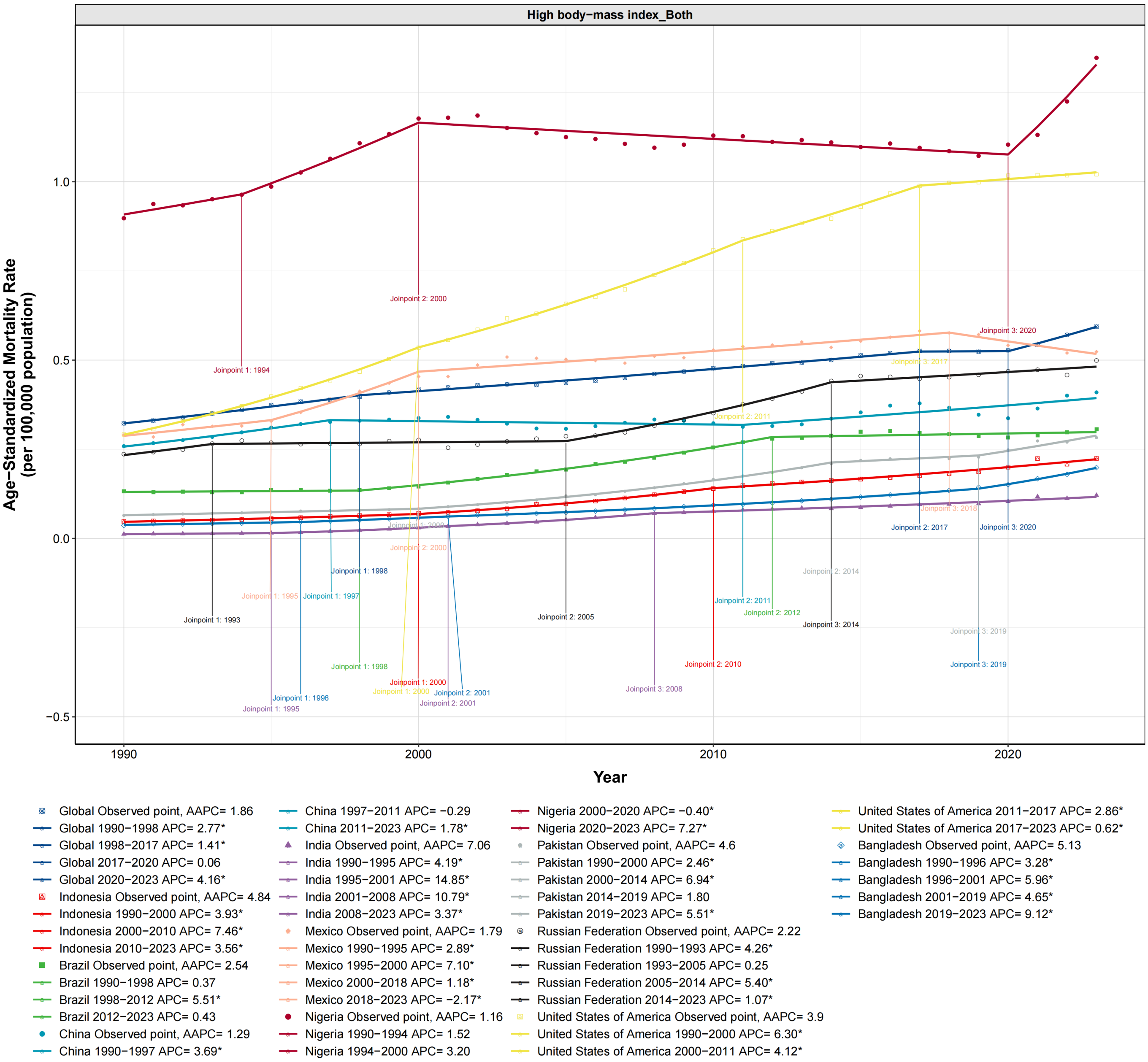
High body−mass index_Both
Age−Standardized Mortality Rate
(per 100,000 population)
1.0
0.5
0.0
−0.5
1990
2000
2010
2020
Year
Joinpoint 2: 2000
Joinpoint 1: 1994
Joinpoint 3: 2020
Joinpoint 2: 2011
Joinpoint 3: 2017
Joinpoint 3: 2018
Joinpoint 2: 2017
Joinpoint 3: 2020
Joinpoint 1: 2000
Joinpoint 2: 2000
Joinpoint 1: 1998
Joinpoint 1: 1995
Joinpoint 1: 1997
Joinpoint 1: 1993
Joinpoint 2: 2005
Joinpoint 2: 2014
Joinpoint 2: 2011
Joinpoint 2: 2012
Joinpoint 3: 2014
Joinpoint 3: 2019
Joinpoint 1: 1998
Joinpoint 2: 2010
Joinpoint 3: 2019
Joinpoint 1: 2000
Joinpoint 1: 1996
Joinpoint 1: 2000
Joinpoint 2: 2001
Joinpoint 2: 2001
Joinpoint 3: 2008
Joinpoint 1: 1995
Global Observed point, AAPC= 1.86
Global 1990−1998 APC= 2.77*
Global 1998−2017 APC= 1.41*
Global 2017−2020 APC= 0.06
Global 2020−2023 APC= 4.16*
Indonesia Observed point, AAPC= 4.84
Indonesia 1990−2000 APC= 3.93*
Indonesia 2000−2010 APC= 7.46*
Indonesia 2010−2023 APC= 3.56*
Brazil Observed point, AAPC= 2.54
Brazil 1990−1998 APC= 0.37
Brazil 1998−2012 APC= 5.51*
Brazil 2012−2023 APC= 0.43
China Observed point, AAPC= 1.29
China 1990−1997 APC= 3.69*
China 1997−2011 APC= −0.29
China 2011−2023 APC= 1.78*
India Observed point, AAPC= 7.06
India 1990−1995 APC= 4.19*
India 1995−2001 APC= 14.85*
India 2001−2008 APC= 10.79*
India 2008−2023 APC= 3.37*
Mexico Observed point, AAPC= 1.79
Mexico 1990−1995 APC= 2.89*
Mexico 1995−2000 APC= 7.10*
Mexico 2000−2018 APC= 1.18*
Mexico 2018−2023 APC= −2.17*
Nigeria Observed point, AAPC= 1.16
Nigeria 1990−1994 APC= 1.52
Nigeria 1994−2000 APC= 3.20
Nigeria 2000−2020 APC= −0.40*
Nigeria 2020−2023 APC= 7.27*
Pakistan Observed point, AAPC= 4.6
Pakistan 1990−2000 APC= 2.46*
Pakistan 2000−2014 APC= 6.94*
Pakistan 2014−2019 APC= 1.80
Pakistan 2019−2023 APC= 5.51*
Russian Federation Observed point, AAPC= 2.22
Russian Federation 1990−1993 APC= 4.26*
Russian Federation 1993−2005 APC= 0.25
Russian Federation 2005−2014 APC= 5.40*
Russian Federation 2014−2023 APC= 1.07*
United States of America Observed point, AAPC= 3.9
United States of America 1990−2000 APC= 6.30*
United States of America 2000−2011 APC= 4.12*
United States of America 2011−2017 APC= 2.86*
United States of America 2017−2023 APC= 0.62*
Bangladesh Observed point, AAPC= 5.13
Bangladesh 1990−1996 APC= 3.28*
Bangladesh 1996−2001 APC= 5.96*
Bangladesh 2001−2019 APC= 4.65*
Bangladesh 2019−2023 APC= 9.12*

Figure S11

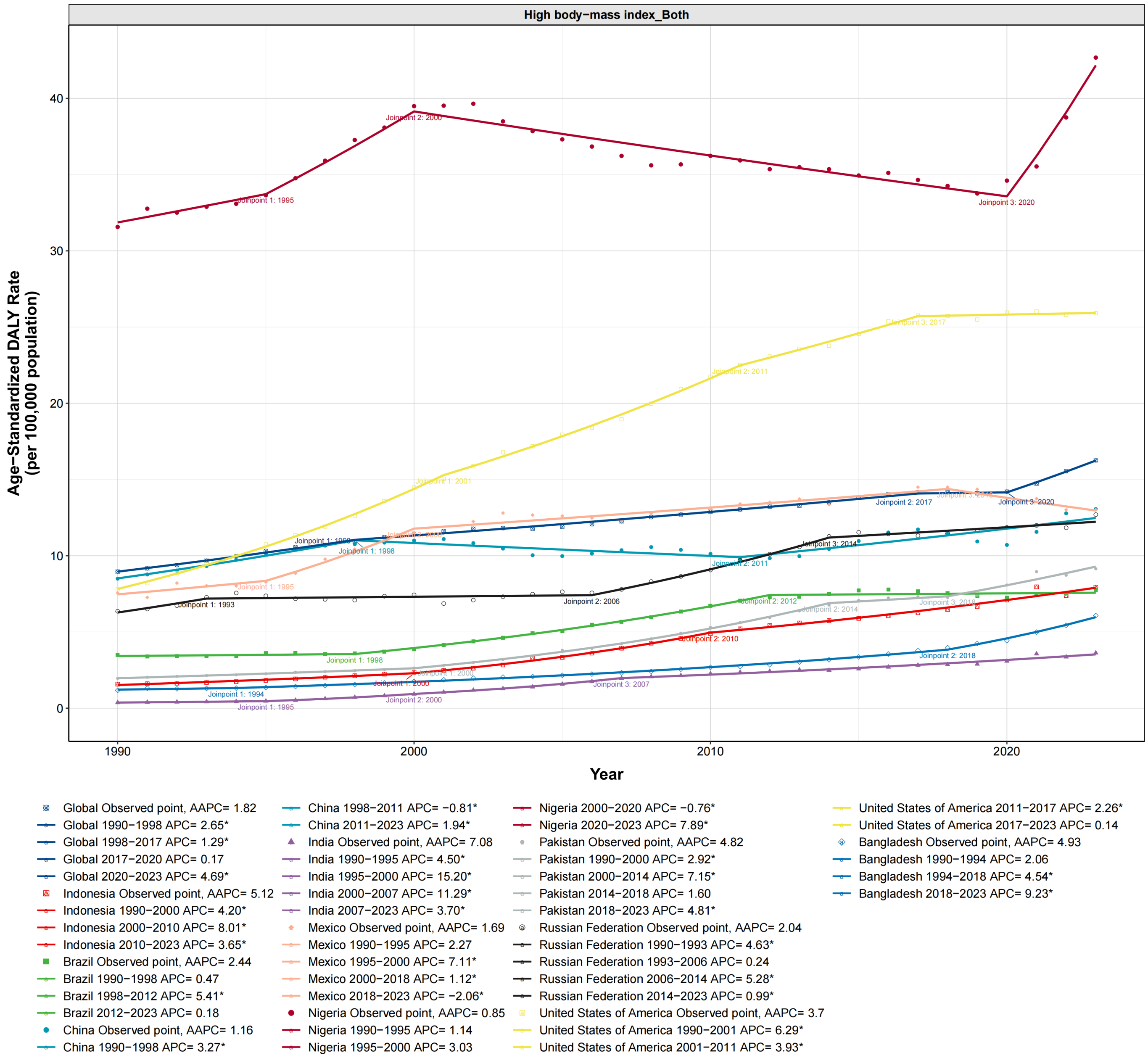

High body−mass index_Both
Age−Standardized DALY Rate
(per 100,000 population)
Year
Global Observed point, AAPC= 1.82
Global 1990−1998 APC= 2.65*
Global 1998−2017 APC= 1.29*
Global 2017−2020 APC= 0.17
Global 2020−2023 APC= 4.69*
Indonesia Observed point, AAPC= 5.12
Indonesia 1990−2000 APC= 4.20*
Indonesia 2000−2010 APC= 8.01*
Indonesia 2010−2023 APC= 3.65*
Brazil Observed point, AAPC= 2.44
Brazil 1990−1998 APC= 0.47
Brazil 1998−2012 APC= 5.41*
Brazil 2012−2023 APC= 0.18
China Observed point, AAPC= 1.16
China 1990−1998 APC= 3.27*
China 1998−2011 APC= −0.81*
China 2011−2023 APC= 1.94*
India Observed point, AAPC= 7.08
India 1990−1995 APC= 4.50*
India 1995−2000 APC= 15.20*
India 2000−2007 APC= 11.29*
India 2007−2023 APC= 3.70*
Mexico Observed point, AAPC= 1.69
Mexico 1990−1995 APC= 2.27
Mexico 1995−2000 APC= 7.11*
Mexico 2000−2018 APC= 1.12*
Mexico 2018−2023 APC= −2.06*
Nigeria Observed point, AAPC= 0.85
Nigeria 1990−1995 APC= 1.14
Nigeria 1995−2000 APC= 3.03
Nigeria 2000−2020 APC= −0.76*
Nigeria 2020−2023 APC= 7.89*
Pakistan Observed point, AAPC= 4.82
Pakistan 1990−2000 APC= 2.92*
Pakistan 2000−2014 APC= 7.15*
Pakistan 2014−2018 APC= 1.60
Pakistan 2018−2023 APC= 4.81*
Russian Federation Observed point, AAPC= 2.04
Russian Federation 1990−1993 APC= 4.63*
Russian Federation 1993−2006 APC= 0.24
Russian Federation 2006−2014 APC= 5.28*
Russian Federation 2014−2023 APC= 0.99*
United States of America Observed point, AAPC= 3.7
United States of America 1990−2001 APC= 6.29*
United States of America 2001−2011 APC= 3.93*
United States of America 2011−2017 APC= 2.26*
United States of America 2017−2023 APC= 0.14
Bangladesh Observed point, AAPC= 4.93
Bangladesh 1990−1994 APC= 2.06
Bangladesh 1994−2018 APC= 4.54*
Bangladesh 2018−2023 APC= 9.23*

Figure S12

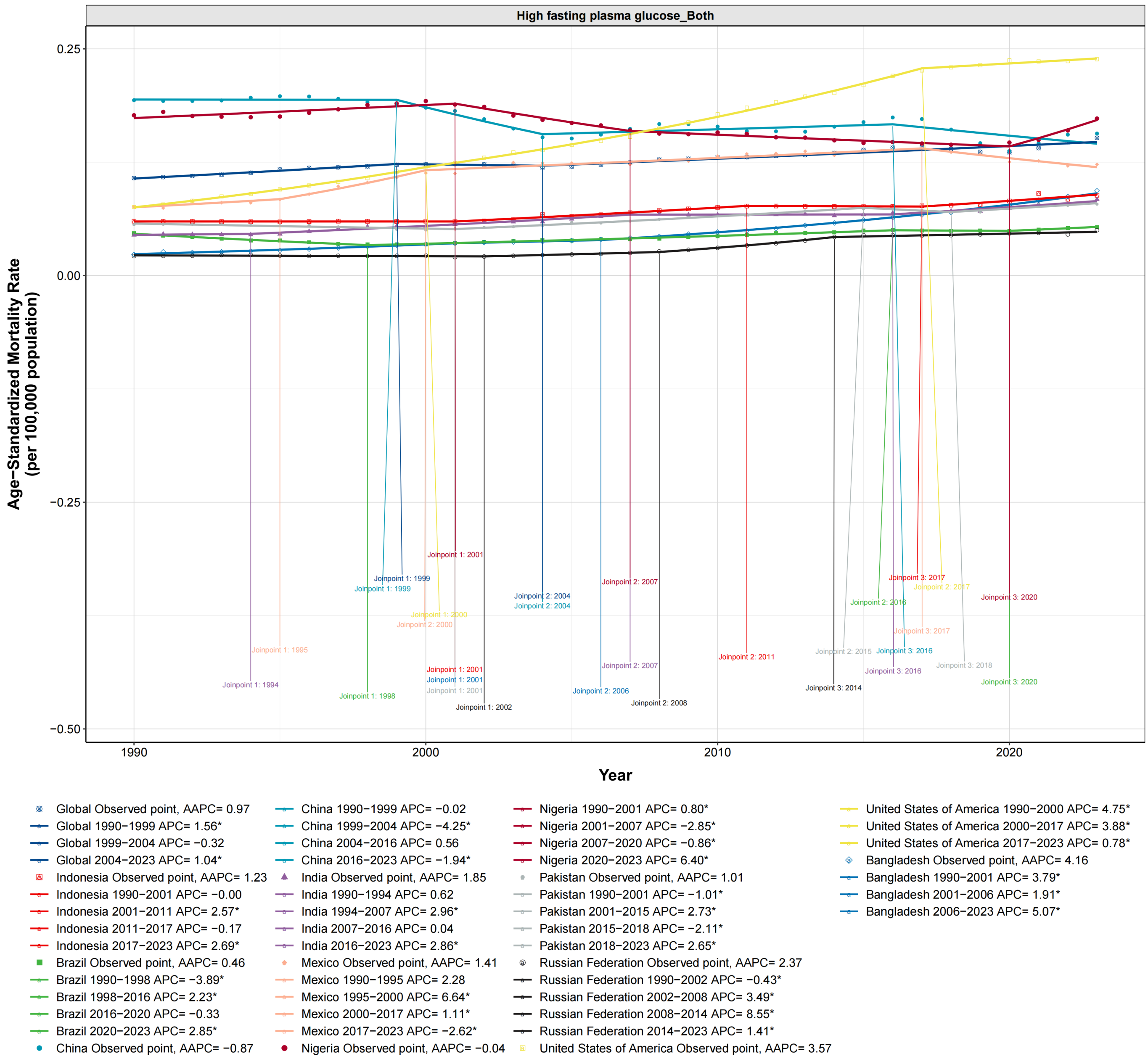
High fasting plasma glucose_Both
Age−Standardized Mortality Rate (per 100,000 population)
0.25
0.00
−0.25
−0.50
1990
2000
2010
2020
Year
Joinpoint 1: 1994
Joinpoint 1: 1995
Joinpoint 1: 1998
Joinpoint 1: 1999
Joinpoint 1: 1999
Joinpoint 1: 2000
Joinpoint 2: 2000
Joinpoint 1: 2001
Joinpoint 1: 2001
Joinpoint 1: 2001
Joinpoint 1: 2001
Joinpoint 1: 2002
Joinpoint 2: 2004
Joinpoint 2: 2004
Joinpoint 2: 2006
Joinpoint 2: 2007
Joinpoint 2: 2007
Joinpoint 2: 2008
Joinpoint 2: 2011
Joinpoint 3: 2014
Joinpoint 2: 2015
Joinpoint 2: 2016
Joinpoint 3: 2016
Joinpoint 3: 2016
Joinpoint 3: 2017
Joinpoint 2: 2017
Joinpoint 3: 2017
Joinpoint 3: 2018
Joinpoint 3: 2020
Joinpoint 3: 2020
Global Observed point, AAPC= 0.97
Global 1990−1999 APC= 1.56*
Global 1999−2004 APC= −0.32
Global 2004−2023 APC= 1.04*
Indonesia Observed point, AAPC= 1.23
Indonesia 1990−2001 APC= −0.00
Indonesia 2001−2011 APC= 2.57*
Indonesia 2011−2017 APC= −0.17
Indonesia 2017−2023 APC= 2.69*
Brazil Observed point, AAPC= 0.46
Brazil 1990−1998 APC= −3.89*
Brazil 1998−2016 APC= 2.23*
Brazil 2016−2020 APC= −0.33
Brazil 2020−2023 APC= 2.85*
China Observed point, AAPC= −0.87
China 1990−1999 APC= −0.02
China 1999−2004 APC= −4.25*
China 2004−2016 APC= 0.56
China 2016−2023 APC= −1.94*
India Observed point, AAPC= 1.85
India 1990−1994 APC= 0.62
India 1994−2007 APC= 2.96*
India 2007−2016 APC= 0.04
India 2016−2023 APC= 2.86*
Mexico Observed point, AAPC= 1.41
Mexico 1990−1995 APC= 2.28
Mexico 1995−2000 APC= 6.64*
Mexico 2000−2017 APC= 1.11*
Mexico 2017−2023 APC= −2.62*
Nigeria Observed point, AAPC= −0.04
Nigeria 1990−2001 APC= 0.80*
Nigeria 2001−2007 APC= −2.85*
Nigeria 2007−2020 APC= −0.86*
Nigeria 2020−2023 APC= 6.40*
Pakistan Observed point, AAPC= 1.01
Pakistan 1990−2001 APC= −1.01*
Pakistan 2001−2015 APC= 2.73*
Pakistan 2015−2018 APC= −2.11*
Pakistan 2018−2023 APC= 2.65*
Russian Federation Observed point, AAPC= 2.37
Russian Federation 1990−2002 APC= −0.43*
Russian Federation 2002−2008 APC= 3.49*
Russian Federation 2008−2014 APC= 8.55*
Russian Federation 2014−2023 APC= 1.41*
United States of America Observed point, AAPC= 3.57
United States of America 1990−2000 APC= 4.75*
United States of America 2000−2017 APC= 3.88*
United States of America 2017−2023 APC= 0.78*
Bangladesh Observed point, AAPC= 4.16
Bangladesh 1990−2001 APC= 3.79*
Bangladesh 2001−2006 APC= 1.91*
Bangladesh 2006−2023 APC= 5.07*

Figure S13

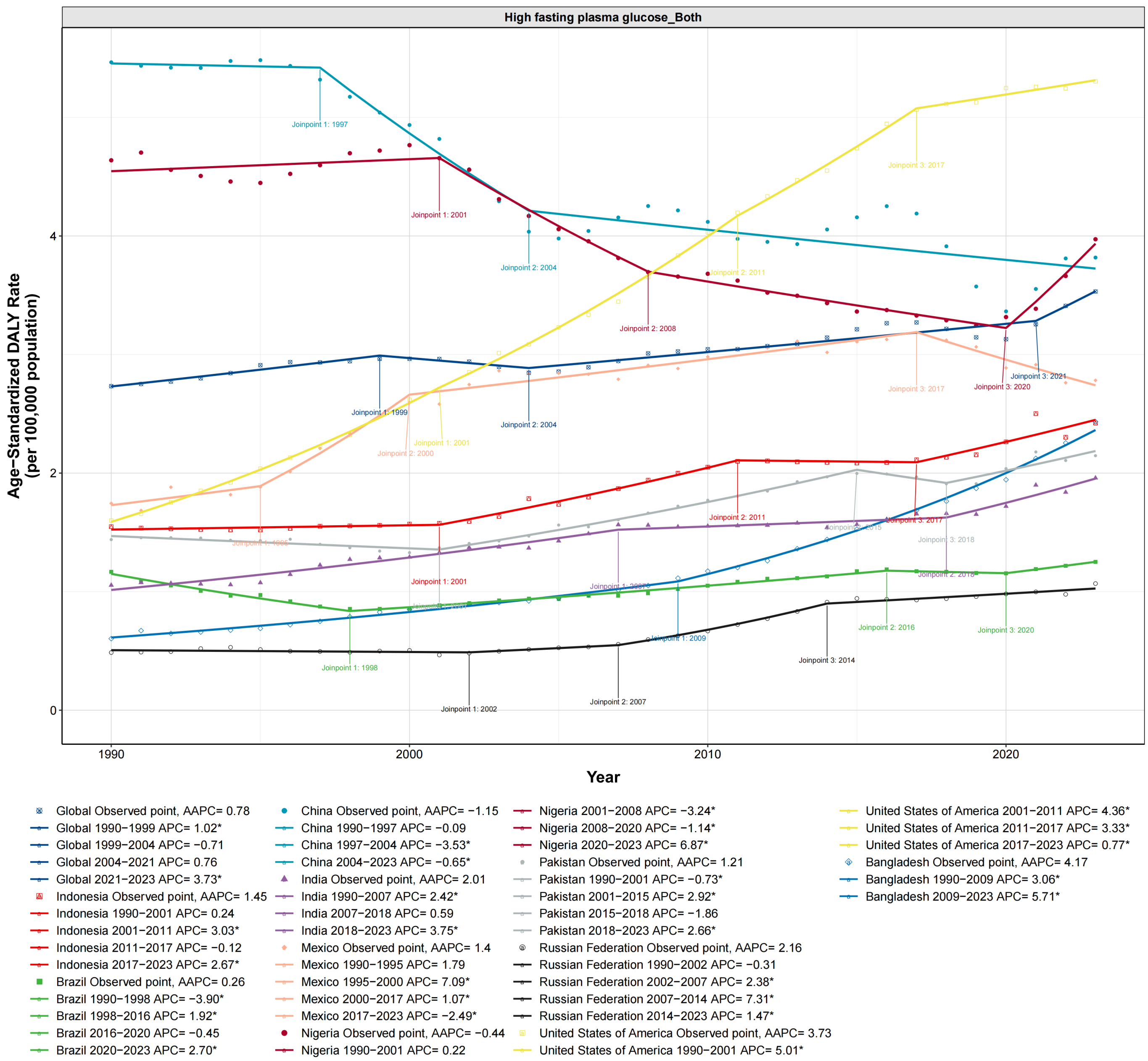

High fasting plasma glucose_Both
Age−Standardized DALY Rate (per 100,000 population)
Year
Global Observed point, AAPC= 0.78
Global 1990−1999 APC= 1.02*
Global 1999−2004 APC= −0.71
Global 2004−2021 APC= 0.76
Global 2021−2023 APC= 3.73*
Indonesia Observed point, AAPC= 1.45
Indonesia 1990−2001 APC= 0.24
Indonesia 2001−2011 APC= 3.03*
Indonesia 2011−2017 APC= −0.12
Indonesia 2017−2023 APC= 2.67*
Brazil Observed point, AAPC= 0.26
Brazil 1990−1998 APC= −3.90*
Brazil 1998−2016 APC= 1.92*
Brazil 2016−2020 APC= −0.45
Brazil 2020−2023 APC= 2.70*
China Observed point, AAPC= −1.15
China 1990−1997 APC= −0.09
China 1997−2004 APC= −3.53*
China 2004−2023 APC= −0.65*
India Observed point, AAPC= 2.01
India 1990−2007 APC= 2.42*
India 2007−2018 APC= 0.59
India 2018−2023 APC= 3.75*
Mexico Observed point, AAPC= 1.4
Mexico 1990−1995 APC= 1.79
Mexico 1995−2000 APC= 7.09*
Mexico 2000−2017 APC= 1.07*
Mexico 2017−2023 APC= −2.49*
Nigeria Observed point, AAPC= −0.44
Nigeria 1990−2001 APC= 0.22
Nigeria 2001−2008 APC= −3.24*
Nigeria 2008−2020 APC= −1.14*
Nigeria 2020−2023 APC= 6.87*
Pakistan Observed point, AAPC= 1.21
Pakistan 1990−2001 APC= −0.73*
Pakistan 2001−2015 APC= 2.92*
Pakistan 2015−2018 APC= −1.86
Pakistan 2018−2023 APC= 2.66*
Russian Federation Observed point, AAPC= 2.16
Russian Federation 1990−2002 APC= −0.31
Russian Federation 2002−2007 APC= 2.38*
Russian Federation 2007−2014 APC= 7.31*
Russian Federation 2014−2023 APC= 1.47*
United States of America Observed point, AAPC= 3.73
United States of America 1990−2001 APC= 5.01*
United States of America 2001−2011 APC= 4.36*
United States of America 2011−2017 APC= 3.33*
United States of America 2017−2023 APC= 0.77*
Bangladesh Observed point, AAPC= 4.17
Bangladesh 1990−2009 APC= 3.06*
Bangladesh 2009−2023 APC= 5.71*

Figure S14
